# Diphtheria-tetanus-acellular pertussis vaccine safety in children under 7 years: a post-marketing analysis of the U.S. vaccine adverse event reporting system

**DOI:** 10.3389/fcimb.2026.1733777

**Published:** 2026-02-02

**Authors:** Meishen Liu, Xueying Zhou, Linshu Guan, Hening Sun, Zhaohui Bai, Yalin Xi, Xinkuo Zheng

**Affiliations:** 1 Department of Pharmacy, The Second Affiliated Hospital of Dalian Medical University, Dalian, Liaoning, China; 2 Department of Pharmacy, Beijing Anzhen Hospital, Capital Medical University, Beijing, China; 3 Department of Pharmacy, Central Hospital of Dalian University of Technology, Dalian, Liaoning, China

**Keywords:** diphtheria-tetanus-acellular pertussis vaccines, DTaP, pharmacovigilance, post-marketing safety surveillance, vaccine adverse event reporting system

## Abstract

**Objectives:**

Although Diphtheria-Tetanus-acellular Pertussis (DTaP) vaccines have been used in the U.S. for decades and have extensive safety records, a comprehensive post-marketing assessment for all available types is still needed. This study leveraged the Vaccine Adverse Event Reporting System (VAERS) database to evaluate adverse events following immunization (AEFI) and analyze potential associations with vaccine administration.

**Methods:**

We extracted all reports of adverse events (AEs) following DTaP vaccination from the VAERS database for the period 1990 to May 2025. Our analysis included descriptive statistics to summarize patient demographics and clinical features, and disproportionality methods to identify potential safety signals.

**Results:**

During the study period, the VAERS database documented 57,341 children under 7 years who received DTaP vaccines, corresponding to 57,368 administered doses and 193,955 adverse event (AE) reports. AE reporting was more frequent in males (52.46%) than females (45.07%), with more than half of the cases (50.44%) involving children under 2 years old. The most common clinical outcomes were recovery (62.53%) and hospitalization (10.29%). Most AEs (89.61%) occurred within 0–30 days after vaccination, with a median onset time of 1.0 days. Infanrix (37.95%) and Daptacel (25.04%) were the most frequently reported vaccine types. Disproportionality analysis detected 158 positive AE signals across 24 system organ classes (SOCs). Among all SAEs, pyrexia (ROR = 1.01) was the most frequently reported, followed by convulsion (ROR = 1.82) and vomiting (ROR = 1.05). The most common signals for non-SAEs included injection site erythema (ROR = 3.79), injection site swelling (ROR = 3.00), and erythema (ROR = 3.03).

**Conclusions:**

This post-marketing surveillance indicates that most reported AEs were non-serious and occurred within 30 days following vaccination. These findings support the known safety profile of DTaP vaccines and highlight identifiable timing patterns of AEs, which may help inform monitoring strategies and benefit-risk assessments after immunization.

## Introduction

Pertussis incidence in the U.S. has significantly decreased since the adoption of tetanus, diphtheria toxoids, and whole-cell pertussis vaccines during the 1940s (Loiacono et al., 2021). Beginning in the 1990s, replacement occurred with acellular pertussis (DTaP) vaccines (Centers for Disease Control and Prevention, 1992a). Current U.S. DTaP-containing vaccines effectively prevent pertussis in most children, demonstrating approximately 97.7% efficacy after four doses (Liang et al., 2018). According to Centers for Disease Control and Prevention (CDC) pertussis surveillance data, 3,044 pertussis cases were reported in 2022. Children aged <1 year accounted for 344 cases, with an incidence rate of 18.1 per 100,000. The 1–6-year age group had 832 cases and an incidence of 3.7 per 100,000 (Centers for Disease Control and Prevention, 2024). In contrast, 2023 saw a substantial rise to 7,063 reported cases nationwide. Among these, children <1 year represented 845 cases, yielding an incidence of 46.3 per 100,000. The 1–6-year cohort reported 2,095 cases and an incidence of 9.2 per 100,000 (Centers for Disease Control and Prevention, 2025). These findings indicate a marked upward trend in pertussis incidence.

The Advisory Committee on Immunization Practices (ACIP) recommends a DTaP vaccination schedule comprising a primary series at 2, 4, and 6 months of age, followed by an initial booster at 15–18 months and a subsequent booster at 4–6 years (Liang et al., 2018). Analysis of U.S. National Immunization Survey-Child (NIS-Child) data from 2013 to 2017 demonstrated consistently high coverage exceeding 93.7% for ≥3 DTaP doses among children aged 19–35 months. Coverage peaked at 95.0% in 2015, subsequently declined significantly to 93.7% in 2016, then rebounded slightly to 94.0% in 2017. In contrast, ≥4-dose coverage remained substantially lower throughout the period, ranging narrowly from 83.1% in 2013 to 84.6% in 2015. This persistent gap underscored ongoing challenges in delivering booster vaccinations (Hill et al., 2018).

The development of DTaP vaccines in the U.S. commenced with Acel-Imune^®^ (Lederle Laboratories/Wyeth), the first licensed acellular formulation approved in 1991, which was subsequently discontinued in 2002 due to corporate restructuring. Sanofi Pasteur introduced Tripedia^®^ in 1996 as the inaugural monovalent acellular DTaP vaccine, but production ceased in 2011 following strategic portfolio optimization. Concurrently, Infanrix^®^ (GlaxoSmithKline Biologicals), approved in 1997, remains commercially active today as both a standalone vaccine and a component of combination products (e.g., Kinrix^®^). Certiva^®^ (Sanofi Pasteur) experienced a complex trajectory: initially marketed in 1998 for pediatric primary immunization but withdrawn in 2000; reintroduced in 2018 for booster doses before final discontinuation in 2020. Daptacel^®^ (Sanofi Pasteur), licensed in 2002, persists in current use exclusively within multivalent vaccines such as Pentacel^®^. Non-branded formulations referenced in surveillance data typically denote generic equivalents or investigational lots without formal commercialization. This dynamic landscape reflects industry consolidation, evolving immunization strategies favoring combination vaccines, and manufacturer-driven decisions to sunset legacy products—culminating in the contemporary dominance of Infanrix^®^ and Daptacel^®^-based formulations (Centers for Disease Control and Prevention, 1992a; Centers for Disease Control and Prevention, 1992b; Centers for Disease Control and Prevention, 1996; Centers for Disease Control and Prevention, 1997; Klein, 2014).

Ongoing surveillance of AEs following immunization remains a critical component of vaccine safety. An adverse event following immunization (AEFI), defined by the Council for International Organizations of Medical Sciences (CIOMS) as any medically untoward occurrence temporally linked to vaccination without confirmed causality, constitutes a key pharmacovigilance parameter. This operational definition emphasizes temporal association over established causation in preliminary safety evaluations (Ujiie et al., 2021; Marshall et al., 2022). Following three decades of global deployment, DTaP vaccines have achieved extensive coverage across diverse populations, particularly pediatric cohorts. Given substantial exposure in these vulnerable groups, continuous safety surveillance is essential (Masseria et al., 2015). A 2018 study revealed that the most frequently documented DTaP adverse reactions include injection site erythema, pyrexia, injection site swelling, and injection site warmth (Moro et al., 2018). Currently, a comprehensive pharmacovigilance analysis encompassing all U.S.-marketed DTaP vaccines remains lacking, a gap this study aims to address.

The VAERS is a foundational piece of U.S. pharmacovigilance infrastructure, operating as a passive surveillance system that encompasses the entire national population (Moro et al., 2018). Its population-wide scope facilitates near-real-time identification of potential vaccine safety concerns by detecting disproportional adverse event reporting patterns (Chen et al., 1994). A safety signal in pharmacoepidemiology refers to an emerging potential link between a medical product and an AE, detected through comprehensive analysis of pharmacovigilance data. Disproportionality analysis continues to be the benchmark technique for detecting rare and idiosyncratic adverse drug reactions that call for immediate attention. Within this framework, our investigation conducts comprehensive data mining of VAERS reports involving children under 7 years across multiple DTaP vaccine formulations. This systematic profiling of post-vaccination safety establishes evidence-based risk stratification to guide clinical vaccination strategies.

## Materials and methods

### Data source

This study employed data from the VAERS database, a U.S. national surveillance system co-managed by the CDC and the Food and Drug Administration (FDA) (Chen et al., 1994). Since 1990, VAERS has remained an essential component of the U.S. pharmacovigilance system for monitoring the safety of vaccines after they have been approved. Its key aims are to detect new, unusual, or rare AEs and monitor trends in known ones, as well as to identify patient-specific risk factors along with evaluating the safety of newly licensed vaccines (Centers for Disease Control and Prevention (CDC), 2024). Adverse event symptoms are documented within the system using standardized MedDRA (version 27.1) Preferred Term (PT) codes, with each VAERS case report permitting documentation of up to five distinct clinical manifestations. The assignment of PTs to SOCs was conducted as part of the analytical methodology (Brown, 2004; Dong et al., 2025).

Within the scope of this study, VAERS reports related to DTaP vaccines were systematically examined for the period between January 1, 1990 and May 31, 2025. Given that young children often receive multiple vaccines simultaneously, DTaP was treated as one of the possible exposures in our signal detection analysis, rather than the sole factor. The dataset encompassed all submissions for the following formulations: DTAP (ACEL-IMUNE), DTAP (CERTIVA), DTAP (DAPTACEL), DTAP (INFANRIX), DTAP (TRIPEDIA), DTAP (NO BRAND NAME). To capture all reported AEs, the analysis was conducted without applying any geographic or clinical filters. Duplicate data is very limited, and we have performed deduplication on such data. For demographic parameters, we selectively included only patients under 7 years who received DTaP vaccination to specifically evaluate its safety profile, while applying no restrictions to other demographic variables. Additionally, we conducted subgroup analyses by age (<2 years, 2–5 years, and 5–7 years) and gender for an in-depth analysis of DTaP safety. Serious adverse events (SAEs) and fatal outcomes were also subject to dedicated analysis.

### Descriptive analysis

We performed a descriptive analysis of DTaP-associated adverse events using the collected reports, stratifying the data by gender, age, primary clinical outcome (including death, disability, hospitalization, life-threatening events, prolonged hospitalization, and recovery), and time of onset. Additionally, we categorized all vaccines by product name, manufacturer, and dose number, analyzing annual reports from 1990 through May 2025 for each.

### Statistical analysis

Our pharmacovigilance framework incorporates four disproportionality analyses: Proportional Reporting Ratio (PRR) (Evans et al., 2001), Reporting Odds Ratio (ROR) (Rothman et al., 2004), Bayesian Confidence Propagation Neural Network (BCPNN) (Bate et al., 1998), and Multi-Item Gamma Poisson Shrinker (MGPS) (Heo and Jung, 2020). Each algorithm exhibits unique advantages: PRR achieves high specificity; ROR addresses reporting bias in low-frequency events more effectively than PRR; BCPNN facilitates Bayesian integration of multi-source data; while MGPS outperforms BCPNN in detecting signals from rare occurrences. Integration of these complementary methodologies mitigates individual limitations through cross-validation. To avoid oversight, a signal was considered positive if it satisfied any one of the four aforementioned methods. Standardized 2×2 contingency tables (**Supplementary Table S1**) were applied universally, employing consistent computational formulas with predefined statistical criteria (**Supplementary Table S2**). Enhanced signal strength corresponds to increased probability of vaccine-adverse event associations. Following initial signal detection, the Benjamini-Hochberg (BH) procedure was applied to χ² test results from PRR to control the False Discovery Rate (FDR) and mitigate inflated Type I errors from multiple hypothesis testing. A potential AE signal was considered statistically significant only if its Benjamini-Hochberg (BH) adjusted *p*-value met the predefined false discovery rate (FDR) threshold of Q = 0.05. Vaccine-event pairs meeting this threshold were classified as positive disproportionality signals. All analyses were performed using R software (v4.4.2) and Microsoft Excel 2021.

### Designated medical event list-based safety monitoring

In 2016, the European Medicines Agency (EMA) established a key pharmacovigilance tool, which includes a list of 62 PTs designated as Designated Medical Events (Liu et al., 2023). These represent inherently serious medical conditions demonstrating frequent medication-associated relationships. This curated framework enhances signal detection efficiency by prioritizing adverse events requiring urgent investigation, serving as a critical safeguard against surveillance gaps in pharmacovigilance systems. Our investigation specifically employed systematic Designated Medical Event screening to evaluate clinically significant safety profiles associated with DTaP vaccines. We cross-referenced all detected PTs against the European Medicines Agency-designated list of 62 Designated Medical Events and performed disproportionality analysis on the matched subset of Designated Medical Event -related PT.

## Results

### Demographic and clinical profile of AEs

This investigation assessed 57,341 DTaP-vaccinated recipients aged < 7 years, with 57,368 AEs documented. Analysis indicated a reduced incidence of AEs among females (45.0%) compared with males (52.4%). Stratification by age identified the largest affected subgroup as children under 2 years (30.33%). Among patients manifesting AEs post-DTaP vaccination (excluding those with undocumented clinical outcomes), recovery constituted the predominant clinical outcome (62.53%), followed by hospitalization attributable to the AEs (10.29%). Temporal assessment revealed that 89.61% of patients exhibited AEs within 0–30 days post-vaccination. Within this temporal window, 37,196 cases demonstrated an induction time of ≤ 1 day, accounting for approximately 64.87% of all AEs. For cases with documentation, the time to onset (days) presented a mean (SD) of 22.7 (203) and a median (min – max) of 1.00 (0 – 14,100) (**Table 1**).

**Table 1:** Characteristics of AE reports associated with DTaP vaccines from VAERS between 1990 and 2025.

Characteristics	Number (%)
Total	57,341 (100%)
Gender	
Female	25,842 (45.07%)
Male	30,083 (52.46%)
N/A	1,416 (2.47%)
Age (year)	
< 2	28,922 (50.44%)
≥ 2 and < 5	17,390 (30.33%)
≥ 5 and < 7	11,029 (19.23%)
Primary clinical outcome	
Died	854 (1.49%)
Disability	912 (1.59%)
Hospitalized	5,898 (10.29%)
Life threatening	1,088 (1.90%)
Prolonged hospitalization	278 (0.48%)
Recovered	35,857 (62.53%)
N/A	18,136 (31.63%)
Onset time (day)	
< 30	51,382 (89.61%)
≥ 30 and < 60	479 (0.84%)
≥ 60 and < 90	170 (0.30%)
≥ 90 and < 120	107 (0.19%)
≥ 120 and < 150	54 (0.09%)
≥ 150 and < 180	44 (0.08%)
≥ 180 and < 360	196 (0.34%)
≥ 360	780 (1.36%)
N/A	4,129 (7.20%)

AE, adverse event; DTaP, diphtheria, tetanus, and acellular pertussis; VAERS, vaccine adverse event reporting system; N/A, not available.

**Figure 1:**
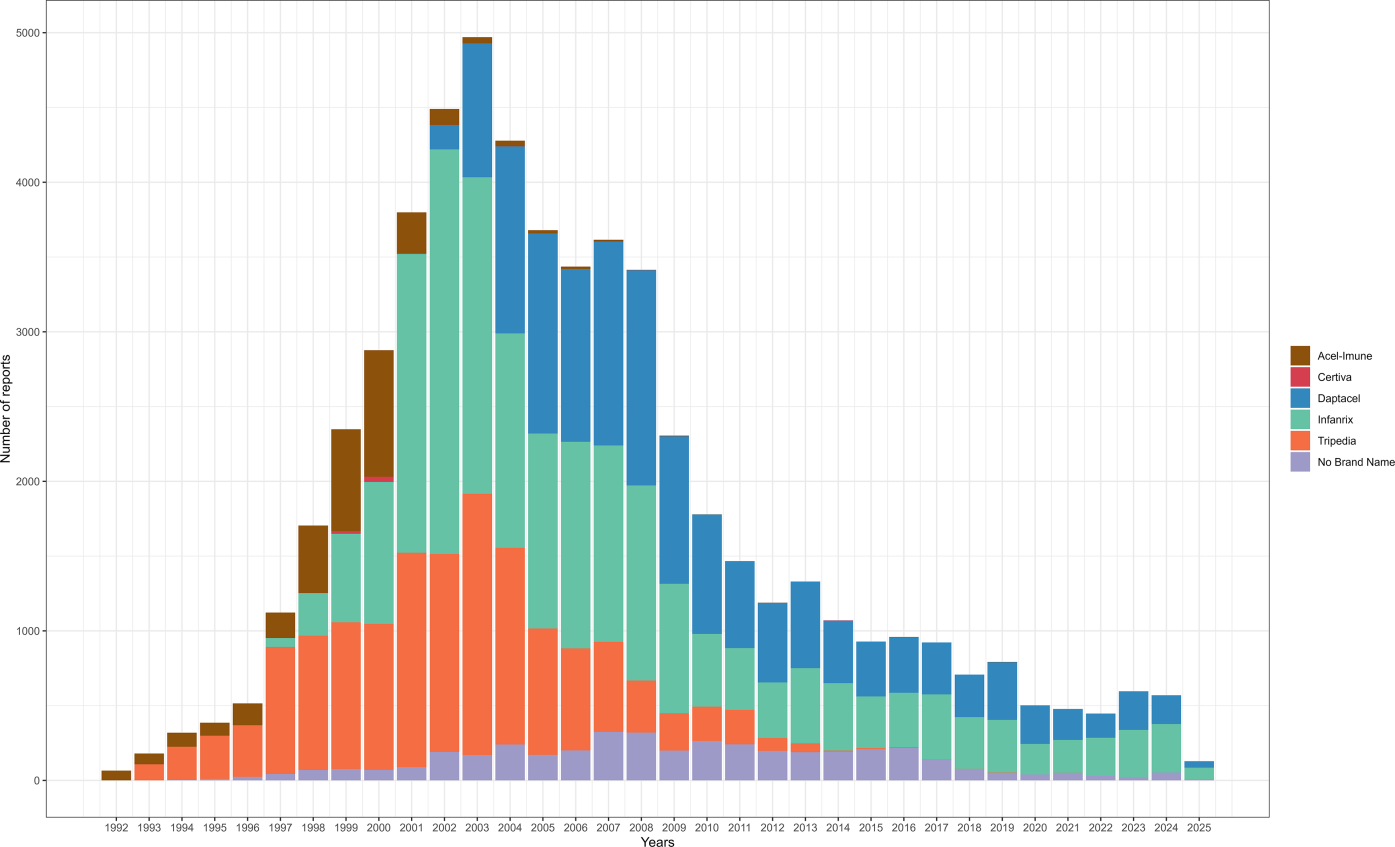
Number of reports per vaccine and per year of the DTaP vaccines. DTaP, diphtheria, tetanus, and acellular pertussis.

This study encompassed 57,341 patients receiving 57,368 DTaP vaccine doses. Infanrix was the most frequently administered DTaP vaccine (37.95%). By manufacturer, Sanofi Pasteur accounted for over one-third of all doses (38.46%), followed by GlaxoSmithKline Biologicals (25.48%). Regarding vaccination frequency, five doses constituted the most common regimen (30.22%), with four doses being the next most frequent (23.77%) (**Table 2**). The annual count of reported AEs per DTaP vaccine is presented in **Figure 1**.

**Table 2:** Characteristics of DTaP vaccines from VAERS between 1990 and 2025.

Characteristics	Number (%)
Total vaccines	57,368 (100%)
Vaccine name	
Acel-Imune	3,140 (5.47%)
Certiva	66 (0.12%)
Daptacel	14,366 (25.04%)
Infanrix	21,773 (37.95%)
Tripedia	13,845 (24.13%)
No Brand Name	4,178 (7.28%)
Vaccine manufacturer	
Baxter Healthcare Corp.	8 (<0.01%)
Connaught Laboratories	6,150 (10.72%)
GlaxoSmithKline Biologicals	14,617 (25.48%)
North American Vaccines	58 (0.10%)
Pfizer\Wyeth	3,140 (5.47%)
Sanofi Pasteur	22,061 (38.46%)
SmithKline Beecham	7,156 (12.47%)
N/A	4,178 (7.28%)
Administration dose	
1	6,510 (11.35%)
2	5,262 (9.17%)
3	4,030 (7.02%)
4	13,637 (23.77%)
5	17,338 (30.22%)
6	504 (0.88%)
7+	35 (0.06%)
N/A	10,052 (17.52%)

DTaP, diphtheria, tetanus, and acellular pertussis; VAERS, vaccine adverse event reporting system; N/A, not available.

### Disproportionality analysis

**Figure 2:**
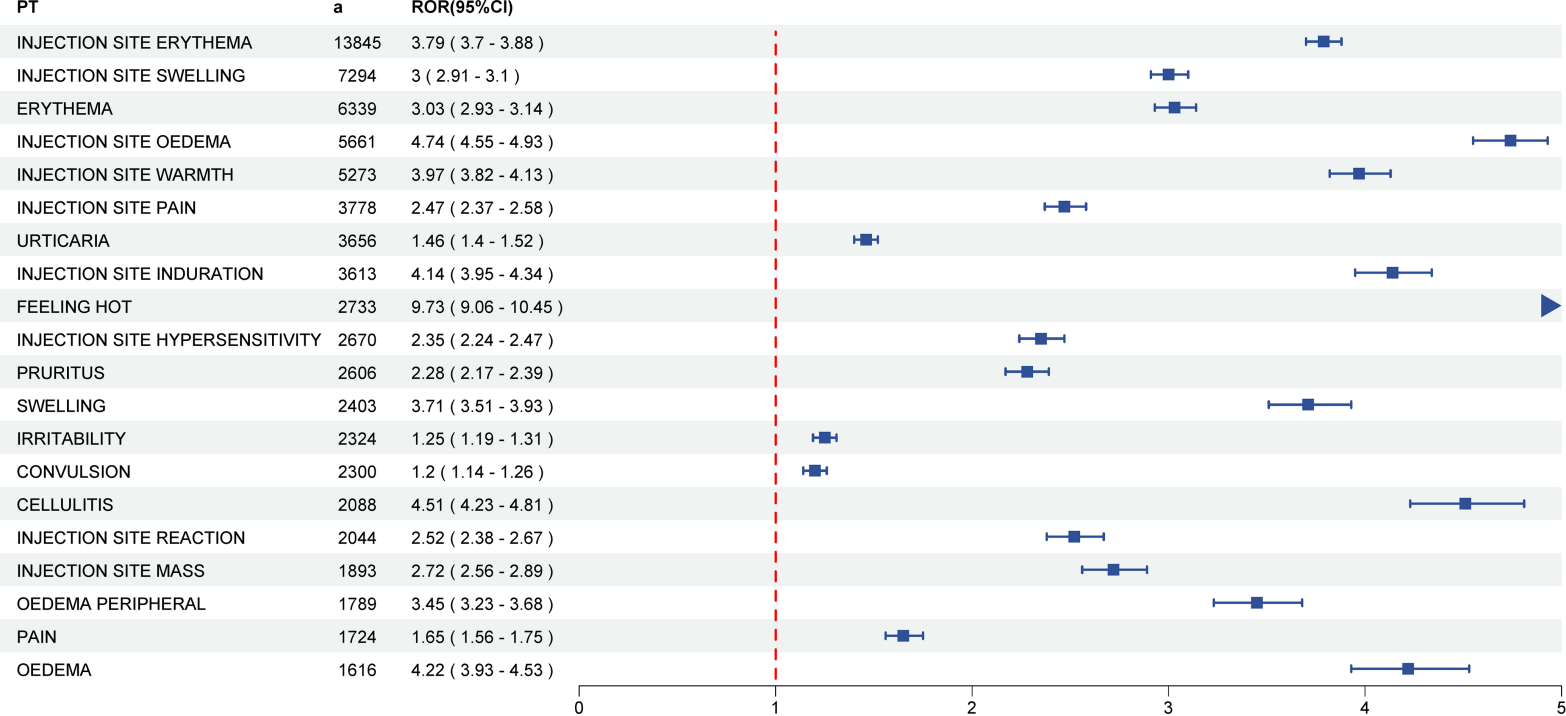
The forest plot of top twenty DTaP vaccine-related positive PT signals. DTaP, diphtheria, tetanus, and acellular pertussis; PT, preferred term; ROR, reporting odds ratio.

Analysis of VAERS reports in patients under 7 years old identified 3,554 DTaP vaccine-associated PTs across 27 SOCs. Pharmacovigilance assessment using four methods yielded 158 positive PT signals spanning 24 SOCs. The five most frequent PTs for positive AE signals were injection site erythema (a = 13,845, ROR = 3.79, PRR = 3.59, EBGM = 2.31, IC = 1.21), injection site swelling (a = 7,294, ROR = 3.00, PRR = 2.93, EBGM = 2.08, IC = 1.05), erythema (a = 6,339, ROR = 3.03, PRR = 2.97, EBGM = 2.09, IC = 1.06), injection site oedema (a = 5,661, ROR = 4.74, PRR = 4.63, EBGM = 2.61, IC = 1.38) and injection site warmth (a = 5,273, ROR = 3.97, PRR = 3.89, EBGM = 2.41, IC = 1.27). **Figure 2** presents the top twenty positive PT signals related to DTaP vaccines, while **Supplementary Table S3** lists all identified positive PT signals.

The 158 positive PT signals underwent BH correction (**Supplementary Table S3**). Results revealed that 15 PTs were reclassified as non-positive signals, namely post-tussive vomiting (a = 48, *p*-adjust = 0.052), visual disturbance (a = 28, *p*-adjust = 0.055), skin burning sensation (a = 15, *p*-adjust = 0.051), asterixis (a = 13, *p*-adjust = 0.053), respiratory depression (a = 12, *p*-adjust = 0.061), nasal oedema (a = 7, *p*-adjust = 0.092), injection site dermatitis (a = 7, *p*-adjust = 0.063), asocial behaviour (a = 6, *p*-adjust = 0.067), piloerection (a = 4, *p*-adjust = 0.067), sensation of heaviness (a = 3, *p*-adjust = 0.117), wolff-parkinson-white syndrome (a = 3, *p*-adjust = 0.117), aplasia pure red cell (a = 3, *p*-adjust = 0.117), respiratory syncytial virus serology (a = 3, *p*-adjust = 0.117), hearing aid user (a = 3, *p*-adjust = 0.117), and injection site joint movement impairment (a = 3, *p*-adjust = 0.117). The remaining 143 PTs remained positive signals following BH correction.

**Figure 3:**
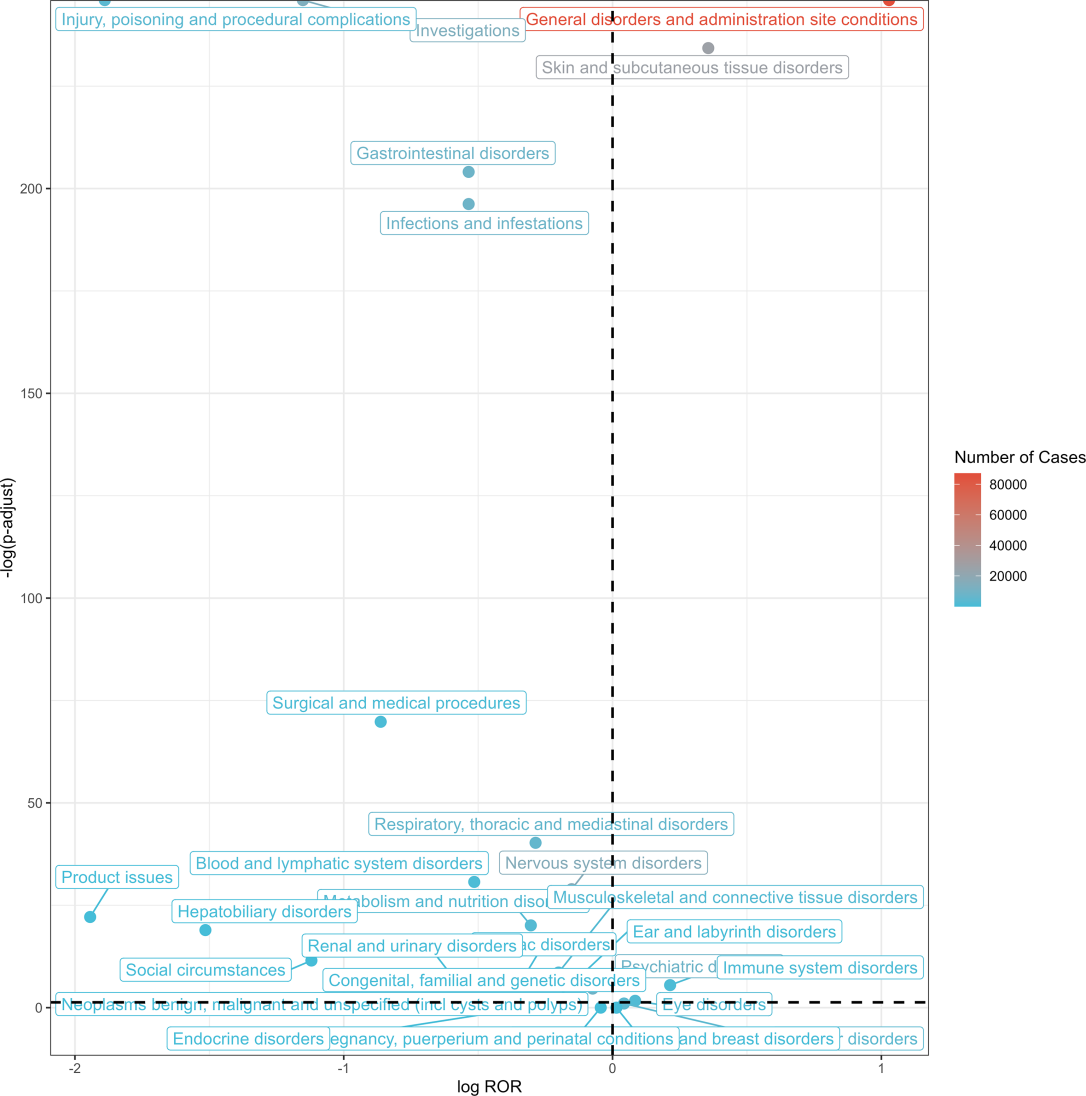
Volcano plot of SOCs corresponding to all reported AEs (X-axis: log (ROR); Y-axis, statistical significance, -log(p-adjust)). SOC, system organ classes; AEs, adverse events; ROR, reporting odds ratio.

The five most frequent SOCs were general disorders and administration site conditions (a = 87,112, ROR = 2.04, PRR = 1.57, EBGM = 1.40, IC = 0.49), skin and subcutaneous tissue disorders (a = 26,184, ROR = 1.28, PRR = 1.25, EBGM = 1.18, IC = 0.24), nervous system disorders (a = 15,856, ROR = 0.90, PRR = 0.91, EBGM = 0.93, IC = -0.11), investigations (a = 13,624, ROR = 0.45, PRR = 0.49, EBGM = 0.55, IC = -0.87) and infections and infestations (a = 8,323, ROR = 0.69, PRR = 0.71, EBGM = 0.75, IC = -0.41). **Figure 3** displays the association between signal strength (ROR) and statistical significance for SOC-level AEs associated with DTaP vaccination. Signal strengths of DTaP vaccine-related AEs at the SOC level are reported in **Supplementary Table S4**.

### Subgroup analysis

In pharmacovigilance, subgroup analysis mitigates the impact of confounding factors related to demographic characteristics and delivers a multi-dimensional perspective for evaluating drug safety.

#### PTs across sex groups

To examine the influence of gender on the side effects of DTaP vaccines, we conducted subgroup analyses stratified by gender. **Table 3** presents the top ten PTs meeting at least one of four statistical methods in both females and males.

**Table 3:** Signal strength of reports of DTaP vaccines at the PT level grouped by sex.

Sex	PT	a	ROR (95%Cl)	PRR (χ^2^)	EBGM (EBGM05)	IC (IC-2SD)
Female	Injection site erythema	6463	3.63 (3.51 - 3.76)	3.44 (5950.16)	2.26 (2.19)	1.17 (1.13)
	Injection site swelling	3318	2.87 (2.74 – 3.00)	2.80 (2213.19)	2.02 (1.94)	1.01 (0.95)
	Erythema	2811	2.96 (2.82 - 3.11)	2.90 (1977.65)	2.06 (1.97)	1.04 (0.98)
	Injection site warmth	2477	3.91 (3.69 - 4.14)	3.83 (2556.47)	2.38 (2.27)	1.25 (1.18)
	Injection site oedema	2453	4.60 (4.33 - 4.88)	4.50 (3024.60)	2.57 (2.44)	1.36 (1.29)
	Injection site induration	1791	3.96 (3.70 - 4.23)	3.90 (1882.49)	2.40 (2.27)	1.26 (1.18)
	Injection site pain	1756	2.42 (2.28 - 2.57)	2.39 (869.79)	1.84 (1.75)	0.88 (0.80)
	Urticaria	1531	1.34 (1.27 - 1.42)	1.34 (96.62)	1.25 (1.19)	0.32 (0.23)
	Feeling hot	1294	9.76 (8.79 - 10.83)	9.63 (2764.29)	3.37 (3.09)	1.75 (1.65)
	Injection site hypersensitivity	1249	2.17 (2.02 - 2.33)	2.15 (487.87)	1.72 (1.63)	0.79 (0.69)
Male	Injection site erythema	7042	3.75 (3.63 - 3.88)	3.57 (6669.86)	2.28 (2.21)	1.19 (1.14)
	Injection site swelling	3883	2.97 (2.85 - 3.10)	2.90 (2701.2)	2.04 (1.97)	1.03 (0.97)
	Erythema	3404	2.96 (2.83 - 3.10)	2.90 (2363.76)	2.04 (1.97)	1.03 (0.97)
	Injection site oedema	2968	4.65 (4.40 - 4.91)	4.54 (3625.87)	2.55 (2.43)	1.35 (1.28)
	Injection site warmth	2699	3.79 (3.59 - 4.00)	3.71 (2638.70)	2.32 (2.22)	1.22 (1.15)
	Urticaria	2047	1.48 (1.40 - 1.56)	1.47 (218.07)	1.33 (1.27)	0.41 (0.34)
	Injection site pain	1890	2.36 (2.23 - 2.50)	2.34 (877.37)	1.80 (1.72)	0.85 (0.77)
	Injection site induration	1772	4.13 (3.86 - 4.43)	4.08 (1924.54)	2.43 (2.29)	1.28 (1.20)
	Feeling hot	1403	9.07 (8.22 - 10.01)	8.96 (2816.25)	3.25 (2.99)	1.70 (1.60)
	Injection site hypersensitivity	1376	2.42 (2.26 - 2.59)	2.40 (674.25)	1.83 (1.73)	0.87 (0.78)

DTaP, diphtheria, tetanus, and acellular pertussis; PT, preferred term; CI, confidence interval; ROR, reporting odds ratio; PRR, proportional reporting ratio; χ^2^, Chi-squared; IC, information component; IC-2SD, the lower limit of the 95% two-sided CI of the IC; EBGM, empirical Bayesian geometric mean; EBGM05, the lower 95 two-sided CI of EBGM.

The top 10 significant PTs for the DTaP vaccine were the same between females and males, differing only in the rankings of a few PTs (e.g., urticaria). Injection site reactions were the most frequent PTs for the DTaP vaccines in both males and females. Injection site erythema (females: a = 6,463, ROR = 3.63, PRR = 3.44, EBGM = 2.26, IC = 1.17; males: a = 7,042, ROR = 3.75, PRR = 3.57, EBGM = 2.28, IC = 1.19) and injection site swelling (females: a = 3,318, ROR = 2.87, PRR = 2.80, EBGM = 2.02, IC = 1.01; males: a = 3,883, ROR = 2.97, PRR = 2.90, EBGM = 2.04, IC = 1.03) were the most prevalent. Additionally, erythema (females: a = 2,811, ROR = 2.96, PRR = 2.90, EBGM = 2.06, IC = 1.04; males: a = 3,404, ROR = 2.96, PRR = 2.90, EBGM = 2.04, IC = 1.03), an adverse reaction in the skin and subcutaneous tissue disorders system organ class, also demonstrated high incidence. **Supplementary Figure S1** displays the association between signal strength (ROR) and statistical significance for SOC-level AEs associated with DTaP vaccination in females and males, respectively.

#### PTs across age groups

We conducted a stratified analysis by age group: < 2, ≥ 2 and < 5, ≥ 5 and < 7 years. **Table 4** presents the top ten significant PTs fulfilling signal detection criteria in at least one of four statistical methods across these three age groups.

**Table 4:** Signal strength of reports of DTaP vaccines at the PT level grouped by age.

Age	PT	a	ROR (95%Cl)	PRR (χ^2^)	EBGM (EBGM05)	IC (IC-2SD)
< 2	Injection site erythema	3693	3.25 (3.11 - 3.39)	3.17 (3361.09)	2.31 (2.23)	1.21 (1.15)
	Crying	2612	1.12 (1.07 - 1.17)	1.12 (25.79)	1.09 (1.05)	0.13 (0.07)
	Irritability	2075	1.64 (1.56 - 1.72)	1.63 (378.91)	1.47 (1.41)	0.55 (0.48)
	Injection site swelling	1918	3.04 (2.87 - 3.22)	3.01 (1597.15)	2.24 (2.13)	1.16 (1.08)
	Convulsion	1901	1.56 (1.48 - 1.65)	1.55 (287.04)	1.42 (1.36)	0.50 (0.43)
	Urticaria	1835	1.67 (1.59 - 1.77)	1.66 (364.78)	1.49 (1.43)	0.58 (0.50)
	Erythema	1622	2.04 (1.93 - 2.17)	2.03 (599.35)	1.72 (1.64)	0.78 (0.70)
	Agitation	1584	1.07 (1.01 - 1.13)	1.07 (5.70)	1.06 (1.01)	0.08 (0.00)
	Injection site oedema	1581	2.89 (2.72 - 3.08)	2.86 (1214.15)	2.17 (2.06)	1.12 (1.03)
	Screaming	1565	1.17 (1.11 - 1.24)	1.17 (31.24)	1.14 (1.08)	0.18 (0.10)
≥ 2 and < 5	Injection site erythema	6305	2.99 (2.88 - 3.10)	2.76 (3657.24)	1.85 (1.80)	0.89 (0.85)
	Injection site swelling	3483	2.21 (2.11 - 2.32)	2.14 (1200.78)	1.62 (1.56)	0.70 (0.64)
	Erythema	2796	2.93 (2.77 - 3.09)	2.83 (1626.40)	1.88 (1.79)	0.91 (0.84)
	Injection site warmth	2513	2.68 (2.54 - 2.84)	2.61 (1275.76)	1.80 (1.72)	0.85 (0.78)
	Injection site oedema	2350	5.62 (5.23 - 6.04)	5.43 (2797.95)	2.43 (2.29)	1.28 (1.21)
	Injection site pain	1754	1.90 (1.78 - 2.02)	1.87 (420.88)	1.51 (1.43)	0.59 (0.51)
	Injection site induration	1616	3.43 (3.18 - 3.69)	3.36 (1184.09)	2.03 (1.91)	1.02 (0.93)
	Feeling hot	1249	9.55 (8.48 - 10.76)	9.36 (2043.98)	2.82 (2.55)	1.49 (1.39)
	Pruritus	1141	1.86 (1.72 - 2.01)	1.84 (260.54)	1.49 (1.40)	0.58 (0.48)
	Swelling	1140	2.99 (2.74 - 3.25)	2.95 (694.78)	1.91 (1.78)	0.94 (0.83)
≥ 5 and < 7	Injection site erythema	3847	3.35 (3.18 - 3.52)	3.10 (2363.55)	1.86 (1.78)	0.89 (0.83)
	Erythema	1921	2.94 (2.74 - 3.15)	2.84 (1012.15)	1.79 (1.69)	0.84 (0.76)
	Injection site swelling	1893	2.22 (2.08 - 2.37)	2.16 (605.07)	1.58 (1.49)	0.66 (0.57)
	Injection site oedema	1730	5.31 (4.87 - 5.8)	5.11 (1721.19)	2.21 (2.05)	1.14 (1.05)
	Injection site warmth	1644	3.02 (2.8 - 3.25)	2.92 (900.69)	1.81 (1.70)	0.86 (0.77)
	Injection site pain	1168	2.01 (1.85 - 2.18)	1.98 (298.5)	1.51 (1.41)	0.59 (0.49)
	Pruritus	1050	2.04 (1.87 - 2.22)	2.01 (280.35)	1.52 (1.42)	0.60 (0.50)
	Injection site induration	945	4.21 (3.77 - 4.70)	4.12 (772.52)	2.07 (1.88)	1.05 (0.93)
	Feeling hot	923	9.98 (8.57 - 11.63)	9.75 (1314.79)	2.57 (2.26)	1.36 (1.23)
	Injection site hypersensitivity	850	2.36 (2.14 - 2.61)	2.33 (313.48)	1.64 (1.51)	0.71 (0.59)

DTaP, diphtheria, tetanus, and acellular pertussis; PT, preferred term; CI, confidence interval; ROR, reporting odds ratio; PRR, proportional reporting ratio; χ^2^, Chi-squared; IC, information component; IC-2SD, the lower limit of the 95% two-sided CI of the IC; EBGM, empirical Bayesian geometric mean; EBGM05, the lower 95 two-sided CI of EBGM.

In the age group < 2 years, the three most frequently reported significant PTs were injection site erythema (a = 3,693, ROR = 3.25, PRR = 3.17, EBGM = 2.31, IC = 1.21), crying (a = 2,612, ROR = 1.12, PRR = 1.12, EBGM = 1.09, IC = 0.13), and irritability (a = 2,075, ROR = 1.64, PRR = 1.63, EBGM = 1.47, IC = 0.55). In the age group ≥ 2 and < 5 years, the three most frequently reported significant PTs were injection site erythema (a = 6,305, ROR = 2.99, PRR = 2.76, EBGM = 1.85, IC = 0.89), injection site swelling (a = 3,483, ROR = 2.21, PRR = 2.14, EBGM = 1.62, IC = 0.70), erythema (a = 2,796, ROR = 2.93, PRR = 2.83, EBGM = 1.88, IC = 0.91). In the age group ≥ 5 and < 7 years, the three most frequently reported significant PTs were injection site erythema (a = 3,847, ROR = 3.35, PRR = 3.10, EBGM = 1.86, IC = 0.89), erythema (a = 1,912, ROR = 2.94, PRR = 2.84, EBGM = 1.79, IC = 0.84), injection site swelling (a = 1,893, ROR = 2.22, PRR = 2.16, EBGM = 1.58, IC = 0.66). **Supplementary Figure S2** displays the association between signal strength (ROR) and statistical significance for SOC-level AEs associated with DTaP vaccination in < 2, ≥ 2 and < 5, ≥ 5 and < 7 years, respectively.

### SAEs and fatalities

**Figure 4:**
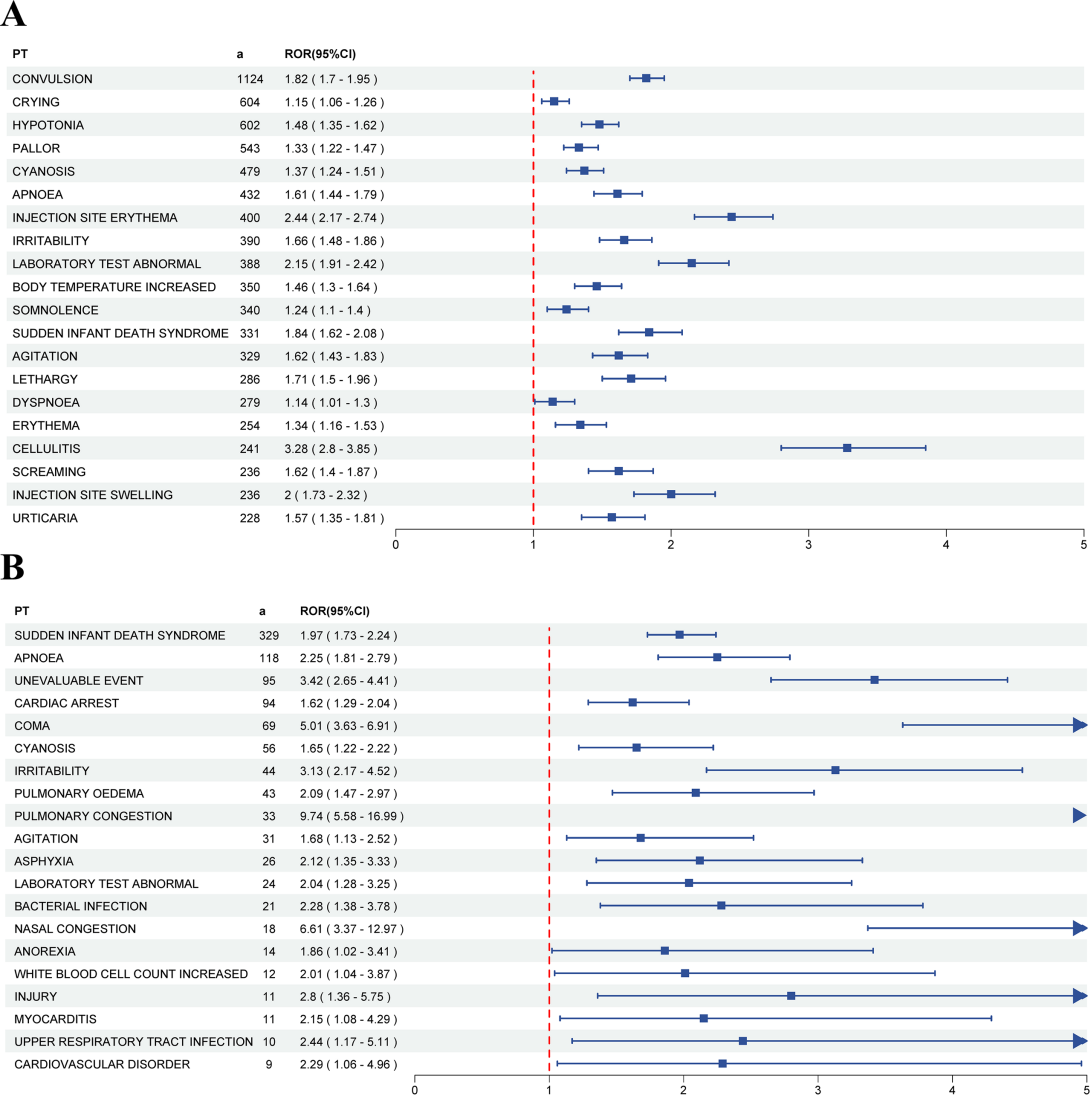
The forest plot of top twenty DTaP vaccine-related positive PT signals. **(A)** SAEs, **(B)** Fatalities. DTaP, diphtheria, tetanus, and acellular pertussis; SAE, serious adverse event; PT, preferred term; ROR, reporting odds ratio.

Within VAERS, SAEs were reported in 13.30% (7,625/57,341) of children < 7 years following DTaP vaccination. Among all SAEs, pyrexia (a = 2,326, ROR = 1.01, PRR = 1.01, EBGM = 1.01, IC = 0.02) was the most frequently reported, followed by convulsion (a = 1,124, ROR = 1.82, PRR = 1.80, EBGM = 1.61, IC = 0.68) and vomiting (a = 928, ROR = 1.05, PRR = 1.05, EBGM = 1.04, IC = 0.06). **Figure 4A** presents the top twenty positive SAE PT signals related to DTaP vaccines, while **Supplementary Table S5** lists all identified positive SAE PT signals. At the SOC level, the majority of SAEs occurred in the investigations system organ class (a = 8,321, ROR = 0.58, PRR = 0.66, EBGM = 0.70, IC = -0.52), followed by general disorders and administration site conditions (a = 7,875, ROR = 1.25, PRR = 1.21, EBGM = 1.17, IC = 0.23), and nervous system disorders (a = 6,767, ROR = 1.37, PRR = 1.31, EBGM = 1.25, IC = 0.33). **Supplementary Figure S3A** displays the association between signal strength (ROR) and statistical significance for SOC-level SAEs associated with DTaP vaccination. **Supplementary Table S6** presents the signal strengths of DTaP vaccine-related SAEs at the SOC level.

Among children younger than 7 years in VAERS, the proportion of reports that were fatal after DTaP vaccination was 1.49% (854/57,341). At the PT level, the most frequently reported fatal events were sudden infant death syndrome (SIDS, a = 329, ROR = 1.97, PRR = 1.87, EBGM = 1.66, IC = 0.73), followed by death (a = 169, ROR = 0.53, PRR = 0.56, EBGM = 0.59, IC = -0.75) and apnoea (a = 118, ROR = 2.25, PRR = 2.20, EBGM = 1.87, IC = 0.91). **Figure 4B** presents the top twenty positive SAE PT signals related to DTaP vaccines, while **Supplementary Table S7** lists all identified positive SAE PT signals. At the SOC level, the majority of fatalities occurred in the general disorders and administration site conditions (a = 872, ROR = 1.12, PRR = 1.09, EBGM = 1.07, IC = 0.10), followed by respiratory, thoracic and mediastinal disorders (a = 455, ROR = 1.46, PRR = 1.39, EBGM = 1.32, IC = 0.40), and investigations system organ class (a = 360, ROR = 0.53, PRR = 0.58, EBGM = 0.62, IC = -0.69). **Supplementary Figure S3B** displays the association between signal strength (ROR) and statistical significance for SOC-level fatal SAEs associated with DTaP vaccination. **Supplementary Table S8** presents the signal strengths of DTaP vaccine-related fatal SAEs at the SOC level.

### Sudden infant death syndrome

As previously described, SIDS was identified as a positive safety signal for fatalities following DTaP vaccination, and we conducted an in-depth analysis of 329 reported SIDS cases. As presented in **Table 5**, among the 329 cases with available data, a total of 223 cases (67.78%) were observed within the first 6 days (0–6 days) post-vaccination, with a mean interval of 2.18 days. The frequency of cases declined markedly thereafter: 31 cases (9.42%) occurred between days 7 and 13 (mean interval: 9.77 days), followed by 12 cases (3.65%) in days 14-20, and 14 cases (4.26%) in days 21-28. Only 23 cases (6.99%) were documented at 29 days or later. Notably, the time interval data were not available for 26 cases (7.90%). Notably, an adverse event was reported in only one child prior to SIDS, and it was a serious adverse event. The event, cardiac arrest occurring after vaccination, falls under the System Organ Class of Cardiac disorders.

**Table 5:** Distribution of SIDS cases and mean time to death by interval since DTaP vaccination.

Time since vaccination (Days)	Number of SIDS cases	Mean interval (Days)
0-6	223 (67.78%)	2.18
7-13	31 (9.42%)	9.77
14-20	12 (3.65%)	16.67
21-28	14 (4.26%)	22.64
≥29	23 (6.99%)	85.70
NA	26 (7.90%)	–

SIDS, Sudden infant death syndrome; DTaP: diphtheria, tetanus, and acellular pertussis; NA, not available.

### Designated medical event list screening

Established by the European Medicines Agency, the Designated Medical Event registry lists suspected AEs designated for prioritized pharmacovigilance monitoring. Of the total detected signals, 37 Designated Medical Event-associated PT signals spanning 12 SOCs were identified. At the PT level, the most frequently reported AEs were erythema multiforme (a = 280, ROR = 1.28, PRR = 1.28, EBGM = 1.21, IC = 0.27), anaphylactic reaction (a = 133, ROR = 0.85, PRR = 0.85, EBGM = 0.87, IC = -0.19), and angioedema (a = 52, ROR = 0.70, PRR = 0.70, EBGM = 0.74, IC = -0.43). Positive signals were detected for only two Designated Medical Event-associated PTs: erythema multiforme (a = 280, ROR = 1.28, PRR = 1.28, EBGM = 1.21, IC = 0.27) and aplasia pure red cell (a = 3, ROR = 5.54, PRR = 5.54, EBGM = 2.82, IC = 1.49) (**Supplementary Table S9**).

## Discussion

This investigation represents the first pharmacovigilance study to consolidate safety signals across multiple DTaP vaccines based on the U.S. VAERS database. The safety profiles of post-licensure DTaP vaccines were evaluated through disproportionality analyses and monitoring of the Designated Medical Event list within VAERS. When interpreting the signals identified in this study, it is important to reiterate the exploratory nature of disproportionality analyses. While a corrected p-value < 0.05 served as our primary threshold for highlighting associations, we intentionally examined findings with p-values between 0.05 and 0.06. These results, though not meeting conventional statistical significance, may represent potential signals—especially for events with strong prior biological plausibility. Therefore, we recommend that subsequent studies pay adequate attention to these findings to prevent such potential signals from being overlooked.

According to a review of the VAERS database, SAEs were reported in 7,625 instances, accounting for 13.3% of all reports; while non-serious cases totaled 49,716, comprising 86.7% of the total. Serious adverse events were reported in 7,625 instances, accounting for 13.3% of the total. Of these, only 11 (0.01%) vaccinees experienced two or more serious adverse events, while 7,614 (99.9%) reported a single occurrence. Among all positive signals for SAEs, convulsions were the most frequently reported, with 1,124 cases. Febrile seizure represented the most common type of convulsion in children (Grünewald et al., 2001). Such seizures may be associated with febrile infections and have also been linked to immunization with Diphtheria, Tetanus, whole-cell Pertussis (DTwP), 13-valent pneumococcal conjugate (PCV-13), and trivalent inactivated influenza vaccines (TIV) (Duffy et al., 2017). There were 854 fatalities among the reported AEs, accounting for approximately 1.49% of all AEs and 11.2% of all SAEs. Based on our findings, SIDS was the most frequently reported diagnosis among DTaP-associated fatal cases and showed a statistical signal, although this likely reflects temporal association rather than causation. However, an assessment of the risk of sudden unexplained infant death (SUID) following the introduction of DTaP in 2010 did not find evidence of an increased risk in Taiwan (Huang et al., 2017). The absence of a causal association between vaccination and SIDS is demonstrated by a considerable body of evidence (Brotherton et al., 2005; Vennemann et al., 2007). Notably, a 2003 review by the Institute of Medicine (IOM) of the U.S. concluded that neither the whole cell pertussis-containing vaccine (now discontinued in the United States) nor the administration of multiple simultaneous vaccines was causally linked to SIDS (Institute of Medicine (US) Immunization Safety Review Committee, 2003).

However, according to our study results, the temporal distribution of SIDS cases following DTaP vaccination revealed a pronounced and clinically significant pattern, with the overwhelming majority (67.78%) of cases clustered within the first week (06 days) post-immunization. This highly skewed distribution, where the incidence rate in the initial period is markedly higher than in subsequent weeks, suggests a non-random temporal association. The finding that over two-thirds of cases occur within this narrow window strengthens the signal of a potential temporal link, as it is inconsistent with a uniform or random distribution over time, which would be expected if no association existed. While this clustering alone does not establish causation, it provides compelling epidemiological evidence that merits serious biological and mechanistic investigation. It is plausible that the acute physiological stress or inflammatory response triggered by vaccination could interact with underlying vulnerabilities in a small subset of infants, potentially tipping the balance during a critical developmental period. This observation underscores the importance of meticulous post-vaccination monitoring, particularly in the first week, and highlights an urgent need for further research aimed at identifying potential sub-populations at risk and elucidating the precise pathophysiological pathways that might connect the vaccine’s biological effects to this tragic outcome.

According to disproportionality analysis, injection site erythema, pyrexia, injection site swelling, erythema, and injection site oedema were the most commonly reported AEs following DTaP vaccination. The vast majority of signals identified were known and expected local or mild reactions. Most reported adverse reactions were self-limited, involving mild local and systemic events. This finding is well-aligned with both the anticipated safety profile and the descriptions contained within the vaccine’s prescribing information (Moro et al., 2018; Ujiie et al., 2021). Virtually all DTaP vaccines are delivered by intramuscular injection, a practice that contributes to the prevalence of injection site-related AEs among reported vaccine reactions. Studies have identified several elements influencing a vaccine’s safety and immunogenicity profile (Herzog, 2014). These factors include the anatomical site of injection, needle length, administration technique, injection depth, antigen characteristics, vaccine formulation, inclusion of adjuvants, as well as patient-specific variables such as age, sex, race/ethnicity, body mass, and pre-existing immune status (Cook et al., 2006; Herzog, 2014).

We conducted subgroup analyses to evaluate the safety of DTaP vaccination in children under 7 years of age, stratified by sex and age. In the sex-based subgroups, the top ten types of AEs were nearly identical between females and males, with the only difference observed in the frequency ranking of the PT. This indicates that there are no significant differences in the safety profile of DTaP between different genders. For the age-based subgroup analysis, participants were categorized into three groups: <2 years, ≥2 to <5 years, and ≥5 to <7 years. Among these, children under 2 years old experienced the highest number of injection site-related AEs within their own group; however, this number was relatively lower compared to the other two age groups. In contrast, AEs such as crying, irritability, and convulsion were reported more frequently in the <2 years subgroup, which can be interpreted as normal behavioral responses to discomfort or fever in infants and young children, thereby providing clinical reassurance. This behavior likely represents a primary means for infants and young children to express distress, which is fully consistent with the physiological and psychological characteristics of this developmental stage (Tse et al., 2012; Liu et al., 2022). For both the ≥2 to <5 years and ≥5 to <7 years groups, injection site-related AEs accounted for most reported AEs.

This study found that the vast majority of AE reports, specifically 89.61%, were clustered in the 0- to 30-day window after DTaP vaccination. It is crucial to emphasize that attributing these events to the vaccination requires a thorough causality assessment based on a structured framework. This involves the strict application of established methods, notably the WHO guidelines for Causality Assessment of AEFI (World Health Organization, 2021). These guidelines recommend a consolidated evaluation of several criteria, such as the temporal relationship, biological plausibility, responses to dechallenge or rechallenge if applicable, and the exploration of alternative etiologies (World Health Organization, 2021). Hence, the strong temporal association observed, while being a prerequisite for further causal evaluation, is not independently sufficient. The observed clustering could potentially be explained by reporting biases or the coincidental occurrence of unrelated illnesses, and thus it does not by itself confirm a causal link.

Among the Designated Medical Events screened, two conditions, erythema multiforme and pure red cell aplasia, met the initial signal criteria. Our analysis identified 280 reports with a mild disproportionality signal for erythema multiforme, a finding consistent with the known, albeit very low, background risk of cutaneous hypersensitivity events following immunizations (Masseria et al., 2015; Moro et al., 2018; Loiacono et al., 2021; Ujiie et al., 2021). In contrast, pure red cell aplasia is an exceedingly rare hematologic disorder, with only 3 cases reported here. The pure red cell aplasia signal should therefore be interpreted with utmost caution; it may represent a chance finding, unrelated coincidental illnesses, or extreme statistical variation rather than a causal association with DTaP vaccination. Crucially, for both erythema multiforme and pure red cell aplasia, the identified signals highlight conditions that warrant clinical vigilance. Their identification underscores the value of targeted Designated Medical Event screening in pharmacovigilance to flag potential but rare serious risks for further professional evaluation and ongoing monitoring.

Our analysis of DTaP vaccines in VAERS reports (1990-2025) reveals distinct temporal trends in both vaccine usage and AE reporting volumes. Infanrix^®^ (37.95%), Daptacel^®^ (25.04%), and Tripedia^®^ (24.13%) accounted for the largest proportions of reported adverse events, which may be attributed to their long-standing recommendation for children under 7 years of age. In comparison, other formulations including Acel-Imune^®^ (5.47%) and Certiva^®^ (3.10%) were less frequently reported, likely due to their discontinued promotion or market withdrawal in the United States. Data on manufacturers indicated a predominance of Sanofi Pasteur (38.46%) and GlaxoSmithKline Biologicals (25.48%), consistent with their status as key DTaP vaccine producers. These results underscore the importance of ongoing surveillance for newer vaccines as their use expands, along with the need for more complete documentation of vaccination details to strengthen the accuracy of post-marketing safety monitoring (Ellis-Legault et al., 2025).

Our study found that among most children under seven years old who received the DTaP vaccine, the number of reported adverse reactions increased after the fourth or fifth dose. This is a concerning and noteworthy finding. Several factors may explain this observation. It may be due to a cumulative immune response, namely that later booster doses could trigger a stronger anamnestic response, leading to more pronounced inflammatory reactions such as high fever or generalized muscle pain. In addition, the older age of children at the time of later doses and the longer intervals between vaccinations might alter how their immune system reacts compared to infancy. Reporting bias could also play a role, as parents or healthcare providers may be more attentive and thus more likely to report symptoms in children completing their vaccination series. Finally, a genuine cumulative biological effect from vaccine components, such as adjuvants or antigens, cannot be ruled out.

Our findings provide direct and actionable guidance for post-marketing DTaP vaccine safety management. Primarily, they serve to clinically reassure healthcare providers that the most frequently detected signals align with well-established, transient local reactogenicity. This evidence-based confirmation aids in effective communication with parents, helping to manage expectations and alleviate concerns regarding common post-vaccination symptoms. For pharmacovigilance systems, our analysis offers a strategic framework for resource prioritization by clearly differentiating between high-volume, expected adverse events and the subset of rare but serious signals, such as certain designated medical events, which merit targeted investigation and follow-up. Furthermore, we underscore a key public health recommendation: authorities should maintain structured vigilance for these flagged rare events. This proactive stance ensures preparedness to initiate focused epidemiological studies should reporting patterns or volumes change significantly, thereby enabling a science-based response. Collectively, this work strengthens the ongoing real-world safety evidence base, supporting the continued confident use of DTaP vaccines.

VAERS offers two principal advantages: comprehensive national scope and the ability to accept reports in near real-time. As an initial signal detection tool, the system plays an essential role in recognizing rare AEs that are unusual or unanticipated, potentially prompting further inquiry via active surveillance systems or formal epidemiological research. Nevertheless, due to its fundamental reliance on voluntary, spontaneous submissions, VAERS is intrinsically constrained by limited data detail and an inability to definitively establish causation, requiring that any identified signals be interpreted with due caution. A core limitation of the VAERS system is the absence of denominator data, specifically the total number of administered vaccine doses. Without these exposure figures, it is impossible to compute precise incidence rates or risks for reported adverse events. Therefore, any observed distribution patterns only represent proportions among submitted reports and cannot be used to infer actual occurrence rates or risk levels in the general vaccinated population. Another significant constraint involves the frequent lack of essential clinical and laboratory details in the medical records of serious cases, even upon follow-up. Collectively, these limitations substantially weaken the capacity to establish causal links between vaccination and reported AEs (McNeil et al., 2014; Moro et al., 2019). In addition, due to the data structure of VAERS, it is not possible to fully distinguish whether an adverse event is caused solely by DTaP, by other vaccines administered concurrently, or by a combination of these. Despite these shortcomings, VAERS retains important value as an initial screening tool for uncommon or unexpected safety signals, though any such signals require subsequent confirmation through rigorously designed epidemiological studies. Future studies should include extended follow-up durations, systematic evaluation of treatment outcomes, and integrated pharmacokinetic analyses to better support causal inferences. Even given these inherent system constraints, our findings provide a foundation for future research and can inform more evidence-based immunization strategies.

## Conclusions

A disproportionality analysis of VAERS data identified a distinct cluster of 158 AEs significantly associated with DTaP vaccination in children under seven. The vaccine’s reactogenicity profile is primarily characterized by local reactions, most notably injection site erythema, injection site swelling, erythema, injection site oedema, and injection site warmth, which predominantly arise within the first 30 days. These evidence-based findings are crucial for guiding enhanced safety surveillance and for shaping risk-benefit evaluations and mitigation strategies among healthcare authorities and providers.

## Data Availability

Publicly available datasets were analyzed in this study. This data can be found here: The data utilized in these analyses are publicly accessible through the VAERS dataset, available at https://vaers.hhs.gov/index.html (accessed on 20 June 2025).
