# Supplementary material for: Diphtheria-tetanus-acellular pertussis vaccine safety in children under 7 years: a post-marketing analysis of the U.S. vaccine adverse event reporting system
Source: Front Cell Infect Microbiol. 2026 Feb 2;16:1733777. doi: 10.3389/fcimb.2026.1733777 (PMC12907357; doi:10.3389/fcimb.2026.1733777)
Supplement: Supplementary file 1 [file Table1.docx]

**Contents**

[Table S1. Two-by-two contingency table for measure of disproportionality 2](#_Toc202908711)

[Table S2. Measure of disproportionality and signal generation criteria 3](#_Toc202908712)

[Table S3. Signal strength of all DTaP vaccine-related positive PT signals and their corresponding SOCs 4](#_Toc202908713)

[Table S4. Signal strength of AE reports of DTaP vaccines at the SOC level. 18](#_Toc202908714)

[Figure S1. Volcano plot of SOCs corresponding to reported AEs in females and males. 20](#_Toc202908715)

[Figure S2. Volcano plot of SOCs corresponding to reported AEs in < 2, ≥ 2 and < 5, ≥ 5 and < 7 years. 21](#_Toc202908716)

[Table S5. Signal strength of DTaP vaccine-related positive SAE PT signals and their corresponding SOCs 22](#_Toc202908717)

[Figure S3. Volcano plot of SOCs corresponding to reported SAEs and fatalities. 43](#_Toc202908718)

[Table S6. Signal strength of SAE reports of DTaP vaccines at the SOC level. 44](#_Toc202908719)

[Table S7. Signal strength of DTaP-related fatal SAE positive PT signals and their corresponding SOCs. 46](#_Toc202908720)

[Table S8. Signal strength of fatal reports of DTaP vaccines at the SOC level. 51](#_Toc202908721)

[Table S9. Signal strength of all DTaP-related DME-associated PT signals and their corresponding SOCs. 53](#_Toc202908722)

# Table S1. Two-by-two contingency table for measure of disproportionality

|  | No. target adverse reaction reports | No. other adverse reaction reports | Total |
| --- | --- | --- | --- |
| Target drug | a | b | a + b |
| Other drugs | c | d | c + d |
| Total | a + c | b + d | N = a + b + c + d |

a, number of reports containing both the suspect drug and the suspect adverse drug reaction; b, number of reports containing the suspect adverse drug reaction with other medications (except the drug of interest); c, number of reports containing the suspect drug with other adverse drug reactions (except the event of interest); d, number of reports containing other medications and other adverse drug reactions.

# Table S2. Measure of disproportionality and signal generation criteria

| Method | Formula | Threshold |
| --- | --- | --- |
| ROR | $ROR=\frac{a/c}{b/d}=\frac{ad}{bc}$  $SE(lnROR)=\sqrt{(\frac{1}{a}+\frac{1}{b}+\frac{1}{c}+\frac{1}{d})}$  $95\% CI=e^{ln\left( ROR \right)\pm1.96\sqrt{(\frac{1}{a}+\frac{1}{b}+\frac{1}{c}+\frac{1}{d})}}$ | a ≥ 3 and 95% CI (lower limit) > 1 |
| PRR | $PRR=\frac{a/{(a+b)}}{c/{(c+d)}}$  $95\% CI=e^{ln(PRR)\pm1.96\sqrt{(\frac{1}{a}-\frac{1}{a+b}+\frac{1}{c}-\frac{1}{c+d})}}$  ${}^{2}=\frac{{N(\left\vert ad-bc \right\vert-\frac{N}{2})}^{2}}{(a+b)(c+d)(b+d)(a+c)}$ | a ≥ 3 and PRR ≥ 2 and χ^2^ ≥ 4 |
| BCPNN | $IC={log}_{2}^{\frac{p(x,y)}{p\left( x \right)p(y)}}={log}_{2}^{\frac{a(a+b+c+d)}{(a+b)(a+c)}}$  $E\left( IC \right)={log}_{2}^{\frac{(a+\gamma ij)(a+b+c+d+\alpha)(a+b+c+d+\beta)}{(a+b+c+d+\gamma)(a+b+\alpha i)(a+b+\beta j)}}$  $V(IC)$=$\frac{1}{{(ln2)}^{2}}\left\{ \left[ \frac{\left( a+b+c+d \right)-a+\gamma-\gamma ij}{(a+\gamma ij)(1+a+b+c+d+\gamma)} \right]+\left[ \frac{\left( a+b+c+d \right)-(a+b)+\alpha-\alpha i}{(a+b+\alpha i)(1+a+b+c+d+\alpha)} \right]+\left[ \frac{\left( a+b+c+d \right)-(a+c)+\beta-\beta j}{(a+b+\beta j)(1+a+b+c+d+\beta)} \right] \right\}$  $\gamma=\gamma ij\frac{(a+b+c+d+\alpha)(a+b+c+d+\beta)}{(a+b+\alpha i)(a+c+\beta j)}$  *IC-2SD*$=E\left( IC \right)-2\sqrt{V(IC)}$ | a ≥ 3 and IC-2SD > 0 |
| MGPS | $EBGM=\frac{a(a+b+c+d)}{(a+c)(a+b)}$  $EBGM05=e^{\ln\left( EBGM \right)-1.64\sqrt{(\frac{1}{a}+\frac{1}{b}+\frac{1}{c}+\frac{1}{d})}}$ | a > 0 and EBGM05 > 2 |

ROR, reporting odds ratio; CI, confidence interval; PRR, proportional reporting ratio; BCPNN, Bayesian confidence propagation neural network; IC, information components; MGPS, multi-item gamma Poisson shrinker; EBGM, empirical Bayes geometric mean; EBGM05, the lower 95% one-side CI of EBGM; IC-2SD, the lower limit of the 95% two-sided CI of the IC; E is the expected number of cases.

# Table S3. Signal strength of all DTaP vaccine-related positive PT signals and their corresponding SOCs

| SOC | PT | a | ROR (95%Cl) | PRR (χ^2^) | EBGM (EBGM05) | IC (IC-2SD) | *p*-adjust |
| --- | --- | --- | --- | --- | --- | --- | --- |
| General disorders and administration site conditions | Injection site erythema | 13845 | 3.79  (3.70 - 3.88) | 3.59  (13550.12) | 2.31  (2.27) | 1.21  (1.18) | < 0.001 |
| General disorders and administration site conditions | Injection site swelling | 7294 | 3.00  (2.91 - 3.10) | 2.93  (5261.68) | 2.08  (2.02) | 1.05  (1.01) | < 0.001 |
| Skin and subcutaneous tissue disorders | Erythema | 6339 | 3.03  (2.93 - 3.14) | 2.97  (4659.80) | 2.09  (2.03) | 1.06  (1.02) | < 0.001 |
| General disorders and administration site conditions | Injection site oedema | 5661 | 4.74  (4.55 - 4.93) | 4.63  (7226.43) | 2.61  (2.53) | 1.38  (1.34) | < 0.001 |
| General disorders and administration site conditions | Injection site warmth | 5273 | 3.97  (3.82 - 4.13) | 3.89  (5576.29) | 2.41  (2.33) | 1.27  (1.22) | < 0.001 |
| General disorders and administration site conditions | Injection site pain | 3778 | 2.47  (2.37 - 2.58) | 2.44  (1961.02) | 1.87  (1.81) | 0.90  (0.85) | < 0.001 |
| Skin and subcutaneous tissue disorders | Urticaria | 3656 | 1.46  (1.40 - 1.52) | 1.45  (373.34) | 1.32  (1.28) | 0.40  (0.35) | < 0.001 |
| General disorders and administration site conditions | Injection site induration | 3613 | 4.14  (3.95 - 4.34) | 4.08  (4026.36) | 2.46  (2.37) | 1.30  (1.24) | < 0.001 |
| General disorders and administration site conditions | Feeling hot | 2733 | 9.73  (9.06 - 10.45) | 9.61  (5876.72) | 3.39  (3.19) | 1.76  (1.69) | < 0.001 |
| General disorders and administration site conditions | Injection site hypersensitivity | 2670 | 2.35  (2.24 - 2.47) | 2.33  (1256.19) | 1.82  (1.74) | 0.86  (0.80) | < 0.001 |
| Skin and subcutaneous tissue disorders | Pruritus | 2606 | 2.28  (2.17 - 2.39) | 2.26  (1142.88) | 1.78  (1.71) | 0.83  (0.77) | < 0.001 |
| General disorders and administration site conditions | Swelling | 2403 | 3.71  (3.51 - 3.93) | 3.68  (2360.81) | 2.34  (2.23) | 1.23  (1.16) | < 0.001 |
| Psychiatric disorders | Irritability | 2324 | 1.25  (1.19 - 1.31) | 1.24  (83.82) | 1.18  (1.14) | 0.24  (0.17) | < 0.001 |
| Nervous system disorders | Convulsion | 2300 | 1.20  (1.14 - 1.26) | 1.20  (56.27) | 1.15  (1.10) | 0.20  (0.13) | < 0.001 |
| Infections and infestations | Cellulitis | 2088 | 4.51  (4.23 - 4.81) | 4.47  (2556.32) | 2.57  (2.44) | 1.36  (1.28) | < 0.001 |
| General disorders and administration site conditions | Injection site reaction | 2044 | 2.52  (2.38 - 2.67) | 2.50  (1106.15) | 1.90  (1.81) | 0.92  (0.85) | < 0.001 |
| General disorders and administration site conditions | Injection site mass | 1893 | 2.72  (2.56 - 2.89) | 2.7  (1179.44) | 1.98  (1.89) | 0.99  (0.91) | < 0.001 |
| General disorders and administration site conditions | Oedema peripheral | 1789 | 3.45  (3.23 - 3.68) | 3.42  (1599.78) | 2.26  (2.14) | 1.18  (1.09) | < 0.001 |
| General disorders and administration site conditions | Pain | 1724 | 1.65  (1.56 - 1.75) | 1.65  (305.7) | 1.45  (1.38) | 0.53  (0.45) | < 0.001 |
| General disorders and administration site conditions | Oedema | 1616 | 4.22  (3.93 - 4.53) | 4.19  (1844.57) | 2.49  (2.35) | 1.32  (1.23) | < 0.001 |
| Vascular disorders | Vasodilatation | 1115 | 1.65  (1.54 - 1.77) | 1.65  (196.41) | 1.45  (1.36) | 0.53  (0.43) | < 0.001 |
| General disorders and administration site conditions | Tenderness | 995 | 5.30  (4.81 - 5.83) | 5.27  (1421.99) | 2.76  (2.55) | 1.46  (1.35) | < 0.001 |
| Skin and subcutaneous tissue disorders | Skin warm | 989 | 1.85  (1.71 - 1.99) | 1.84  (254.56) | 1.56  (1.46) | 0.64  (0.54) | < 0.001 |
| Nervous system disorders | Lethargy | 944 | 1.14  (1.06 - 1.23) | 1.14  (12.26) | 1.11  (1.04) | 0.15  (0.04) | 0.001 |
| General disorders and administration site conditions | Injection site rash | 857 | 1.38  (1.27 - 1.49) | 1.37  (63.98) | 1.27  (1.19) | 0.35  (0.24) | < 0.001 |
| Immune system disorders | Hypersensitivity | 836 | 1.65  (1.52 - 1.79) | 1.65  (148.58) | 1.45  (1.35) | 0.54  (0.42) | < 0.001 |
| Skin and subcutaneous tissue disorders | Rash macular | 673 | 1.45  (1.32 - 1.58) | 1.45  (66.75) | 1.32  (1.23) | 0.40  (0.28) | < 0.001 |
| General disorders and administration site conditions | Injection site pruritus | 632 | 1.67  (1.52 - 1.83) | 1.67  (116.62) | 1.46  (1.35) | 0.55  (0.41) | < 0.001 |
| Investigations | Laboratory test abnormal | 597 | 1.32  (1.2 - 1.45) | 1.32  (33.87) | 1.23  (1.14) | 0.30  (0.17) | < 0.001 |
| General disorders and administration site conditions | Injection site inflammation | 568 | 3.53  (3.15 - 3.96) | 3.53  (526.61) | 2.29  (2.08) | 1.20  (1.05) | < 0.001 |
| Nervous system disorders | Dyskinesia | 491 | 1.42  (1.28 - 1.58) | 1.42  (44.38) | 1.3  (1.20) | 0.38  (0.24) | < 0.001 |
| General disorders and administration site conditions | Local reaction | 490 | 1.65  (1.48 - 1.83) | 1.65  (86.28) | 1.45  (1.32) | 0.53  (0.38) | < 0.001 |
| Psychiatric disorders | Staring | 468 | 1.56  (1.40 - 1.74) | 1.56  (66.82) | 1.40  (1.27) | 0.48  (0.33) | < 0.001 |
| Skin and subcutaneous tissue disorders | Skin discolouration | 437 | 1.36  (1.22 - 1.51) | 1.36  (29.84) | 1.26  (1.15) | 0.33  (0.18) | < 0.001 |
| General disorders and administration site conditions | Induration | 419 | 1.81  (1.61 - 2.03) | 1.80  (101.00) | 1.54  (1.40) | 0.62  (0.46) | < 0.001 |
| General disorders and administration site conditions | Face oedema | 407 | 1.49  (1.33 - 1.67) | 1.49  (46.59) | 1.35  (1.22) | 0.43  (0.27) | < 0.001 |
| Nervous system disorders | Coma | 405 | 3.34  (2.92 - 3.82) | 3.33  (347.84) | 2.23  (1.99) | 1.15  (0.98) | < 0.001 |
| Musculoskeletal and connective tissue disorders | Musculoskeletal stiffness | 402 | 1.42  (1.27 - 1.60) | 1.42  (36.27) | 1.30  (1.18) | 0.38  (0.22) | < 0.001 |
| Respiratory, thoracic and mediastinal disorders | Wheezing | 396 | 1.19  (1.06 - 1.33) | 1.19  (9.04) | 1.14  (1.04) | 0.19  (0.03) | 0.005 |
| General disorders and administration site conditions | Injection site vesicles | 362 | 1.93  (1.70 - 2.19) | 1.93  (106.61) | 1.61  (1.45) | 0.69  (0.51) | < 0.001 |
| General disorders and administration site conditions | Injection site urticaria | 358 | 1.26  (1.12 - 1.42) | 1.26  (14.41) | 1.19  (1.08) | 0.26  (0.09) | < 0.001 |
| Eye disorders | Eye movement disorder | 354 | 1.33  (1.18 - 1.51) | 1.33  (21.82) | 1.25  (1.13) | 0.32  (0.14) | < 0.001 |
| Injury, poisoning and procedural complications | Contusion | 349 | 2.10  (1.84 - 2.40) | 2.10  (128.29) | 1.70  (1.52) | 0.77  (0.59) | < 0.001 |
| General disorders and administration site conditions | Sudden infant death syndrome | 331 | 1.19  (1.05 - 1.34) | 1.19  (7.31) | 1.14  (1.03) | 0.19  (0.01) | 0.012 |
| Infections and infestations | Injection site cellulitis | 317 | 1.54  (1.35 - 1.75) | 1.53  (41.77) | 1.38  (1.23) | 0.46  (0.28) | < 0.001 |
| Skin and subcutaneous tissue disorders | Erythema multiforme | 280 | 1.28  (1.12 - 1.47) | 1.28  (12.88) | 1.21  (1.08) | 0.27  (0.08) | 0.001 |
| General disorders and administration site conditions | Injection site nodule | 244 | 1.51  (1.30 - 1.75) | 1.51  (29.84) | 1.36  (1.20) | 0.45  (0.24) | < 0.001 |
| Eye disorders | Eye swelling | 224 | 1.33  (1.15 - 1.56) | 1.33  (13.81) | 1.25  (1.10) | 0.32  (0.10) | < 0.001 |
| Nervous system disorders | Depressed level of consciousness | 218 | 1.25  (1.07 - 1.45) | 1.25  (7.96) | 1.18  (1.04) | 0.24  (0.03) | 0.009 |
| Infections and infestations | Bacterial infection | 217 | 1.48  (1.26 - 1.73) | 1.48  (23.84) | 1.34  (1.17) | 0.42  (0.20) | < 0.001 |
| Skin and subcutaneous tissue disorders | Skin nodule | 215 | 2.65  (2.22 - 3.16) | 2.65  (128.45) | 1.96  (1.69) | 0.97  (0.74) | < 0.001 |
| Psychiatric disorders | Listless | 193 | 1.50  (1.27 - 1.77) | 1.50  (22.73) | 1.35  (1.18) | 0.44  (0.20) | < 0.001 |
| Skin and subcutaneous tissue disorders | Ecchymosis | 189 | 1.28  (1.09 - 1.51) | 1.28  (8.78) | 1.21  (1.05) | 0.28  (0.04) | 0.006 |
| Respiratory, thoracic and mediastinal disorders | Hypoxia | 188 | 1.65  (1.39 - 1.96) | 1.65  (33.56) | 1.45  (1.26) | 0.54  (0.30) | < 0.001 |
| Surgical and medical procedures | Vasodilation procedure | 184 | 18.90  (13.22 - 27.01) | 18.88  (509.96) | 3.93  (2.91) | 1.97  (1.69) | < 0.001 |
| General disorders and administration site conditions | Inflammation | 182 | 1.52  (1.28 - 1.81) | 1.52  (23.02) | 1.37  (1.18) | 0.45  (0.21) | < 0.001 |
| Metabolism and nutrition disorders | Oral intake reduced | 179 | 2.47  (2.04 - 2.98) | 2.47  (93.68) | 1.88  (1.60) | 0.91  (0.66) | < 0.001 |
| General disorders and administration site conditions | Injection site discolouration | 178 | 1.58  (1.33 - 1.88) | 1.58  (26.60) | 1.41  (1.21) | 0.49  (0.25) | < 0.001 |
| Respiratory, thoracic and mediastinal disorders | Respiratory disorder | 177 | 1.40  (1.18 - 1.66) | 1.40  (14.50) | 1.29  (1.11) | 0.37  (0.12) | < 0.001 |
| Infections and infestations | Injection site abscess | 170 | 1.23  (1.03 - 1.46) | 1.23  (5.44) | 1.17  (1.01) | 0.23  (-0.02) | 0.030 |
| Vascular disorders | Flushing | 168 | 1.27  (1.07 - 1.52) | 1.27  (7.38) | 1.20  (1.04) | 0.27  (0.02) | 0.012 |
| Infections and infestations | Abscess | 160 | 1.94  (1.60 - 2.35) | 1.94  (47.65) | 1.62  (1.38) | 0.69  (0.43) | < 0.001 |
| General disorders and administration site conditions | Abasia | 147 | 1.39  (1.15 - 1.67) | 1.39  (11.46) | 1.28  (1.09) | 0.36  (0.09) | 0.002 |
| Eye disorders | Eyelid oedema | 141 | 1.42  (1.17 - 1.72) | 1.42  (12.62) | 1.30  (1.11) | 0.38  (0.11) | 0.001 |
| Infections and infestations | Pertussis | 140 | 1.55  (1.27 - 1.89) | 1.55  (19.17) | 1.39  (1.18) | 0.47  (0.19) | < 0.001 |
| General disorders and administration site conditions | Difficulty in walking | 117 | 3.23  (2.52 - 4.13) | 3.23  (95.93) | 2.19  (1.78) | 1.13  (0.81) | < 0.001 |
| General disorders and administration site conditions | Injection site haemorrhage | 115 | 1.91  (1.52 - 2.39) | 1.90  (32.63) | 1.60  (1.32) | 0.68  (0.37) | < 0.001 |
| General disorders and administration site conditions | Injection site bruising | 113 | 1.31  (1.06 - 1.62) | 1.31  (6.07) | 1.23  (1.03) | 0.30  (-0.01) | 0.023 |
| Nervous system disorders | Nervous system disorder | 110 | 1.29  (1.04 - 1.60) | 1.29  (5.32) | 1.21  (1.01) | 0.28  (-0.03) | 0.032 |
| General disorders and administration site conditions | Extensive swelling of vaccinated limb | 103 | 1.71  (1.36 - 2.17) | 1.71  (20.93) | 1.49  (1.22) | 0.57  (0.25) | < 0.001 |
| Skin and subcutaneous tissue disorders | Cold sweat | 101 | 1.32  (1.05 - 1.66) | 1.32  (5.87) | 1.24  (1.02) | 0.31  (-0.01) | 0.025 |
| Nervous system disorders | Infantile spasms | 91 | 1.61  (1.26 - 2.06) | 1.61  (14.59) | 1.42  (1.16) | 0.51  (0.17) | < 0.001 |
| Injury, poisoning and procedural complications | Injury | 90 | 2.00  (1.55 - 2.59) | 2.00  (29.31) | 1.65  (1.33) | 0.72  (0.37) | < 0.001 |
| General disorders and administration site conditions | High-pitched crying | 87 | 2.30  (1.76 – 3.00) | 2.30  (39.24) | 1.80  (1.44) | 0.85  (0.49) | < 0.001 |
| Nervous system disorders | Postictal state | 85 | 1.38  (1.08 - 1.77) | 1.38  (6.57) | 1.28  (1.04) | 0.35  (0.00) | 0.018 |
| Injury, poisoning and procedural complications | Drug toxicity | 83 | 2.32  (1.77 - 3.06) | 2.32  (38.39) | 1.81  (1.44) | 0.86  (0.49) | < 0.001 |
| Respiratory, thoracic and mediastinal disorders | Hypoventilation | 81 | 1.35  (1.05 - 1.75) | 1.35  (5.48) | 1.26  (1.02) | 0.33  (-0.03) | 0.030 |
| Gastrointestinal disorders | Gastrointestinal haemorrhage | 80 | 2.79  (2.09 - 3.73) | 2.79  (52.28) | 2.02  (1.58) | 1.01  (0.63) | < 0.001 |
| Eye disorders | Eye oedema | 78 | 3.89  (2.83 - 5.35) | 3.89  (81.67) | 2.41  (1.85) | 1.27  (0.87) | < 0.001 |
| Respiratory, thoracic and mediastinal disorders | Pharyngolaryngeal pain | 77 | 1.51  (1.15 - 1.96) | 1.50  (9.27) | 1.36  (1.09) | 0.44  (0.07) | 0.005 |
| Skin and subcutaneous tissue disorders | Angioneurotic oedema | 74 | 2.68  (1.99 - 3.62) | 2.68  (45.17) | 1.97  (1.54) | 0.98  (0.58) | < 0.001 |
| Surgical and medical procedures | Cold compress therapy | 67 | 8.25  (5.36 - 12.69) | 8.25  (132.03) | 3.24  (2.26) | 1.70  (1.24) | < 0.001 |
| General disorders and administration site conditions | Injection site haematoma | 67 | 1.48  (1.12 - 1.97) | 1.48  (7.5) | 1.34  (1.06) | 0.43  (0.03) | 0.012 |
| General disorders and administration site conditions | Injection site extravasation | 65 | 2.27  (1.66 - 3.09) | 2.27  (28.48) | 1.78  (1.38) | 0.84  (0.42) | < 0.001 |
| Infections and infestations | Injection site infection | 60 | 1.51  (1.12 - 2.04) | 1.51  (7.29) | 1.36  (1.06) | 0.44  (0.02) | 0.014 |
| General disorders and administration site conditions | No reaction on previous exposure to drug | 59 | 1.40  (1.04 - 1.89) | 1.40  (4.83) | 1.29  (1.00) | 0.37  (-0.06) | 0.042 |
| Respiratory, thoracic and mediastinal disorders | Grunting | 58 | 1.47  (1.08 - 1.99) | 1.47  (6.18) | 1.33  (1.03) | 0.42  (-0.01) | 0.023 |
| General disorders and administration site conditions | Injection site movement impairment | 56 | 1.42  (1.04 - 1.93) | 1.42  (4.96) | 1.30  (1.01) | 0.38  (-0.05) | 0.039 |
| Skin and subcutaneous tissue disorders | Skin reaction | 53 | 1.72  (1.24 - 2.38) | 1.72  (10.84) | 1.49  (1.13) | 0.58  (0.12) | 0.002 |
| General disorders and administration site conditions | Similar reaction on previous exposure to drug | 50 | 1.71  (1.22 - 2.39) | 1.71  (10.08) | 1.49  (1.12) | 0.57  (0.11) | 0.004 |
| Skin and subcutaneous tissue disorders | Skin tightness | 49 | 2.55  (1.77 - 3.67) | 2.55  (27.30) | 1.92  (1.41) | 0.94  (0.45) | < 0.001 |
| Psychiatric disorders | Tension | 49 | 2.83  (1.95 - 4.10) | 2.83  (32.8) | 2.04  (1.49) | 1.03  (0.54) | < 0.001 |
| Gastrointestinal disorders | Post-tussive vomiting | 48 | 1.42  (1.02 - 1.98) | 1.42  (4.28) | 1.30  (0.99) | 0.38  (-0.09) | 0.052 |
| Eye disorders | Eye rolling | 47 | 2.41  (1.67 - 3.48) | 2.41  (23.49) | 1.85  (1.36) | 0.89  (0.40) | < 0.001 |
| Skin and subcutaneous tissue disorders | Skin irritation | 46 | 2.07  (1.44 - 2.97) | 2.07  (16.35) | 1.69  (1.25) | 0.75  (0.26) | < 0.001 |
| Musculoskeletal and connective tissue disorders | Joint range of motion decreased | 45 | 2.31  (1.59 - 3.35) | 2.31  (20.55) | 1.81  (1.32) | 0.85  (0.35) | < 0.001 |
| Respiratory, thoracic and mediastinal disorders | Pulmonary congestion | 43 | 4.18  (2.7 - 6.47) | 4.18  (48.81) | 2.49  (1.73) | 1.32  (0.78) | < 0.001 |
| Musculoskeletal and connective tissue disorders | Torticollis | 42 | 2.63  (1.77 - 3.91) | 2.63  (24.78) | 1.95  (1.40) | 0.96  (0.44) | < 0.001 |
| Nervous system disorders | Syncope vasovagal | 40 | 4.93  (3.07 - 7.91) | 4.93  (53.63) | 2.68  (1.80) | 1.42  (0.86) | < 0.001 |
| Immune system disorders | Type iii immune complex mediated reaction | 40 | 1.94  (1.33 - 2.85) | 1.94  (12.02) | 1.62  (1.17) | 0.69  (0.17) | 0.002 |
| Nervous system disorders | Sensory disturbance | 39 | 1.50  (1.03 - 2.18) | 1.50  (4.63) | 1.36  (0.99) | 0.44  (-0.08) | 0.047 |
| Metabolism and nutrition disorders | Acidosis | 37 | 1.52  (1.04 - 2.23) | 1.52  (4.64) | 1.37  (0.99) | 0.45  (-0.08) | 0.047 |
| Respiratory, thoracic and mediastinal disorders | Pharyngeal oedema | 36 | 1.56  (1.06 - 2.31) | 1.56  (5.15) | 1.40  (1.01) | 0.48  (-0.06) | 0.038 |
| Injury, poisoning and procedural complications | Wrong technique in drug usage process | 36 | 2.25  (1.49 - 3.41) | 2.25  (15.6) | 1.78  (1.26) | 0.83  (0.27) | < 0.001 |
| Blood and lymphatic system disorders | Lymph node pain | 29 | 1.82  (1.16 - 2.83) | 1.82  (7.12) | 1.55  (1.07) | 0.63  (0.02) | 0.017 |
| Eye disorders | Visual disturbance | 28 | 1.59  (1.02 - 2.48) | 1.59  (4.30) | 1.41  (0.98) | 0.50  (-0.11) | 0.055 |
| Surgical and medical procedures | Oxygen supplementation | 28 | 2.15  (1.35 - 3.43) | 2.15  (10.95) | 1.73  (1.17) | 0.79  (0.16) | 0.003 |
| Skin and subcutaneous tissue disorders | Skin striae | 27 | 1.96  (1.23 - 3.12) | 1.96  (8.24) | 1.62  (1.10) | 0.70  (0.07) | 0.010 |
| Infections and infestations | Erythema induratum | 22 | 11.61  (4.96 - 27.18) | 11.61  (51.49) | 3.56  (1.75) | 1.83  (1.03) | < 0.001 |
| Musculoskeletal and connective tissue disorders | Musculoskeletal pain | 21 | 2.77  (1.57 - 4.88) | 2.77  (13.58) | 2.01  (1.25) | 1.01  (0.27) | 0.001 |
| General disorders and administration site conditions | Nonspecific reaction | 20 | 2.55  (1.44 - 4.5) | 2.55  (11.13) | 1.92  (1.19) | 0.94  (0.19) | 0.003 |
| Psychiatric disorders | Affect lability | 19 | 1.90  (1.09 - 3.3) | 1.90  (5.32) | 1.59  (1.00) | 0.67  (-0.08) | 0.039 |
| Investigations | Blood pressure | 19 | 3.34  (1.80 - 6.22) | 3.34  (16.37) | 2.23  (1.33) | 1.16  (0.37) | < 0.001 |
| General disorders and administration site conditions | Cyst | 18 | 2.01  (1.13 - 3.58) | 2.01  (5.95) | 1.66  (1.02) | 0.73  (-0.05) | 0.030 |
| Gastrointestinal disorders | Oral mucosal blistering | 17 | 2.03  (1.12 - 3.66) | 2.03  (5.70) | 1.66  (1.01) | 0.73  (-0.06) | 0.034 |
| Nervous system disorders | Sensory processing disorder | 15 | 2.22  (1.17 - 4.20) | 2.22  (6.26) | 1.76  (1.03) | 0.82  (-0.03) | 0.029 |
| Skin and subcutaneous tissue disorders | Skin burning sensation | 15 | 1.98  (1.06 - 3.71) | 1.98  (4.73) | 1.64  (0.97) | 0.71  (-0.13) | 0.051 |
| Nervous system disorders | Asterixis | 13 | 2.09  (1.06 - 4.12) | 2.09  (4.71) | 1.70  (0.96) | 0.76  (-0.14) | 0.053 |
| General disorders and administration site conditions | Injection site abscess sterile | 13 | 2.53  (1.25 - 5.12) | 2.53  (7.13) | 1.91  (1.06) | 0.93  (0.01) | 0.020 |
| Vascular disorders | Lymphoedema | 12 | 3.17  (1.46 - 6.85) | 3.17  (9.58) | 2.17  (1.14) | 1.12  (0.14) | 0.007 |
| Respiratory, thoracic and mediastinal disorders | Respiratory depression | 12 | 2.11  (1.04 - 4.29) | 2.11  (4.46) | 1.71  (0.94) | 0.77  (-0.17) | 0.061 |
| Investigations | Pertussis identification test positive | 12 | 6.33  (2.49 - 16.09) | 6.33  (19.85) | 2.96  (1.36) | 1.57  (0.54) | < 0.001 |
| Product issues | Pharmaceutical product complaint | 9 | 5.54  (1.97 - 15.57) | 5.54  (13.40) | 2.82  (1.19) | 1.49  (0.33) | 0.002 |
| Congenital, familial and genetic disorders | Chromosome abnormality | 8 | 3.28  (1.27 - 8.51) | 3.28  (6.73) | 2.21  (1.00) | 1.14  (-0.03) | 0.029 |
| Psychiatric disorders | Self injurious behaviour | 8 | 3.69  (1.39 - 9.84) | 3.69  (7.86) | 2.35  (1.03) | 1.23  (0.04) | 0.018 |
| Neoplasms benign, malignant and unspecified (incl cysts and polyps) | Skin papilloma | 8 | 3.69  (1.39 - 9.84) | 3.69  (7.86) | 2.35  (1.03) | 1.23  (0.04) | 0.018 |
| Surgical and medical procedures | Heat therapy | 8 | 3.69  (1.39 - 9.84) | 3.69  (7.86) | 2.35  (1.03) | 1.23  (0.04) | 0.018 |
| Investigations | Bordetella test | 8 | 3.28  (1.27 - 8.51) | 3.28  (6.73) | 2.21  (1.00) | 1.14  (-0.03) | 0.029 |
| Gastrointestinal disorders | Tooth disorder | 7 | 3.69  (1.30 - 10.53) | 3.69  (6.88) | 2.35  (0.98) | 1.23  (-0.03) | 0.029 |
| Congenital, familial and genetic disorders | Tourette's disorder | 7 | 5.17  (1.64 - 16.29) | 5.17  (9.81) | 2.74  (1.05) | 1.45  (0.16) | 0.009 |
| Respiratory, thoracic and mediastinal disorders | Nasal oedema | 7 | 2.59  (0.98 - 6.79) | 2.59  (4.00) | 1.93  (0.86) | 0.95  (-0.28) | 0.092 |
| General disorders and administration site conditions | Injection site dermatitis | 7 | 2.87  (1.07 - 7.71) | 2.87  (4.81) | 2.05  (0.90) | 1.04  (-0.20) | 0.063 |
| Pregnancy, puerperium and perinatal conditions | Premature labour | 6 | 11.08  (2.24 - 54.91) | 11.08  (13.76) | 3.52  (0.92) | 1.82  (0.36) | 0.002 |
| Gastrointestinal disorders | Glossitis | 6 | 3.69  (1.19 - 11.45) | 3.69  (5.89) | 2.35  (0.91) | 1.23  (-0.12) | 0.044 |
| Investigations | Carbon dioxide abnormal | 6 | 7.39  (1.85 - 29.54) | 7.39  (11.05) | 3.13  (0.98) | 1.65  (0.22) | 0.006 |
| Psychiatric disorders | Asocial behaviour | 6 | 3.17  (1.06 - 9.42) | 3.17  (4.79) | 2.17  (0.87) | 1.12  (-0.22) | 0.067 |
| Investigations | Blood heavy metal test | 6 | 5.54  (1.56 - 19.64) | 5.54  (8.93) | 2.82  (0.98) | 1.49  (0.10) | 0.014 |
| Investigations | Electron microscopy | 6 | 7.39  (1.85 - 29.54) | 7.39  (11.05) | 3.13  (0.98) | 1.65  (0.22) | 0.006 |
| Investigations | Blood urea nitrogen/creatinine ratio increased | 5 | 4.62  (1.24 - 17.2) | 4.62  (6.30) | 2.61  (0.87) | 1.38  (-0.11) | 0.042 |
| General disorders and administration site conditions | Injection site lymphadenopathy | 5 | 4.62  (1.24 - 17.2) | 4.62  (6.30) | 2.61  (0.87) | 1.38  (-0.11) | 0.042 |
| Investigations | Blood prolactin normal | 5 | 4.62  (1.24 - 17.2) | 4.62  (6.30) | 2.61  (0.87) | 1.38  (-0.11) | 0.042 |
| Endocrine disorders | Adrenal disorder | 4 | 7.39  (1.35 - 40.34) | 7.39  (7.36) | 3.13  (0.76) | 1.65  (-0.04) | 0.034 |
| Respiratory, thoracic and mediastinal disorders | Anoxia | 4 | 7.39  (1.35 - 40.34) | 7.39  (7.36) | 3.13  (0.76) | 1.65  (-0.04) | 0.034 |
| Skin and subcutaneous tissue disorders | Lipoatrophy | 4 | 7.39  (1.35 - 40.34) | 7.39  (7.36) | 3.13  (0.76) | 1.65  (-0.04) | 0.034 |
| General disorders and administration site conditions | Injection site calcification | 4 | 7.39  (1.35 - 40.34) | 7.39  (7.36) | 3.13  (0.76) | 1.65  (-0.04) | 0.034 |
| Skin and subcutaneous tissue disorders | Piloerection | 4 | 4.93  (1.10 - 22.01) | 4.93  (5.36) | 2.68  (0.77) | 1.42  (-0.22) | 0.067 |
| Investigations | Blood acid phosphatase increased | 3 | 11.08  (1.15 - 106.54) | 11.08  (6.88) | 3.52  (0.53) | 1.82  (-0.12) | 0.049 |
| Gastrointestinal disorders | Vomiting neonatal | 3 | 11.08  (1.15 - 106.54) | 11.08  (6.88) | 3.52  (0.53) | 1.82  (-0.12) | 0.049 |
| General disorders and administration site conditions | Sensation of heaviness | 3 | 5.54  (0.93 - 33.16) | 5.54  (4.47) | 2.82  (0.63) | 1.49  (-0.37) | 0.117 |
| Investigations | Pertussis identification test negative | 3 | 11.08  (1.15 - 106.54) | 11.08  (6.88) | 3.52  (0.53) | 1.82  (-0.12) | 0.049 |
| Investigations | Blood pressure systolic | 3 | 11.08  (1.15 - 106.54) | 11.08  (6.88) | 3.52  (0.53) | 1.82  (-0.12) | 0.049 |
| Reproductive system and breast disorders | Genital swelling | 3 | 11.08  (1.15 - 106.54) | 11.08  (6.88) | 3.52  (0.53) | 1.82  (-0.12) | 0.049 |
| Cardiac disorders | Wolff-parkinson-white syndrome | 3 | 5.54  (0.93 - 33.16) | 5.54  (4.47) | 2.82  (0.63) | 1.49  (-0.37) | 0.117 |
| Blood and lymphatic system disorders | Aplasia pure red cell | 3 | 5.54  (0.93 - 33.16) | 5.54  (4.47) | 2.82  (0.63) | 1.49  (-0.37) | 0.117 |
| Investigations | Respiratory syncytial virus serology | 3 | 5.54  (0.93 - 33.16) | 5.54  (4.47) | 2.82  (0.63) | 1.49  (-0.37) | 0.117 |
| Social circumstances | Hearing aid user | 3 | 5.54  (0.93 - 33.16) | 5.54  (4.47) | 2.82  (0.63) | 1.49  (-0.37) | 0.117 |
| General disorders and administration site conditions | Injection site joint movement impairment | 3 | 5.54  (0.93 - 33.16) | 5.54  (4.47) | 2.82  (0.63) | 1.49  (-0.37) | 0.117 |
| Investigations | Gastrointestinal examination | 3 | 11.08  (1.15 - 106.54) | 11.08  (6.88) | 3.52  (0.53) | 1.82  (-0.12) | 0.049 |

DTaP: diphtheria, tetanus, and acellular pertussis; PT, preferred term; SOC, System Organ Class; CI, confidence interval; ROR, reporting odds ratio; PRR, proportional reporting ratio; χ^2^, Chi-squared; IC, information component; IC-2SD, the lower limit of the 95% two-sided CI of the IC; EBGM, empirical Bayesian geometric mean; EBGM05, the lower 95 two-sided CI of EBGM.

# Table S4. Signal strength of AE reports of DTaP vaccines at the SOC level.

| SOC | a | ROR  (95%Cl) | PRR  (χ^2^) | EBGM  (EBGM05) | IC  (IC-2SD) |
| --- | --- | --- | --- | --- | --- |
| General disorders and administration site conditions | 87112 | 2.04  (2.02 - 2.06) | 1.57  (18796.43) | 1.40  (1.39) | 0.49  (0.48) |
| Skin and subcutaneous tissue disorders | 26184 | 1.28  (1.27 - 1.30) | 1.25  (1075.68) | 1.18  (1.17) | 0.24  (0.22) |
| Nervous system disorders | 15856 | 0.90  (0.88 - 0.92) | 0.91  (130.04) | 0.93  (0.91) | -0.11  (-0.14) |
| Investigations | 13624 | 0.45  (0.44 - 0.46) | 0.49  (7385.46) | 0.55  (0.54) | -0.87  (-0.89) |
| Infections and infestations | 8323 | 0.69  (0.68 - 0.71) | 0.71  (899.76) | 0.75  (0.74) | -0.41  (-0.44) |
| Psychiatric disorders | 8322 | 0.95  (0.92 - 0.97) | 0.95  (18.82) | 0.96  (0.94) | -0.06  (-0.09) |
| Gastrointestinal disorders | 8162 | 0.69  (0.67 - 0.70) | 0.70  (936.24) | 0.75  (0.73) | -0.42  (-0.45) |
| Respiratory, thoracic and mediastinal disorders | 5805 | 0.82  (0.80 - 0.84) | 0.83  (182.4) | 0.86  (0.84) | -0.22  (-0.26) |
| Vascular disorders | 4279 | 1.03  (1.00 - 1.07) | 1.03  (3.03) | 1.02  (0.99) | 0.03  (-0.02) |
| Injury, poisoning and procedural complications | 3831 | 0.27  (0.26 - 0.28) | 0.28  (6947.18) | 0.33  (0.32) | -1.59  (-1.64) |
| Musculoskeletal and connective tissue disorders | 2544 | 0.87  (0.84 - 0.91) | 0.88  (36.76) | 0.90  (0.87) | -0.15  (-0.22) |
| Metabolism and nutrition disorders | 2423 | 0.81  (0.77 - 0.84) | 0.81  (89.44) | 0.84  (0.81) | -0.24  (-0.31) |
| Eye disorders | 2169 | 1.06  (1.01 - 1.11) | 1.06  (5.7) | 1.05  (1.01) | 0.07  (0.00) |
| Immune system disorders | 1300 | 1.16  (1.09 - 1.24) | 1.16  (22.73) | 1.12  (1.07) | 0.17  (0.08) |
| Blood and lymphatic system disorders | 1274 | 0.70  (0.66 - 0.74) | 0.70  (138.58) | 0.75  (0.71) | -0.42  (-0.51) |
| Surgical and medical procedures | 1015 | 0.55  (0.52 - 0.59) | 0.55  (318.50) | 0.61  (0.58) | -0.71  (-0.8) |
| Cardiac disorders | 612 | 0.79  (0.73 - 0.87) | 0.79  (26.76) | 0.83  (0.77) | -0.27  (-0.39) |
| Ear and labyrinth disorders | 309 | 0.86  (0.76 - 0.97) | 0.86  (6.00) | 0.88  (0.80) | -0.18  (-0.36) |
| Renal and urinary disorders | 224 | 0.66  (0.57 - 0.76) | 0.66  (34.05) | 0.71  (0.63) | -0.50  (-0.70) |
| Congenital, familial and genetic disorders | 175 | 0.81  (0.69 - 0.95) | 0.81  (6.48) | 0.84  (0.74) | -0.25  (-0.48) |
| Social circumstances | 91 | 0.46  (0.37 - 0.57) | 0.46  (50.60) | 0.52  (0.44) | -0.94  (-1.25) |
| Hepatobiliary disorders | 77 | 0.35  (0.28 - 0.44) | 0.35  (84.61) | 0.41  (0.33) | -1.30  (-1.64) |
| Reproductive system and breast disorders | 77 | 1.01  (0.78 - 1.29) | 1.01  (0.00) | 1.00  (0.81) | 0.01  (-0.35) |
| Neoplasms benign, malignant and unspecified  (incl cysts and polyps) | 61 | 0.67  (0.51 - 0.89) | 0.67  (8.10) | 0.72  (0.58) | -0.46  (-0.86) |
| Product issues | 49 | 0.26  (0.19 - 0.34) | 0.26  (99.65) | 0.30  (0.24) | -1.72  (-2.14) |
| Pregnancy, puerperium and perinatal conditions | 29 | 0.97  (0.64 - 1.45) | 0.97  (0.03) | 0.97  (0.69) | -0.04  (-0.62) |
| Endocrine disorders | 26 | 0.82  (0.54 - 1.26) | 0.82  (0.83) | 0.85  (0.60) | -0.23  (-0.83) |

AE, adverse event; DTaP: diphtheria, tetanus, and acellular pertussis; CI, confidence interval; SOC, System Organ Class; ROR, reporting odds ratio; PRR, proportional reporting ratio; χ^2^, Chi-squared; IC, information component; IC-2SD, the lower limit of the 95% two-sided CI of the IC; EBGM, empirical Bayesian geometric mean; EBGM05, the lower 95 two-sided CI of EBGM.

**A**: Females **B**: Males


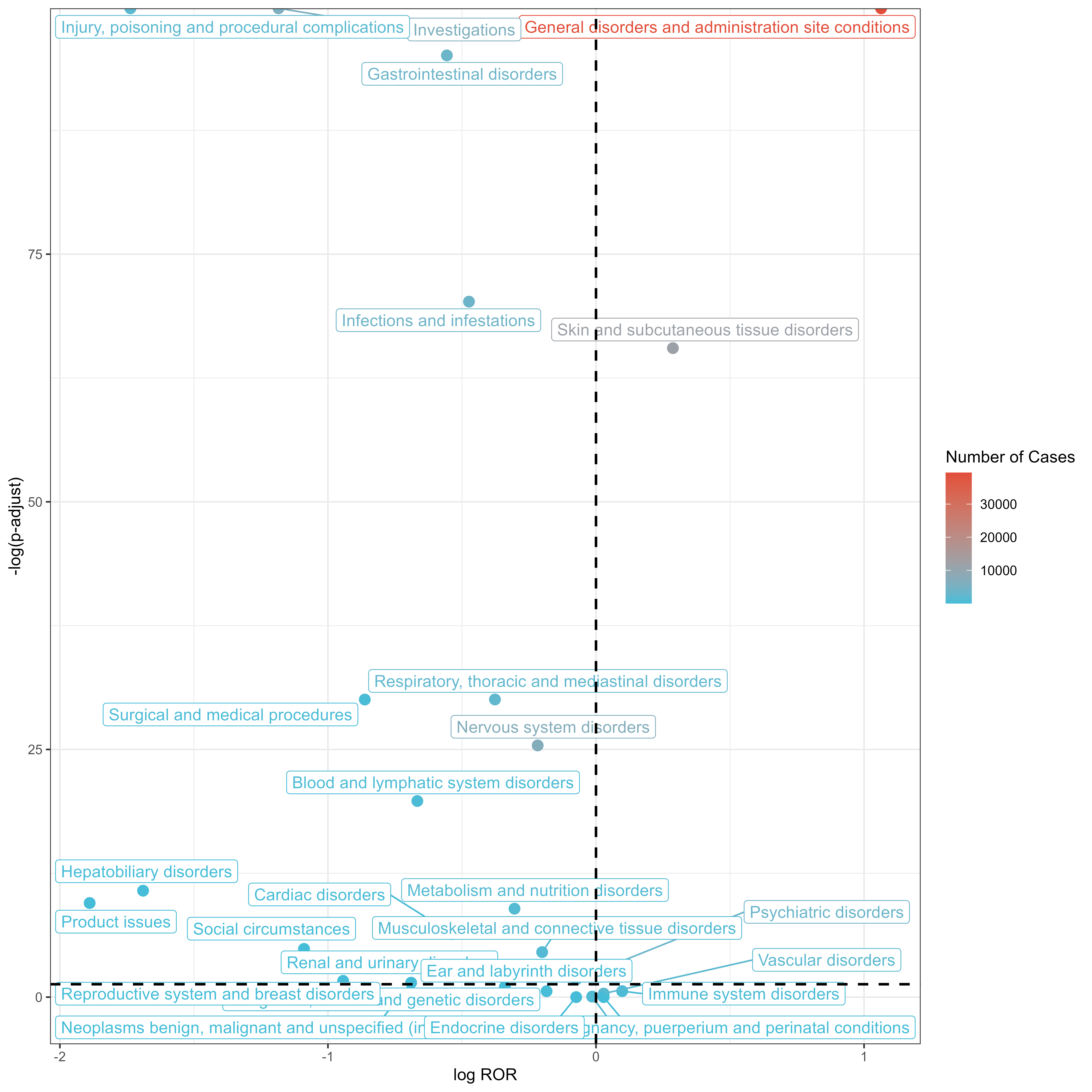

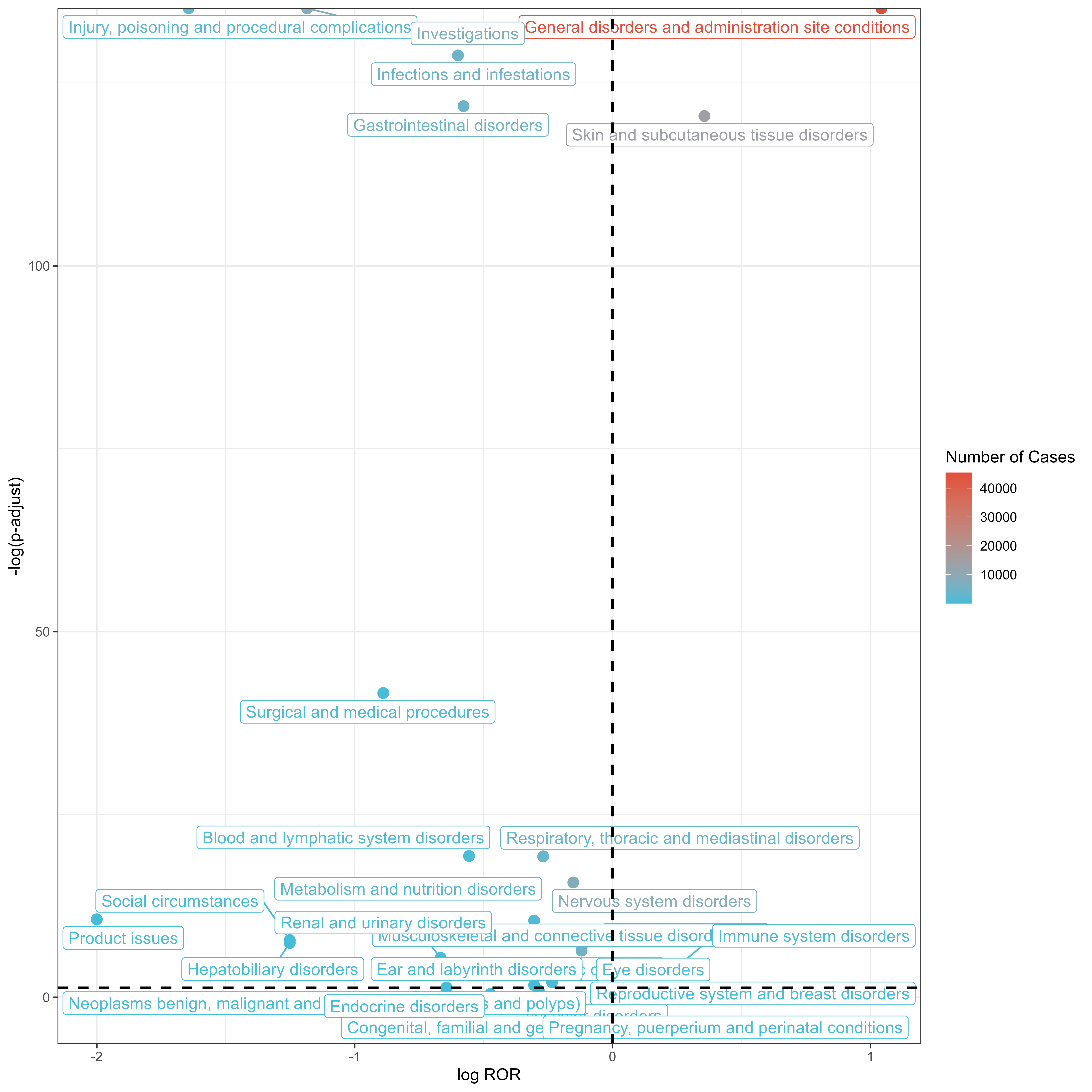


# Figure S1. Volcano plot of SOCs corresponding to reported AEs in females and males.

SOC, system organ classes; AEs, adverse events; ROR, reporting odds ratio.

A: < 2 years B: ≥ 2 and < 5 years C: ≥ 5 and < 7 years


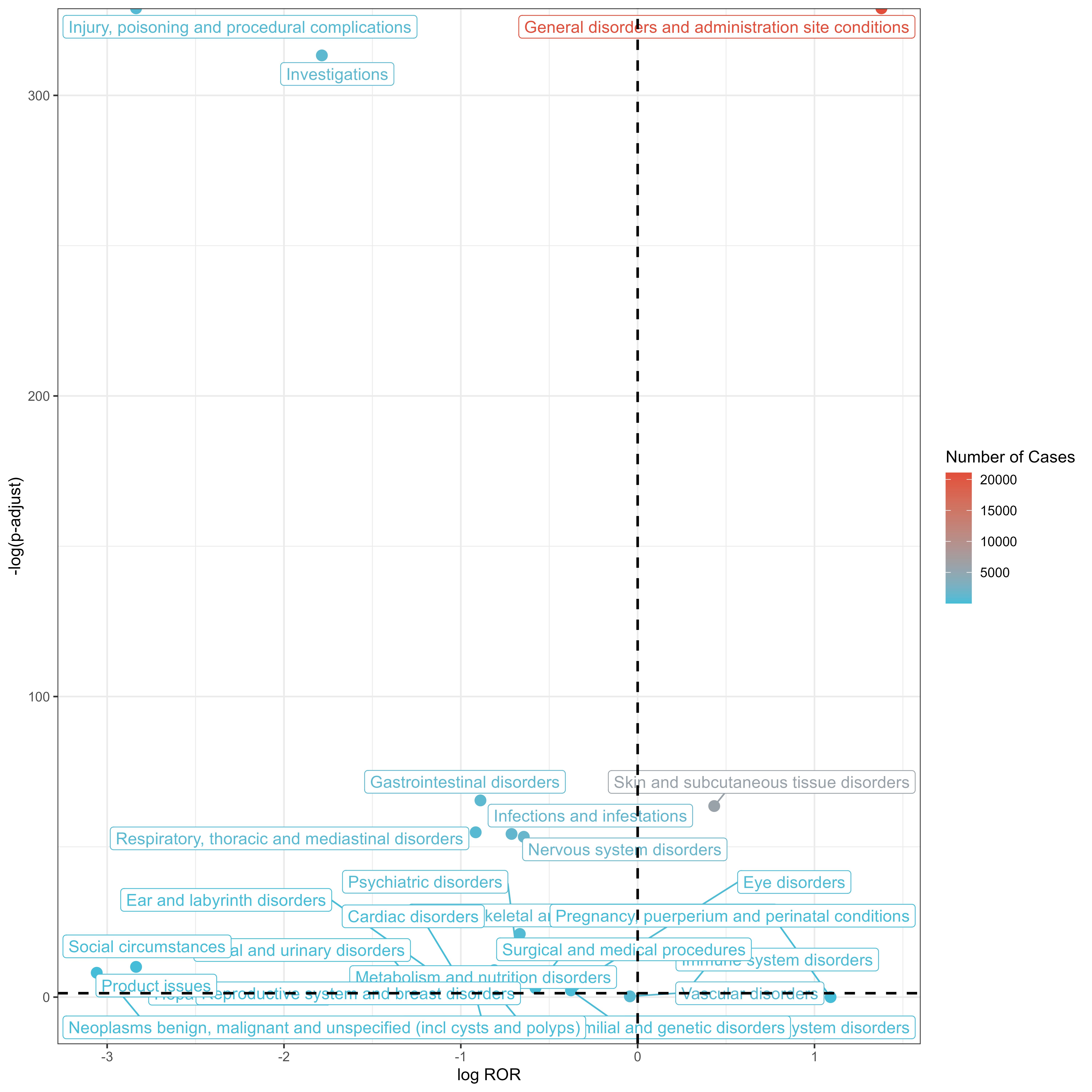

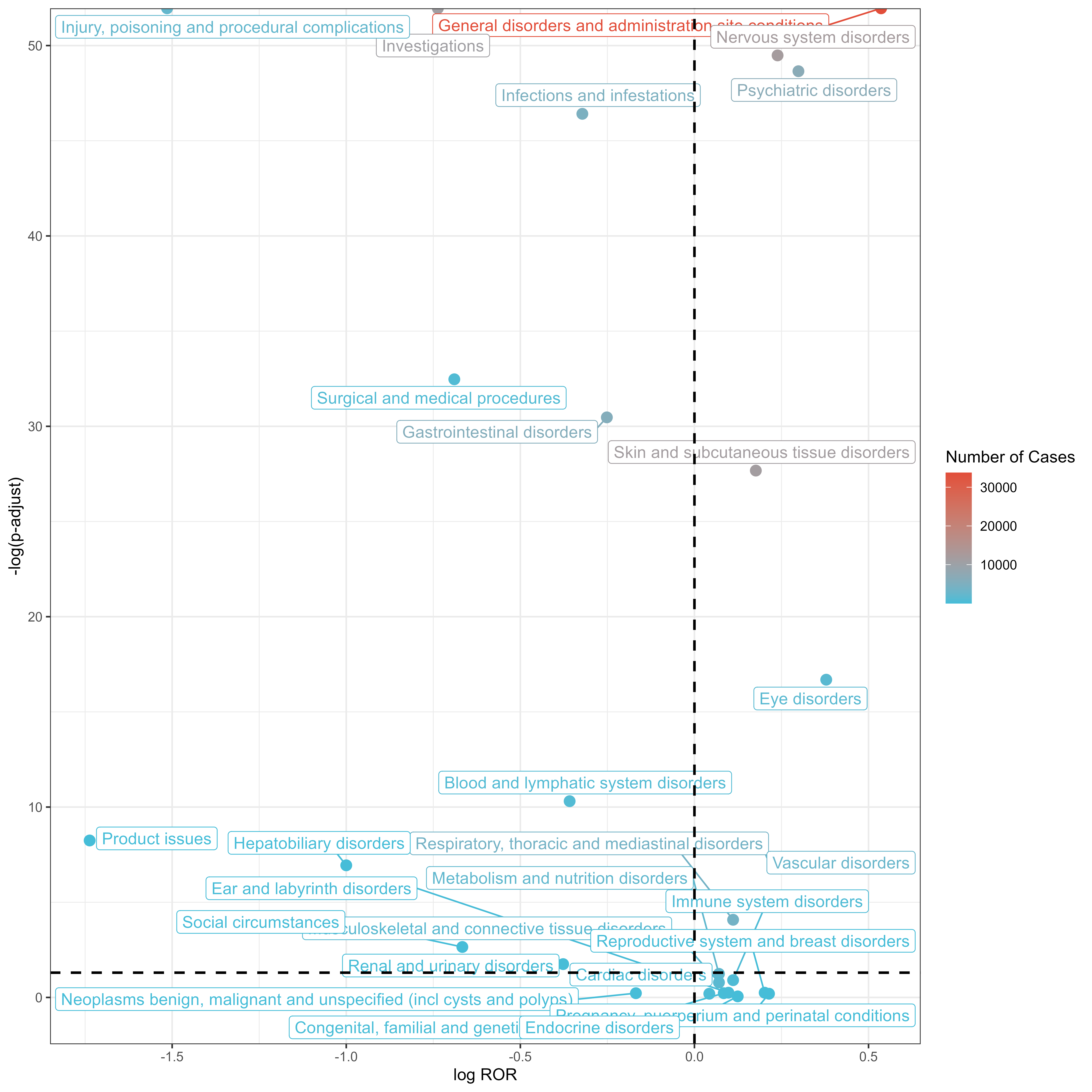

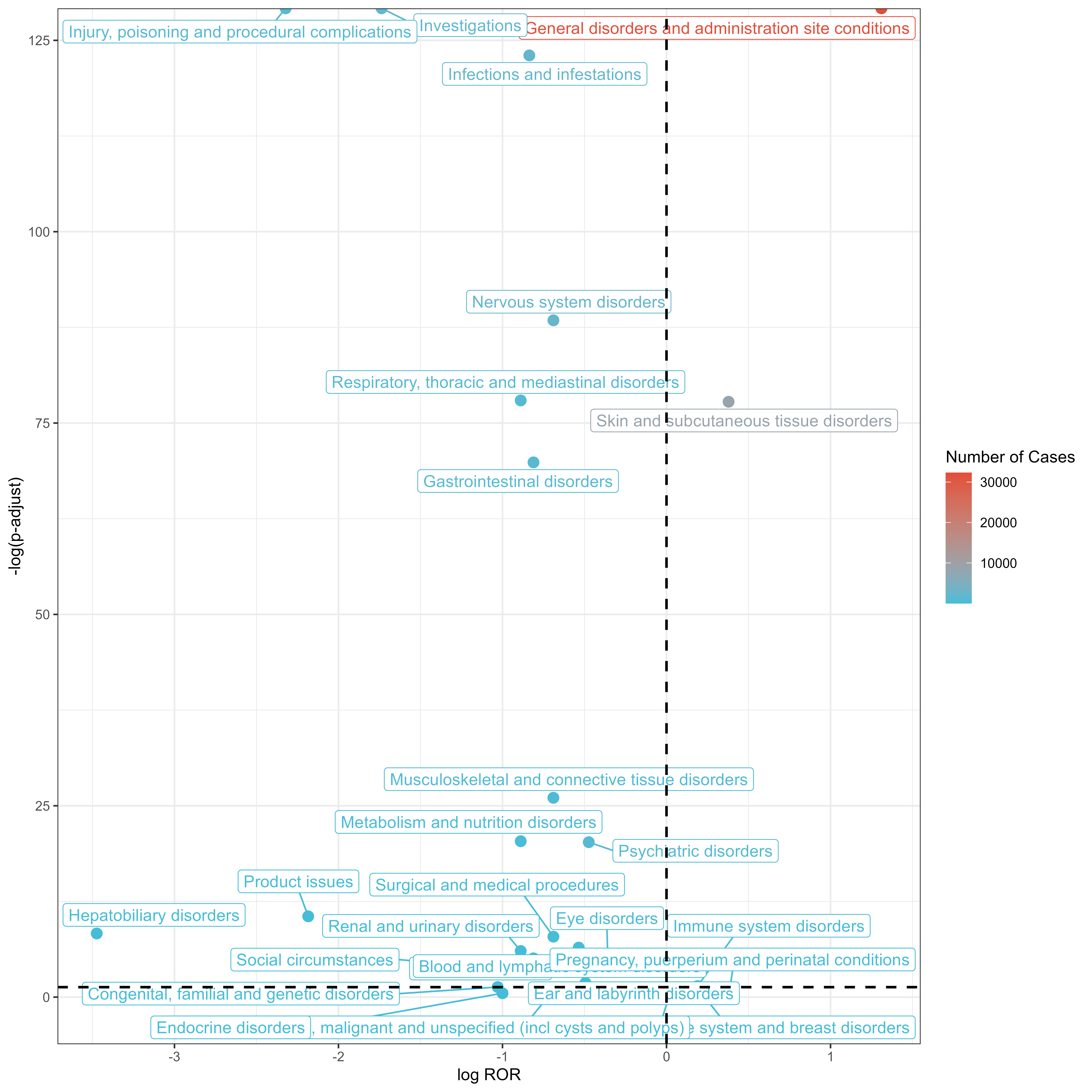


# Figure S2. Volcano plot of SOCs corresponding to reported AEs in < 2, ≥ 2 and < 5, ≥ 5 and < 7 years.

SOC, system organ classes; AEs, adverse events; ROR, reporting odds ratio.

# Table S5. Signal strength of DTaP vaccine-related positive SAE PT signals and their corresponding SOCs

| SOC | PT | a | ROR (95%Cl) | PRR (χ^2^) | EBGM (EBGM05) | IC (IC-2SD) |
| --- | --- | --- | --- | --- | --- | --- |
| Nervous system disorders | Convulsion | 1124 | 1.82  (1.70 - 1.95) | 1.80  (308.55) | 1.61  (1.52) | 0.68  (0.59) |
| General disorders and administration site conditions | Crying | 604 | 1.15  (1.06 - 1.26) | 1.15  (10.20) | 1.13  (1.05) | 0.17  (0.04) |
| Nervous system disorders | Hypotonia | 602 | 1.48  (1.35 - 1.62) | 1.47  (73.17) | 1.38  (1.28) | 0.46  (0.33) |
| Vascular disorders | Pallor | 543 | 1.33  (1.22 - 1.47) | 1.33  (36.53) | 1.27  (1.17) | 0.34  (0.21) |
| Vascular disorders | Cyanosis | 479 | 1.37  (1.24 - 1.51) | 1.36  (37.51) | 1.29  (1.19) | 0.37  (0.23) |
| Respiratory, thoracic and mediastinal disorders | Apnoea | 432 | 1.61  (1.44 - 1.79) | 1.60  (76.75) | 1.47  (1.34) | 0.56  (0.40) |
| General disorders and administration site conditions | Injection site erythema | 400 | 2.44  (2.17 - 2.74) | 2.42  (235.71) | 2.00  (1.81) | 1.00  (0.83) |
| Psychiatric disorders | Irritability | 390 | 1.66  (1.48 - 1.86) | 1.66  (79.05) | 1.51  (1.37) | 0.59  (0.43) |
| Investigations | Laboratory test abnormal | 388 | 2.15  (1.91 - 2.42) | 2.14  (171.95) | 1.83  (1.66) | 0.87  (0.7) |
| Investigations | Body temperature increased | 350 | 1.46  (1.30 - 1.64) | 1.45  (39.9) | 1.36  (1.23) | 0.45  (0.28) |
| Nervous system disorders | Somnolence | 340 | 1.24  (1.10 - 1.40) | 1.24  (13.1) | 1.20  (1.09) | 0.26  (0.09) |
| General disorders and administration site conditions | Sudden infant death syndrome | 331 | 1.84  (1.62 - 2.08) | 1.83  (94.78) | 1.63  (1.47) | 0.70  (0.53) |
| Psychiatric disorders | Agitation | 329 | 1.62  (1.43 - 1.83) | 1.62  (60.59) | 1.48  (1.34) | 0.57  (0.39) |
| Nervous system disorders | Lethargy | 286 | 1.71  (1.50 - 1.96) | 1.71  (65.01) | 1.55  (1.38) | 0.63  (0.44) |
| Respiratory, thoracic and mediastinal disorders | Dyspnoea | 279 | 1.14  (1.01 - 1.3) | 1.14  (4.23) | 1.12  (1.01) | 0.16  (-0.02) |
| Skin and subcutaneous tissue disorders | Erythema | 254 | 1.34  (1.16 - 1.53) | 1.33  (17.22) | 1.27  (1.13) | 0.34  (0.15) |
| Infections and infestations | Cellulitis | 241 | 3.28  (2.8 - 3.85) | 3.27  (241.58) | 2.44  (2.14) | 1.29  (1.07) |
| General disorders and administration site conditions | Screaming | 236 | 1.62  (1.40 - 1.87) | 1.61  (43.06) | 1.48  (1.31) | 0.56  (0.36) |
| General disorders and administration site conditions | Injection site swelling | 236 | 2.00  (1.73 - 2.32) | 2.00  (87.26) | 1.74  (1.53) | 0.8  (0.59) |
| Skin and subcutaneous tissue disorders | Urticaria | 228 | 1.57  (1.35 - 1.81) | 1.56  (36.37) | 1.44  (1.27) | 0.53  (0.32) |
| General disorders and administration site conditions | Injection site oedema | 213 | 2.79  (2.37 - 3.29) | 2.78  (163.74) | 2.20  (1.92) | 1.14  (0.91) |
| Psychiatric disorders | Autism | 209 | 1.85  (1.58 - 2.17) | 1.85  (61.61) | 1.64  (1.44) | 0.71  (0.49) |
| Nervous system disorders | Coma | 207 | 4.08  (3.41 - 4.88) | 4.07  (279.64) | 2.79  (2.40) | 1.48  (1.24) |
| Investigations | Electroencephalogram abnormal | 203 | 1.66  (1.42 - 1.94) | 1.66  (41.03) | 1.51  (1.32) | 0.59  (0.37) |
| Nervous system disorders | Stupor | 192 | 1.47  (1.26 - 1.73) | 1.47  (22.99) | 1.37  (1.20) | 0.46  (0.23) |
| General disorders and administration site conditions | Injection site pain | 188 | 1.87  (1.58 - 2.2) | 1.86  (56.69) | 1.65  (1.44) | 0.72  (0.49) |
| Nervous system disorders | Tremor | 184 | 1.38  (1.18 - 1.63) | 1.38  (15.72) | 1.31  (1.14) | 0.39  (0.15) |
| Metabolism and nutrition disorders | Anorexia | 174 | 2.24  (1.88 - 2.67) | 2.24  (85.63) | 1.89  (1.63) | 0.92  (0.67) |
| General disorders and administration site conditions | Oedema peripheral | 173 | 2.31  (1.93 - 2.75) | 2.30  (90.92) | 1.93  (1.66) | 0.95  (0.70) |
| Psychiatric disorders | Staring | 170 | 2.06  (1.72 - 2.45) | 2.05  (67.48) | 1.77  (1.53) | 0.83  (0.58) |
| General disorders and administration site conditions | Injection site reaction | 164 | 2.43  (2.02 - 2.92) | 2.43  (96.58) | 2.00  (1.72) | 1.00  (0.74) |
| Infections and infestations | Viral infection | 162 | 1.70  (1.43 - 2.03) | 1.70  (36.22) | 1.54  (1.33) | 0.62  (0.37) |
| Immune system disorders | Hypersensitivity | 156 | 1.62  (1.36 - 1.94) | 1.62  (28.77) | 1.48  (1.28) | 0.57  (0.31) |
| General disorders and administration site conditions | Condition aggravated | 154 | 1.29  (1.08 - 1.54) | 1.29  (8.29) | 1.24  (1.07) | 0.31  (0.06) |
| Nervous system disorders | Dyskinesia | 148 | 1.55  (1.30 - 1.86) | 1.55  (22.91) | 1.43  (1.23) | 0.52  (0.26) |
| General disorders and administration site conditions | Injection site warmth | 146 | 4.07  (3.29 - 5.03) | 4.06  (196.58) | 2.78  (2.33) | 1.48  (1.19) |
| Psychiatric disorders | Abnormal behaviour | 145 | 1.71  (1.42 - 2.06) | 1.71  (32.68) | 1.54  (1.32) | 0.63  (0.36) |
| Respiratory, thoracic and mediastinal disorders | Hypoxia | 144 | 2.41  (1.98 - 2.93) | 2.40  (83.24) | 1.99  (1.69) | 0.99  (0.72) |
| Infections and infestations | Bacterial infection | 143 | 2.16  (1.78 - 2.62) | 2.15  (64.3) | 1.84  (1.56) | 0.88  (0.61) |
| Eye disorders | Eye movement disorder | 132 | 1.70  (1.40 - 2.07) | 1.70  (29.41) | 1.54  (1.31) | 0.62  (0.34) |
| Infections and infestations | Otitis media | 131 | 1.40  (1.16 - 1.7) | 1.40  (12.03) | 1.32  (1.13) | 0.40  (0.13) |
| Musculoskeletal and connective tissue disorders | Musculoskeletal stiffness | 130 | 1.61  (1.33 - 1.96) | 1.61  (23.44) | 1.48  (1.25) | 0.56  (0.28) |
| Nervous system disorders | Speech disorder | 125 | 1.58  (1.29 - 1.92) | 1.57  (20.6) | 1.45  (1.23) | 0.54  (0.25) |
| General disorders and administration site conditions | Injection site induration | 117 | 3.12  (2.49 - 3.91) | 3.11  (108.66) | 2.37  (1.96) | 1.24  (0.93) |
| Respiratory, thoracic and mediastinal disorders | Wheezing | 116 | 1.77  (1.44 - 2.18) | 1.77  (29.76) | 1.59  (1.33) | 0.67  (0.37) |
| General disorders and administration site conditions | Unevaluable event | 112 | 3.09  (2.45 - 3.89) | 3.08  (102.29) | 2.35  (1.94) | 1.23  (0.92) |
| Cardiac disorders | Cardiac arrest | 109 | 1.40  (1.13 - 1.73) | 1.40  (9.94) | 1.32  (1.11) | 0.40  (0.10) |
| Musculoskeletal and connective tissue disorders | Muscle twitching | 108 | 1.37  (1.11 - 1.69) | 1.37  (8.59) | 1.30  (1.09) | 0.37  (0.07) |
| Nervous system disorders | Aphasia | 103 | 1.73  (1.39 - 2.16) | 1.73  (24.39) | 1.56  (1.30) | 0.64  (0.33) |
| Nervous system disorders | Hypokinesia | 97 | 1.46  (1.17 - 1.83) | 1.46  (11.27) | 1.37  (1.13) | 0.45  (0.13) |
| Nervous system disorders | Grand mal convulsion | 96 | 1.42  (1.14 - 1.78) | 1.42  (9.70) | 1.34  (1.11) | 0.42  (0.10) |
| Nervous system disorders | Depressed level of consciousness | 96 | 1.78  (1.41 - 2.23) | 1.78  (24.81) | 1.59  (1.31) | 0.67  (0.34) |
| Skin and subcutaneous tissue disorders | Skin discolouration | 92 | 1.41  (1.12 - 1.77) | 1.40  (8.62) | 1.32  (1.09) | 0.41  (0.08) |
| General disorders and administration site conditions | Oedema | 92 | 2.11  (1.66 - 2.68) | 2.10  (38.97) | 1.81  (1.48) | 0.85  (0.52) |
| Gastrointestinal disorders | Gastrointestinal disorder | 90 | 2.04  (1.60 - 2.59) | 2.03  (34.90) | 1.76  (1.44) | 0.82  (0.48) |
| Nervous system disorders | Epilepsy | 90 | 1.35  (1.07 - 1.70) | 1.35  (6.58) | 1.28  (1.06) | 0.36  (0.03) |
| Respiratory, thoracic and mediastinal disorders | Respiratory disorder | 87 | 1.72  (1.35 - 2.19) | 1.72  (20.18) | 1.55  (1.27) | 0.64  (0.29) |
| Nervous system disorders | Neurodevelopmental disorder | 87 | 3.04  (2.35 - 3.95) | 3.04  (77.71) | 2.33  (1.87) | 1.22  (0.86) |
| Immune system disorders | Anaphylactic reaction | 86 | 1.58  (1.24 - 2.01) | 1.58  (14.35) | 1.45  (1.19) | 0.54  (0.20) |
| Blood and lymphatic system disorders | Anaemia | 83 | 1.28  (1.01 - 1.62) | 1.28  (4.09) | 1.23  (1.00) | 0.29  (-0.05) |
| Nervous system disorders | Encephalopathy | 81 | 1.40  (1.10 - 1.79) | 1.40  (7.51) | 1.32  (1.08) | 0.40  (0.05) |
| Cardiac disorders | Bradycardia | 81 | 1.51  (1.18 - 1.93) | 1.51  (11.13) | 1.40  (1.14) | 0.49  (0.14) |
| Metabolism and nutrition disorders | Oral intake reduced | 80 | 3.90  (2.93 - 5.18) | 3.89  (102.26) | 2.72  (2.14) | 1.44  (1.06) |
| Nervous system disorders | Nervous system disorder | 79 | 2.33  (1.80 - 3.03) | 2.33  (42.65) | 1.94  (1.56) | 0.96  (0.59) |
| General disorders and administration site conditions | Swelling | 77 | 1.32  (1.03 - 1.69) | 1.32  (4.78) | 1.26  (1.02) | 0.33  (-0.03) |
| Nervous system disorders | Mental retardation severity unspecified | 76 | 1.68  (1.30 - 2.17) | 1.68  (16.09) | 1.52  (1.23) | 0.61  (0.24) |
| General disorders and administration site conditions | Injection site hypersensitivity | 75 | 3.03  (2.29 - 4.01) | 3.03  (66.61) | 2.32  (1.84) | 1.22  (0.83) |
| Nervous system disorders | Infantile spasms | 74 | 2.47  (1.88 - 3.24) | 2.46  (44.99) | 2.02  (1.61) | 1.02  (0.64) |
| Psychiatric disorders | Insomnia | 71 | 1.73  (1.33 - 2.26) | 1.73  (16.72) | 1.56  (1.25) | 0.64  (0.26) |
| Respiratory, thoracic and mediastinal disorders | Asthma | 69 | 1.33  (1.03 - 1.73) | 1.33  (4.64) | 1.27  (1.02) | 0.34  (-0.03) |
| Skin and subcutaneous tissue disorders | Pruritus | 69 | 1.54  (1.18 - 2.01) | 1.54  (10.33) | 1.43  (1.14) | 0.51  (0.13) |
| General disorders and administration site conditions | Feeling hot | 69 | 2.31  (1.75 - 3.06) | 2.31  (36.55) | 1.93  (1.53) | 0.95  (0.56) |
| General disorders and administration site conditions | Developmental delay | 68 | 1.66  (1.26 - 2.17) | 1.65  (13.67) | 1.51  (1.20) | 0.59  (0.21) |
| General disorders and administration site conditions | Injection site mass | 62 | 2.58  (1.91 - 3.48) | 2.58  (41.23) | 2.09  (1.62) | 1.06  (0.64) |
| Respiratory, thoracic and mediastinal disorders | Respiratory distress | 61 | 1.41  (1.06 - 1.86) | 1.41  (5.75) | 1.33  (1.05) | 0.41  (0.01) |
| Nervous system disorders | Speech disorder developmental | 61 | 2.15  (1.60 - 2.88) | 2.14  (27.09) | 1.83  (1.43) | 0.87  (0.46) |
| Psychiatric disorders | Listless | 59 | 1.61  (1.20 - 2.15) | 1.61  (10.58) | 1.47  (1.16) | 0.56  (0.15) |
| Respiratory, thoracic and mediastinal disorders | Nasal congestion | 57 | 1.92  (1.42 - 2.60) | 1.92  (18.83) | 1.69  (1.31) | 0.76  (0.33) |
| Investigations | Heart rate increased | 57 | 1.52  (1.14 - 2.04) | 1.52  (8.11) | 1.41  (1.11) | 0.50  (0.08) |
| Infections and infestations | Injection site abscess | 56 | 2.10  (1.55 - 2.85) | 2.10  (23.54) | 1.80  (1.39) | 0.85  (0.42) |
| Skin and subcutaneous tissue disorders | Hyperhidrosis | 56 | 1.56  (1.16 - 2.10) | 1.56  (8.93) | 1.44  (1.13) | 0.53  (0.11) |
| Eye disorders | Eye disorder | 56 | 2.02  (1.49 - 2.74) | 2.02  (21.26) | 1.75  (1.36) | 0.81  (0.38) |
| General disorders and administration site conditions | Face oedema | 55 | 1.66  (1.23 - 2.24) | 1.66  (11.12) | 1.51  (1.17) | 0.59  (0.17) |
| Psychiatric disorders | Decreased eye contact | 55 | 1.56  (1.16 - 2.1) | 1.56  (8.65) | 1.44  (1.12) | 0.52  (0.10) |
| Gastrointestinal disorders | Gastrointestinal haemorrhage | 52 | 4.56  (3.17 - 6.57) | 4.56  (80.22) | 2.98  (2.19) | 1.57  (1.10) |
| General disorders and administration site conditions | Injection site inflammation | 52 | 2.55  (1.84 - 3.54) | 2.55  (33.94) | 2.07  (1.58) | 1.05  (0.6) |
| Injury, poisoning and procedural complications | Drug toxicity | 52 | 5.59  (3.81 - 8.20) | 5.59  (98.9) | 3.32  (2.41) | 1.73  (1.24) |
| Gastrointestinal disorders | Salivary hypersecretion | 51 | 1.53  (1.12 - 2.08) | 1.53  (7.36) | 1.42  (1.09) | 0.50  (0.06) |
| Eye disorders | Strabismus | 49 | 1.77  (1.28 - 2.44) | 1.77  (12.44) | 1.58  (1.21) | 0.66  (0.21) |
| Respiratory, thoracic and mediastinal disorders | Pulmonary oedema | 48 | 2.07  (1.49 - 2.88) | 2.07  (19.51) | 1.79  (1.35) | 0.84  (0.37) |
| Vascular disorders | Vasodilatation | 46 | 1.74  (1.25 - 2.41) | 1.73  (10.98) | 1.56  (1.19) | 0.64  (0.18) |
| Immune system disorders | Immune system disorder | 46 | 2.24  (1.59 - 3.15) | 2.24  (22.65) | 1.89  (1.42) | 0.92  (0.44) |
| Skin and subcutaneous tissue disorders | Eczema | 44 | 1.60  (1.14 - 2.23) | 1.60  (7.66) | 1.47  (1.11) | 0.55  (0.08) |
| Infections and infestations | Abscess | 43 | 1.90  (1.34 - 2.68) | 1.90  (13.72) | 1.67  (1.25) | 0.74  (0.26) |
| Gastrointestinal disorders | Gastrooesophageal reflux disease | 43 | 1.80  (1.28 - 2.54) | 1.80  (11.64) | 1.61  (1.21) | 0.69  (0.20) |
| Skin and subcutaneous tissue disorders | Blister | 43 | 1.70  (1.21 - 2.39) | 1.70  (9.56) | 1.54  (1.16) | 0.62  (0.14) |
| Injury, poisoning and procedural complications | Inappropriate schedule of drug administration | 43 | 1.58  (1.13 - 2.22) | 1.58  (7.17) | 1.45  (1.10) | 0.54  (0.06) |
| Injury, poisoning and procedural complications | Injury | 41 | 3.16  (2.16 - 4.63) | 3.16  (38.86) | 2.39  (1.73) | 1.26  (0.74) |
| General disorders and administration site conditions | Vaccine positive rechallenge | 37 | 2.15  (1.47 - 3.14) | 2.15  (16.53) | 1.83  (1.34) | 0.88  (0.35) |
| Nervous system disorders | Cognitive disorder | 37 | 2.40  (1.63 - 3.52) | 2.39  (21.16) | 1.98  (1.44) | 0.99  (0.45) |
| Respiratory, thoracic and mediastinal disorders | Hypoventilation | 36 | 2.01  (1.38 - 2.94) | 2.01  (13.51) | 1.75  (1.27) | 0.80  (0.27) |
| Respiratory, thoracic and mediastinal disorders | Pulmonary congestion | 36 | 7.89  (4.76 - 13.07) | 7.89  (90.79) | 3.89  (2.55) | 1.96  (1.36) |
| Nervous system disorders | Opisthotonus | 35 | 1.70  (1.17 - 2.49) | 1.70  (7.83) | 1.54  (1.12) | 0.62  (0.09) |
| Gastrointestinal disorders | Intestinal obstruction | 35 | 1.63  (1.12 - 2.38) | 1.63  (6.69) | 1.49  (1.09) | 0.58  (0.04) |
| Psychiatric disorders | Antisocial behaviour | 35 | 2.77  (1.85 - 4.15) | 2.77  (26.62) | 2.19  (1.56) | 1.13  (0.58) |
| Nervous system disorders | Postictal state | 34 | 1.96  (1.33 - 2.89) | 1.96  (11.83) | 1.71  (1.24) | 0.78  (0.23) |
| Nervous system disorders | Fontanelle bulging | 34 | 1.56  (1.07 - 2.28) | 1.56  (5.39) | 1.44  (1.05) | 0.53  (-0.01) |
| Nervous system disorders | Hemiparesis | 33 | 1.81  (1.22 - 2.67) | 1.81  (9.03) | 1.61  (1.16) | 0.69  (0.14) |
| Nervous system disorders | Hypersomnia | 33 | 1.62  (1.10 - 2.39) | 1.62  (6.10) | 1.48  (1.07) | 0.57  (0.02) |
| General disorders and administration site conditions | Tenderness | 33 | 2.16  (1.45 - 3.23) | 2.16  (14.91) | 1.84  (1.32) | 0.88  (0.32) |
| Metabolism and nutrition disorders | Acidosis | 32 | 2.12  (1.41 - 3.18) | 2.12  (13.79) | 1.82  (1.29) | 0.86  (0.29) |
| Nervous system disorders | Psychomotor hyperactivity | 32 | 1.57  (1.06 - 2.32) | 1.57  (5.21) | 1.45  (1.04) | 0.53  (-0.02) |
| Psychiatric disorders | Aggression | 32 | 1.64  (1.11 - 2.43) | 1.64  (6.24) | 1.50  (1.08) | 0.58  (0.03) |
| Infections and infestations | Injection site cellulitis | 31 | 1.55  (1.04 - 2.30) | 1.55  (4.74) | 1.43  (1.03) | 0.52  (-0.05) |
| Psychiatric disorders | Social avoidant behaviour | 30 | 1.76  (1.17 - 2.65) | 1.76  (7.54) | 1.58  (1.12) | 0.66  (0.08) |
| General disorders and administration site conditions | Difficulty in walking | 30 | 3.35  (2.13 - 5.26) | 3.35  (31.15) | 2.48  (1.70) | 1.31  (0.70) |
| Respiratory, thoracic and mediastinal disorders | Asphyxia | 29 | 1.92  (1.26 - 2.93) | 1.92  (9.57) | 1.69  (1.19) | 0.76  (0.16) |
| Skin and subcutaneous tissue disorders | Cold sweat | 29 | 1.94  (1.28 - 2.96) | 1.94  (9.90) | 1.70  (1.20) | 0.77  (0.18) |
| General disorders and administration site conditions | No reaction on previous exposure to drug | 28 | 2.75  (1.75 - 4.32) | 2.75  (21.03) | 2.18  (1.49) | 1.12  (0.51) |
| Nervous system disorders | Disturbance in attention | 27 | 2.08  (1.34 - 3.23) | 2.08  (11.07) | 1.79  (1.24) | 0.84  (0.22) |
| Skin and subcutaneous tissue disorders | Skin ulcer | 26 | 1.95  (1.25 - 3.04) | 1.95  (8.95) | 1.71  (1.18) | 0.77  (0.15) |
| Infections and infestations | Sinusitis | 24 | 2.32  (1.44 - 3.73) | 2.32  (12.77) | 1.94  (1.30) | 0.95  (0.30) |
| Nervous system disorders | Sensory disturbance | 24 | 2.53  (1.57 - 4.1) | 2.53  (15.39) | 2.06  (1.38) | 1.04  (0.38) |
| Eye disorders | Eye rolling | 24 | 5.95  (3.36 - 10.53) | 5.94  (48.29) | 3.42  (2.12) | 1.77  (1.06) |
| Eye disorders | Visual disturbance | 23 | 3.45  (2.05 - 5.79) | 3.45  (24.89) | 2.52  (1.64) | 1.34  (0.64) |
| Blood and lymphatic system disorders | Thrombocythaemia | 23 | 2.11  (1.31 - 3.41) | 2.11  (9.83) | 1.81  (1.21) | 0.86  (0.19) |
| General disorders and administration site conditions | High-pitched crying | 23 | 3.36  (2.01 - 5.63) | 3.36  (23.97) | 2.48  (1.61) | 1.31  (0.62) |
| General disorders and administration site conditions | Injection site extravasation | 23 | 2.79  (1.69 - 4.59) | 2.79  (17.70) | 2.20  (1.45) | 1.14  (0.46) |
| Skin and subcutaneous tissue disorders | Skin disorder | 22 | 2.67  (1.61 - 4.42) | 2.67  (15.60) | 2.13  (1.40) | 1.09  (0.40) |
| Respiratory, thoracic and mediastinal disorders | Grunting | 22 | 1.82  (1.12 - 2.94) | 1.82  (6.12) | 1.62  (1.08) | 0.69  (0.02) |
| Respiratory, thoracic and mediastinal disorders | Aspiration | 22 | 1.72  (1.07 - 2.77) | 1.72  (5.06) | 1.55  (1.04) | 0.63  (-0.04) |
| Skin and subcutaneous tissue disorders | Angioneurotic oedema | 21 | 3.32  (1.94 - 5.69) | 3.32  (21.53) | 2.47  (1.57) | 1.30  (0.58) |
| General disorders and administration site conditions | Extensive swelling of vaccinated limb | 21 | 1.93  (1.18 - 3.17) | 1.93  (7.02) | 1.69  (1.12) | 0.76  (0.07) |
| Psychiatric disorders | Confusional state | 19 | 1.87  (1.11 - 3.13) | 1.87  (5.75) | 1.65  (1.07) | 0.72  (0.00) |
| Congenital, familial and genetic disorders | Congenital anomaly | 19 | 2.25  (1.33 - 3.84) | 2.25  (9.50) | 1.90  (1.22) | 0.92  (0.19) |
| General disorders and administration site conditions | Feeling cold | 19 | 2.04  (1.21 - 3.45) | 2.04  (7.43) | 1.77  (1.14) | 0.82  (0.09) |
| Blood and lymphatic system disorders | White blood cell disorder | 19 | 2.52  (1.47 - 4.32) | 2.52  (12.04) | 2.05  (1.31) | 1.04  (0.3) |
| Investigations | Oxygen saturation normal | 19 | 2.30  (1.35 - 3.92) | 2.30  (9.97) | 1.93  (1.23) | 0.95  (0.21) |
| Cardiac disorders | Cardiovascular disorder | 18 | 1.77  (1.04 – 3.00) | 1.77  (4.58) | 1.59  (1.02) | 0.67  (-0.07) |
| Blood and lymphatic system disorders | Haemolytic anaemia | 18 | 2.14  (1.24 - 3.67) | 2.14  (7.91) | 1.83  (1.16) | 0.87  (0.12) |
| General disorders and administration site conditions | Influenza like illness | 18 | 1.97  (1.15 - 3.37) | 1.97  (6.4) | 1.72  (1.1) | 0.78  (0.04) |
| Injury, poisoning and procedural complications | Metal poisoning | 18 | 2.44  (1.41 - 4.24) | 2.44  (10.72) | 2.01  (1.27) | 1.01  (0.25) |
| Infections and infestations | Pertussis | 18 | 2.7  (1.54 - 4.73) | 2.7  (13.05) | 2.15  (1.35) | 1.11  (0.34) |
| Surgical and medical procedures | Oxygen supplementation | 18 | 3.42  (1.91 - 6.13) | 3.42  (19.24) | 2.51  (1.54) | 1.33  (0.55) |
| Eye disorders | Retinal haemorrhage | 17 | 2.69  (1.51 - 4.79) | 2.69  (12.26) | 2.15  (1.33) | 1.10  (0.32) |
| Infections and infestations | Staphylococcal infection | 17 | 2.11  (1.21 - 3.67) | 2.10  (7.20) | 1.81  (1.13) | 0.85  (0.08) |
| Psychiatric disorders | Head banging | 17 | 2.93  (1.63 - 5.27) | 2.93  (14.30) | 2.28  (1.39) | 1.19  (0.40) |
| Musculoskeletal and connective tissue disorders | Growth retardation | 16 | 1.82  (1.04 - 3.20) | 1.82  (4.50) | 1.62  (1.01) | 0.70  (-0.08) |
| General disorders and administration site conditions | Similar reaction on previous exposure to drug | 16 | 2.85  (1.56 - 5.19) | 2.85  (12.79) | 2.23  (1.35) | 1.16  (0.35) |
| Musculoskeletal and connective tissue disorders | Torticollis | 16 | 3.51  (1.88 - 6.54) | 3.5  (17.73) | 2.55  (1.51) | 1.35  (0.52) |
| Investigations | Scan brain | 16 | 1.94  (1.10 - 3.42) | 1.94  (5.43) | 1.70  (1.06) | 0.77  (-0.02) |
| Investigations | Virus serology test | 15 | 2.25  (1.24 - 4.09) | 2.25  (7.45) | 1.89  (1.15) | 0.92  (0.10) |
| Ear and labyrinth disorders | Hyperacusis | 14 | 2.75  (1.45 - 5.20) | 2.75  (10.51) | 2.18  (1.28) | 1.12  (0.26) |
| Psychiatric disorders | Attention deficit/hyperactivity disorder | 14 | 2.35  (1.26 - 4.37) | 2.35  (7.65) | 1.95  (1.16) | 0.97  (0.11) |
| General disorders and administration site conditions | Injection site vesicles | 14 | 2.49  (1.33 - 4.67) | 2.49  (8.70) | 2.04  (1.20) | 1.03  (0.17) |
| Infections and infestations | Oral candidiasis | 13 | 2.31  (1.21 - 4.41) | 2.31  (6.90) | 1.93  (1.13) | 0.95  (0.07) |
| Blood and lymphatic system disorders | Splenomegaly | 13 | 1.95  (1.04 - 3.66) | 1.95  (4.47) | 1.71  (1.01) | 0.77  (-0.10) |
| Ear and labyrinth disorders | Hearing impaired | 13 | 2.55  (1.33 - 4.91) | 2.55  (8.48) | 2.07  (1.20) | 1.05  (0.16) |
| Blood and lymphatic system disorders | Red blood cell abnormality | 12 | 2.85  (1.42 - 5.70) | 2.85  (9.59) | 2.23  (1.25) | 1.16  (0.23) |
| Nervous system disorders | Hyperkinesia | 12 | 2.01  (1.04 - 3.88) | 2.01  (4.50) | 1.75  (1.01) | 0.80  (-0.10) |
| Psychiatric disorders | Feeding disorder neonatal | 12 | 2.01  (1.04 - 3.88) | 2.01  (4.50) | 1.75  (1.01) | 0.80  (-0.1) |
| Respiratory, thoracic and mediastinal disorders | Respiratory depression | 12 | 4.56  (2.13 - 9.74) | 4.56  (18.51) | 2.98  (1.58) | 1.57  (0.60) |
| General disorders and administration site conditions | Injection site urticaria | 12 | 2.85  (1.42 - 5.70) | 2.85  (9.59) | 2.23  (1.25) | 1.16  (0.23) |
| Psychiatric disorders | Disturbance in social behaviour | 12 | 3.11  (1.54 - 6.28) | 3.11  (11.09) | 2.36  (1.31) | 1.24  (0.30) |
| Investigations | Brain scan normal | 12 | 2.28  (1.17 - 4.45) | 2.28  (6.15) | 1.91  (1.09) | 0.94  (0.02) |
| General disorders and administration site conditions | Cyst | 11 | 3.69  (1.73 - 7.87) | 3.69  (13.07) | 2.63  (1.39) | 1.40  (0.40) |
| Eye disorders | Eyelid ptosis | 11 | 2.02  (1.02 - 4.02) | 2.02  (4.19) | 1.75  (0.99) | 0.81  (-0.13) |
| Nervous system disorders | Haemorrhage intracranial | 11 | 2.09  (1.05 - 4.17) | 2.09  (4.56) | 1.80  (1.01) | 0.84  (-0.10) |
| Gastrointestinal disorders | Dyspepsia | 11 | 2.02  (1.02 - 4.02) | 2.02  (4.19) | 1.75  (0.99) | 0.81  (-0.13) |
| Psychiatric disorders | Emotional disorder | 11 | 2.41  (1.19 - 4.88) | 2.41  (6.37) | 1.99  (1.10) | 0.99  (0.04) |
| General disorders and administration site conditions | Multi-organ failure | 11 | 2.51  (1.23 - 5.09) | 2.51  (6.91) | 2.05  (1.13) | 1.03  (0.07) |
| Psychiatric disorders | Tension | 11 | 3.69  (1.73 - 7.87) | 3.69  (13.07) | 2.63  (1.39) | 1.40  (0.40) |
| Nervous system disorders | Syncope vasovagal | 11 | 6.96  (2.88 - 16.8) | 6.96  (25.27) | 3.68  (1.76) | 1.88  (0.84) |
| Metabolism and nutrition disorders | Food aversion | 11 | 2.41  (1.19 - 4.88) | 2.41  (6.37) | 1.99  (1.10) | 0.99  (0.04) |
| Nervous system disorders | Cerebral ischaemia | 10 | 2.11  (1.02 - 4.36) | 2.11  (4.26) | 1.81  (0.99) | 0.86  (-0.13) |
| Eye disorders | Eye oedema | 10 | 5.18  (2.2 - 12.19) | 5.18  (17.66) | 3.19  (1.56) | 1.67  (0.61) |
| Ear and labyrinth disorders | Hypoacusis | 10 | 2.28  (1.09 - 4.74) | 2.28  (5.12) | 1.91  (1.04) | 0.94  (-0.06) |
| Investigations | Blood pressure | 10 | 4.75  (2.05 - 10.99) | 4.75  (16.13) | 3.04  (1.51) | 1.61  (0.55) |
| Skin and subcutaneous tissue disorders | Skin nodule | 9 | 2.85  (1.28 - 6.34) | 2.85  (7.19) | 2.23  (1.14) | 1.16  (0.10) |
| Psychiatric disorders | Depression | 9 | 2.56  (1.17 - 5.63) | 2.56  (5.92) | 2.08  (1.08) | 1.06  (0.00) |
| Psychiatric disorders | Abnormal sleep-related event | 9 | 2.56  (1.17 - 5.63) | 2.56  (5.92) | 2.08  (1.08) | 1.06  (0.00) |
| Musculoskeletal and connective tissue disorders | Muscle disorder | 9 | 3.02  (1.34 - 6.77) | 3.02  (7.93) | 2.32  (1.18) | 1.21  (0.14) |
| Musculoskeletal and connective tissue disorders | Toe walking | 9 | 2.70  (1.22 - 5.96) | 2.70  (6.53) | 2.15  (1.11) | 1.11  (0.05) |
| Nervous system disorders | Sensory processing disorder | 9 | 3.66  (1.58 - 8.46) | 3.66  (10.6) | 2.62  (1.30) | 1.39  (0.30) |
| Surgical and medical procedures | Ear tube insertion | 9 | 2.70  (1.22 - 5.96) | 2.70  (6.53) | 2.15  (1.11) | 1.11  (0.05) |
| Congenital, familial and genetic disorders | Chromosome abnormality | 8 | 5.70  (2.14 - 15.18) | 5.70  (15.48) | 3.35  (1.47) | 1.74  (0.55) |
| General disorders and administration site conditions | Mucous membrane disorder | 8 | 2.40  (1.05 - 5.48) | 2.40  (4.59) | 1.98  (0.99) | 0.99  (-0.12) |
| Surgical and medical procedures | Vasodilation procedure | 8 | 22.78  (4.84 - 107.3) | 22.78  (33.32) | 5.36  (1.46) | 2.42  (1.12) |
| Neoplasms benign, malignant and unspecified (incl cysts and polyps) | Haemangioma | 8 | 3.80  (1.55 - 9.29) | 3.80  (9.89) | 2.68  (1.27) | 1.42  (0.27) |
| Blood and lymphatic system disorders | Lymph node pain | 8 | 2.68  (1.16 - 6.21) | 2.68  (5.73) | 2.14  (1.06) | 1.10  (-0.02) |
| Investigations | Heart rate normal | 8 | 2.40  (1.05 - 5.48) | 2.40  (4.59) | 1.98  (0.99) | 0.99  (-0.12) |
| Investigations | Blood pressure increased | 7 | 2.49  (1.03 - 6.06) | 2.49  (4.35) | 2.04  (0.97) | 1.03  (-0.15) |
| General disorders and administration site conditions | Chest discomfort | 7 | 2.66  (1.08 - 6.52) | 2.66  (4.94) | 2.13  (1.01) | 1.09  (-0.09) |
| Respiratory, thoracic and mediastinal disorders | Intercostal retraction | 7 | 3.99  (1.52 - 10.48) | 3.99  (9.21) | 2.76  (1.23) | 1.46  (0.24) |
| Nervous system disorders | Exaggerated startle response | 7 | 2.66  (1.08 - 6.52) | 2.66  (4.94) | 2.13  (1.01) | 1.09  (-0.09) |
| Investigations | Drug screen | 6 | 2.63  (1 - 6.92) | 2.63  (4.14) | 2.11  (0.94) | 1.08  (-0.19) |
| Injury, poisoning and procedural complications | Overdose | 6 | 2.85  (1.07 - 7.59) | 2.85  (4.80) | 2.23  (0.98) | 1.16  (-0.12) |
| Skin and subcutaneous tissue disorders | Skin irritation | 6 | 8.54  (2.41 - 30.28) | 8.54  (15.98) | 4.02  (1.39) | 2.01  (0.61) |
| Investigations | Acoustic stimulation tests | 6 | 3.42  (1.24 - 9.4) | 3.42  (6.41) | 2.51  (1.08) | 1.33  (0.03) |
| Investigations | Blood aluminium increased | 6 | 3.42  (1.24 - 9.4) | 3.42  (6.41) | 2.51  (1.08) | 1.33  (0.03) |
| Injury, poisoning and procedural complications | Reaction to previous exposure to any vaccine | 6 | 3.42  (1.24 - 9.4) | 3.42  (6.41) | 2.51  (1.08) | 1.33  (0.03) |
| Respiratory, thoracic and mediastinal disorders | Cyanosis central | 6 | 3.42  (1.24 - 9.4) | 3.42  (6.41) | 2.51  (1.08) | 1.33  (0.03) |
| Psychiatric disorders | Hostility | 5 | 3.16  (1.06 - 9.44) | 3.16  (4.76) | 2.39  (0.96) | 1.26  (-0.14) |
| Metabolism and nutrition disorders | Hyperchloraemia | 5 | 4.75  (1.45 - 15.55) | 4.75  (8.06) | 3.04  (1.13) | 1.61  (0.16) |
| Injury, poisoning and procedural complications | Nerve injury | 5 | 2.85  (0.97 - 8.33) | 2.85  (4.00) | 2.23  (0.91) | 1.16  (-0.22) |
| Injury, poisoning and procedural complications | Fracture | 5 | 5.70  (1.65 - 19.68) | 5.7  (9.68) | 3.35  (1.19) | 1.74  (0.28) |
| Vascular disorders | Lymphoedema | 5 | 5.70  (1.65 - 19.68) | 5.70  (9.68) | 3.35  (1.19) | 1.74  (0.28) |
| Investigations | Virus culture | 5 | 7.12  (1.91 - 26.51) | 7.12  (11.69) | 3.72  (1.24) | 1.90  (0.41) |
| Skin and subcutaneous tissue disorders | Skin tightness | 5 | 28.48  (3.33 - 243.78) | 28.48  (22.09) | 5.58  (0.93) | 2.48  (0.87) |
| Investigations | Weight | 5 | 3.16  (1.06 - 9.44) | 3.16  (4.76) | 2.39  (0.96) | 1.26  (-0.14) |
| Investigations | Head circumference normal | 5 | 5.70  (1.65 - 19.68) | 5.70  (9.68) | 3.35  (1.19) | 1.74  (0.28) |
| Infections and infestations | Conjunctivitis infective | 5 | 7.12  (1.91 - 26.51) | 7.12  (11.69) | 3.72  (1.24) | 1.90  (0.41) |
| Endocrine disorders | Adrenal disorder | 4 | 11.39  (2.09 - 62.2) | 11.39  (12.64) | 4.46  (1.08) | 2.16  (0.47) |
| Eye disorders | Pupillary disorder | 4 | 3.80  (1.07 - 13.46) | 3.80  (4.94) | 2.68  (0.93) | 1.42  (-0.14) |
| Pregnancy, puerperium and perinatal conditions | Neonatal disorder | 4 | 4.56  (1.22 - 16.97) | 4.56  (6.17) | 2.98  (0.99) | 1.57  (-0.01) |
| Eye disorders | Exophthalmos | 4 | 5.70  (1.42 - 22.77) | 5.70  (7.74) | 3.35  (1.05) | 1.74  (0.13) |
| Psychiatric disorders | Self injurious behaviour | 4 | 4.56  (1.22 - 16.97) | 4.56  (6.17) | 2.98  (0.99) | 1.57  (-0.01) |
| Respiratory, thoracic and mediastinal disorders | Suffocation feeling | 4 | 5.70  (1.42 - 22.77) | 5.70  (7.74) | 3.35  (1.05) | 1.74  (0.13) |
| Respiratory, thoracic and mediastinal disorders | Anoxia | 4 | 11.39  (2.09 - 62.2) | 11.39  (12.64) | 4.46  (1.08) | 2.16  (0.47) |
| Vascular disorders | Infarction | 4 | 4.56  (1.22 - 16.97) | 4.56  (6.17) | 2.98  (0.99) | 1.57  (-0.01) |
| Respiratory, thoracic and mediastinal disorders | Bronchitis chronic | 4 | 3.80  (1.07 - 13.46) | 3.80  (4.94) | 2.68  (0.93) | 1.42  (-0.14) |
| Psychiatric disorders | Asperger's disorder | 4 | 5.70  (1.42 - 22.77) | 5.70  (7.74) | 3.35  (1.05) | 1.74  (0.13) |
| Nervous system disorders | Leukoencephalomyelitis | 4 | 5.70  (1.42 - 22.77) | 5.70  (7.74) | 3.35  (1.05) | 1.74  (0.13) |
| Investigations | Clostridium difficile toxin test | 4 | 3.80  (1.07 - 13.46) | 3.80  (4.94) | 2.68  (0.93) | 1.42  (-0.14) |
| Investigations | Laparoscopy | 4 | 5.70  (1.42 - 22.77) | 5.70  (7.74) | 3.35  (1.05) | 1.74  (0.13) |
| Investigations | Pertussis identification test positive | 4 | 22.78  (2.55 - 203.85) | 22.78  (16.66) | 5.36  (0.86) | 2.42  (0.67) |
| Infections and infestations | Acute sinusitis | 4 | 5.70  (1.42 - 22.77) | 5.70  (7.74) | 3.35  (1.05) | 1.74  (0.13) |
| Ear and labyrinth disorders | Ear haemorrhage | 4 | 11.39  (2.09 - 62.2) | 11.39  (12.64) | 4.46  (1.08) | 2.16  (0.47) |
| Investigations | Blood heavy metal test | 4 | 7.59  (1.70 - 33.93) | 7.59  (9.81) | 3.83  (1.09) | 1.94  (0.29) |
| Investigations | Anticonvulsant drug level therapeutic | 3 | 4.27  (0.96 - 19.09) | 4.27  (4.3) | 2.87  (0.82) | 1.52  (-0.25) |
| Congenital, familial and genetic disorders | Tourette's disorder | 3 | 17.09  (1.78 - 164.27) | 17.09  (11.36) | 5.02  (0.76) | 2.33  (0.39) |
| Gastrointestinal disorders | Glossitis | 3 | 17.09  (1.78 - 164.27) | 17.09  (11.36) | 5.02  (0.76) | 2.33  (0.39) |
| Investigations | Red blood cell sedimentation rate decreased | 3 | 17.09  (1.78 - 164.27) | 17.09  (11.36) | 5.02  (0.76) | 2.33  (0.39) |
| Gastrointestinal disorders | Irritable bowel syndrome | 3 | 5.70  (1.15 - 28.22) | 5.70  (5.81) | 3.35  (0.88) | 1.74  (-0.07) |
| Cardiac disorders | Bundle branch block | 3 | 5.70  (1.15 - 28.22) | 5.70  (5.81) | 3.35  (0.88) | 1.74  (-0.07) |
| Respiratory, thoracic and mediastinal disorders | Pulmonary embolism | 3 | 8.54  (1.43 - 51.13) | 8.54  (7.99) | 4.02  (0.90) | 2.01  (0.14) |
| Investigations | Cystogram | 3 | 4.27  (0.96 - 19.09) | 4.27  (4.3) | 2.87  (0.82) | 1.52  (-0.25) |
| Investigations | Bacterial culture | 3 | 17.09  (1.78 - 164.27) | 17.09  (11.36) | 5.02  (0.76) | 2.33  (0.39) |
| Psychiatric disorders | Asocial behaviour | 3 | 5.70  (1.15 - 28.22) | 5.70  (5.81) | 3.35  (0.88) | 1.74  (-0.07) |
| Investigations | Blood urea nitrogen/creatinine ratio increased | 3 | 4.27  (0.96 - 19.09) | 4.27  (4.3) | 2.87  (0.82) | 1.52  (-0.25) |
| Investigations | Carbon dioxide abnormal | 3 | 8.54  (1.43 - 51.13) | 8.54  (7.99) | 4.02  (0.9) | 2.01  (0.14) |
| Psychiatric disorders | Emotional poverty | 3 | 4.27  (0.96 - 19.09) | 4.27  (4.3) | 2.87  (0.82) | 1.52  (-0.25) |
| Investigations | Cytomegalovirus antibody negative | 3 | 4.27  (0.96 - 19.09) | 4.27  (4.30) | 2.87  (0.82) | 1.52  (-0.25) |
| Blood and lymphatic system disorders | Aplasia pure red cell | 3 | 8.54  (1.43 - 51.13) | 8.54  (7.99) | 4.02  (0.90) | 2.01  (0.14) |
| Gastrointestinal disorders | Peristalsis visible | 3 | 4.27  (0.96 - 19.09) | 4.27  (4.30) | 2.87  (0.82) | 1.52  (-0.25) |
| Gastrointestinal disorders | Sandifer's syndrome | 3 | 4.27  (0.96 - 19.09) | 4.27  (4.30) | 2.87  (0.82) | 1.52  (-0.25) |
| Investigations | Respiratory syncytial virus serology | 3 | 17.09  (1.78 - 164.27) | 17.09  (11.36) | 5.02  (0.76) | 2.33  (0.39) |
| Investigations | Body height | 3 | 4.27  (0.96 - 19.09) | 4.27  (4.30) | 2.87  (0.82) | 1.52  (-0.25) |
| Social circumstances | Hearing aid user | 3 | 8.54  (1.43 - 51.13) | 8.54  (7.99) | 4.02  (0.90) | 2.01  (0.14) |
| General disorders and administration site conditions | Injection site lymphadenopathy | 3 | 17.09  (1.78 - 164.27) | 17.09  (11.36) | 5.02  (0.76) | 2.33  (0.39) |

DTaP: diphtheria, tetanus, and acellular pertussis; SAE serious adverse event; PT, preferred term; SOC, System Organ Class; CI, confidence interval; ROR, reporting odds ratio; PRR, proportional reporting ratio; χ^2^, Chi-squared; IC, information component; IC-2SD, the lower limit of the 95% two-sided CI of the IC; EBGM, empirical Bayesian geometric mean; EBGM05, the lower 95 two-sided CI of EBGM.

A: SAEs B: Fatalities


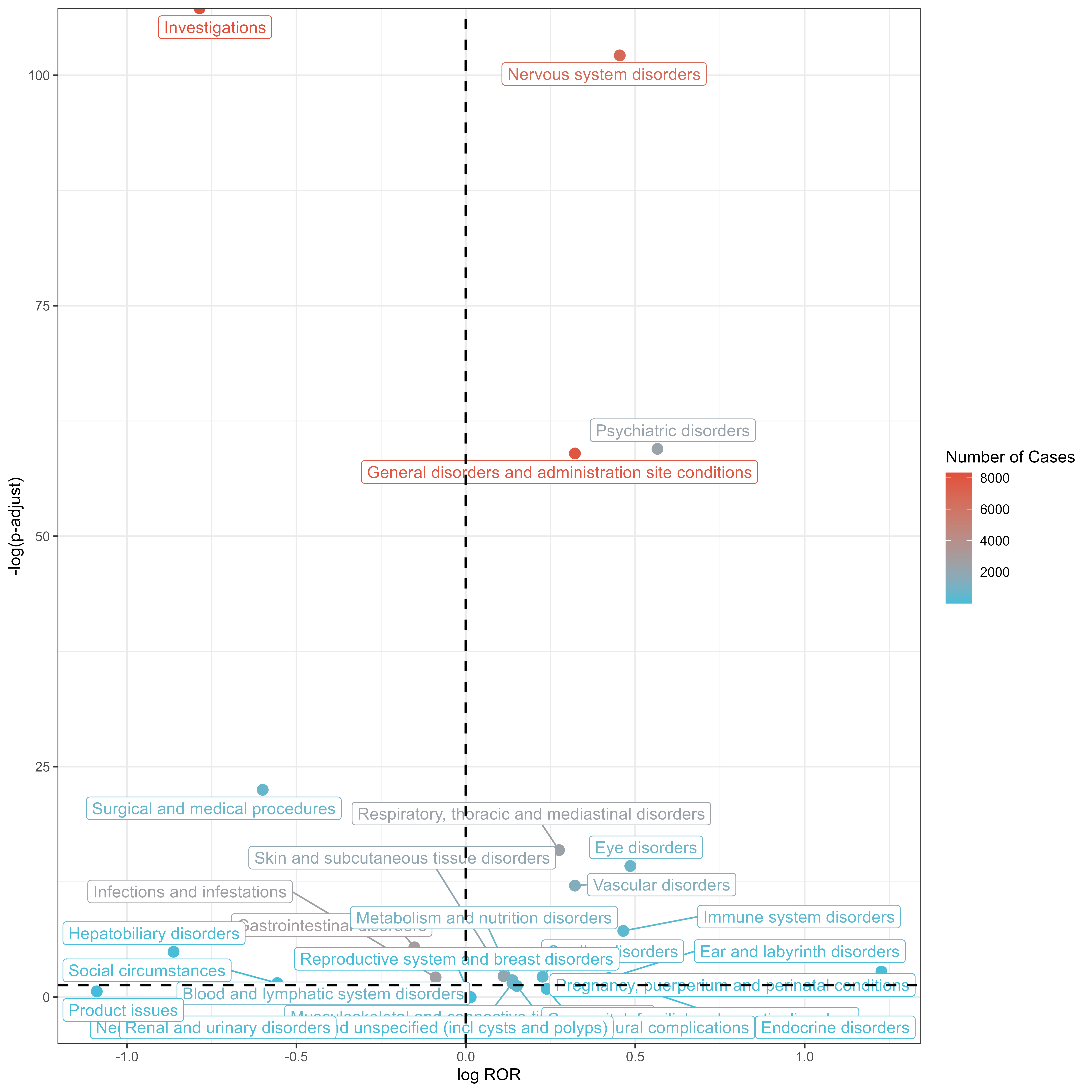

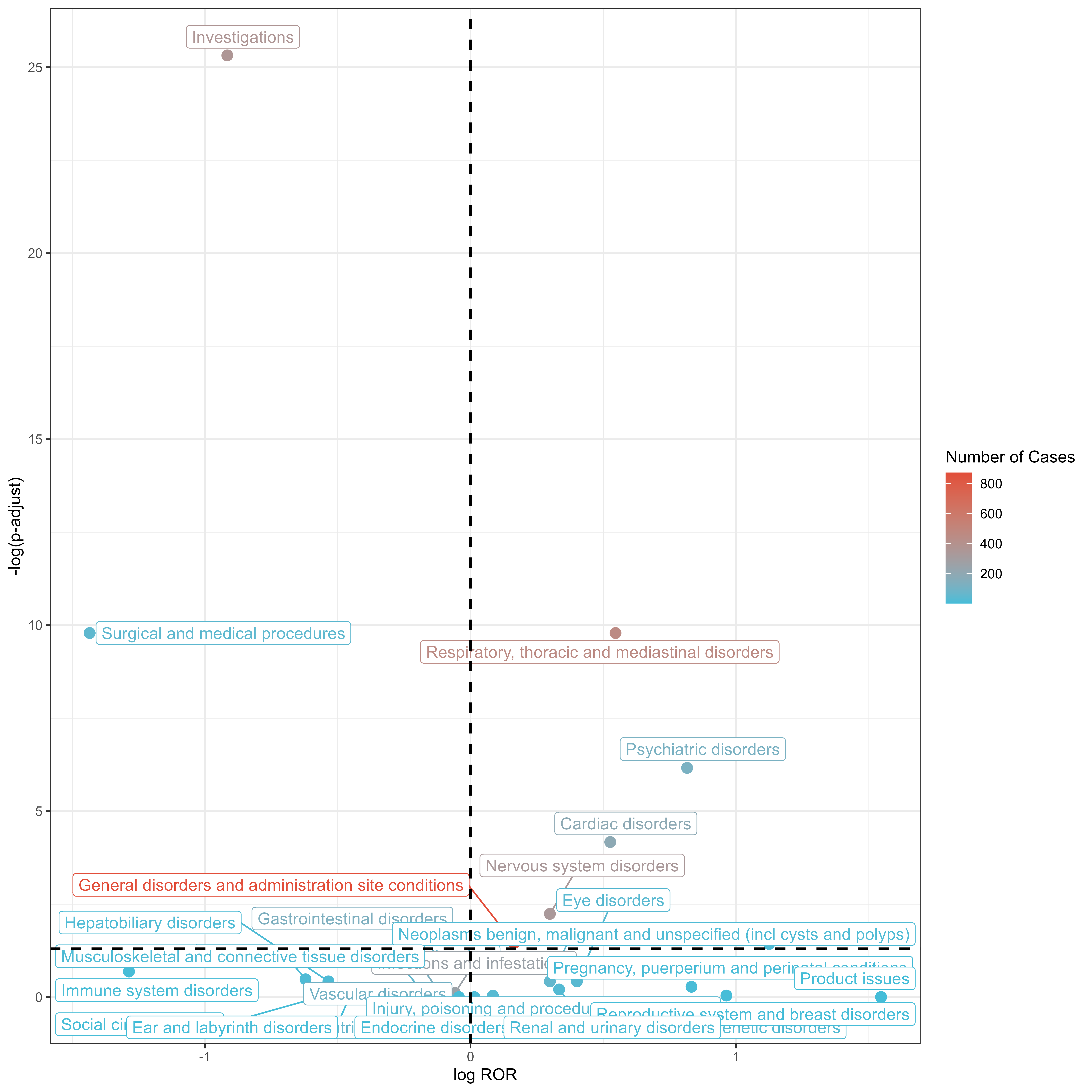


# Figure S3. Volcano plot of SOCs corresponding to reported SAEs and fatalities.

SOC, system organ classes; SAEs, serious adverse events; ROR, reporting odds ratio.

# Table S6. Signal strength of SAE reports of DTaP vaccines at the SOC level.

| SOC | a | ROR  (95%Cl) | PRR  (χ^2^) | EBGM  (EBGM05) | IC  (IC-2SD) |
| --- | --- | --- | --- | --- | --- |
| Investigations | 8321 | 0.58  (0.57 - 0.60) | 0.66  (1769.90) | 0.70  (0.68) | -0.52  (-0.55) |
| General disorders and administration site conditions | 7875 | 1.25  (1.22 - 1.29) | 1.21  (269.67) | 1.17  (1.14) | 0.23  (0.19) |
| Nervous system disorders | 6767 | 1.37  (1.33 - 1.41) | 1.31  (469.46) | 1.25  (1.22) | 0.33  (0.29) |
| Gastrointestinal disorders | 2885 | 0.90  (0.87 - 0.94) | 0.91  (23.36) | 0.92  (0.89) | -0.12  (-0.17) |
| Infections and infestations | 2643 | 0.94  (0.9 - 0.98) | 0.94  (8.41) | 0.95  (0.92) | -0.07  (-0.13) |
| Respiratory, thoracic and mediastinal disorders | 2468 | 1.21  (1.16 - 1.27) | 1.20  (71.97) | 1.17  (1.12) | 0.22  (0.16) |
| Psychiatric disorders | 2339 | 1.48  (1.41 - 1.55) | 1.45  (272.72) | 1.36  (1.31) | 0.44  (0.38) |
| Skin and subcutaneous tissue disorders | 2045 | 1.08  (1.03 - 1.13) | 1.07  (9.24) | 1.06  (1.02) | 0.09  (0.02) |
| Vascular disorders | 1398 | 1.25  (1.17 - 1.32) | 1.24  (53.96) | 1.20  (1.14) | 0.26  (0.17) |
| Metabolism and nutrition disorders | 948 | 1.10  (1.02 - 1.18) | 1.10  (6.92) | 1.08  (1.02) | 0.11  (0.01) |
| Musculoskeletal and connective tissue disorders | 775 | 1.10  (1.02 - 1.19) | 1.10  (5.47) | 1.08  (1.01) | 0.11  (0.00) |
| Blood and lymphatic system disorders | 724 | 0.93  (0.86 - 1.01) | 0.94  (2.77) | 0.95  (0.88) | -0.08  (-0.20) |
| Eye disorders | 721 | 1.40  (1.29 - 1.52) | 1.39  (64.00) | 1.31  (1.23) | 0.39  (0.27) |
| Surgical and medical procedures | 655 | 0.66  (0.61 - 0.71) | 0.66  (102.27) | 0.70  (0.65) | -0.52  (-0.64) |
| Injury, poisoning and procedural complications | 453 | 1.11  (1.00 - 1.23) | 1.11  (4.15) | 1.09  (1.00) | 0.13  (-0.02) |
| Cardiac disorders | 446 | 1.17  (1.06 - 1.30) | 1.17  (8.96) | 1.14  (1.05) | 0.19  (0.04) |
| Immune system disorders | 386 | 1.38  (1.23 - 1.54) | 1.37  (31.75) | 1.30  (1.18) | 0.38  (0.22) |
| Renal and urinary disorders | 129 | 0.89  (0.74 - 1.07) | 0.89  (1.51) | 0.91  (0.77) | -0.14  (-0.41) |
| Congenital, familial and genetic disorders | 125 | 1.18  (0.97 - 1.43) | 1.18  (2.87) | 1.15  (0.98) | 0.20  (-0.08) |
| Ear and labyrinth disorders | 121 | 1.34  (1.1 - 1.63) | 1.34  (8.28) | 1.27  (1.08) | 0.35  (0.06) |
| Hepatobiliary disorders | 62 | 0.55  (0.42 - 0.71) | 0.55  (21.45) | 0.59  (0.47) | -0.77  (-1.15) |
| Social circumstances | 44 | 0.68  (0.50 - 0.93) | 0.68  (5.80) | 0.72  (0.55) | -0.48  (-0.93) |
| Neoplasms benign, malignant and unspecified (incl cysts and polyps) | 42 | 1.00  (0.72 - 1.39) | 1.00  (0.00) | 1.00  (0.76) | 0.00  (-0.47) |
| Pregnancy, puerperium and perinatal conditions | 23 | 2.34  (1.44 - 3.80) | 2.34  (12.50) | 1.95  (1.30) | 0.96  (0.29) |
| Endocrine disorders | 23 | 1.44  (0.91 - 2.27) | 1.44  (2.46) | 1.35  (0.92) | 0.43  (-0.21) |
| Reproductive system and breast disorders | 15 | 1.01  (0.58 - 1.74) | 1.01  (0.00) | 1.00  (0.63) | 0.01  (-0.77) |
| Product issues | 4 | 0.47  (0.17 - 1.32) | 0.47  (2.15) | 0.52  (0.22) | -0.96  (-2.31) |

SAE, serious adverse event; DTaP: diphtheria, tetanus, and acellular pertussis; CI, confidence interval; SOC, System Organ Class; ROR, reporting odds ratio; PRR, proportional reporting ratio; χ^2^, Chi-squared; IC, information component; IC-2SD, the lower limit of the 95% two-sided CI of the IC; EBGM, empirical Bayesian geometric mean; EBGM05, the lower 95 two-sided CI of EBGM.

# Table S7. Signal strength of DTaP-related fatal SAE positive PT signals and their corresponding SOCs.

| SOC | PT | a | ROR (95%Cl) | PRR (χ^2^) | EBGM (EBGM05) | IC (IC-2SD) |
| --- | --- | --- | --- | --- | --- | --- |
| General disorders and administration site conditions | Sudden infant death syndrome | 329 | 1.97  (1.73 - 2.24) | 1.87  (107.38) | 1.66  (1.49) | 0.73  (0.55) |
| Respiratory, thoracic and mediastinal disorders | Apnoea | 118 | 2.25  (1.81 - 2.79) | 2.20  (57.49) | 1.87  (1.57) | 0.91  (0.61) |
| General disorders and administration site conditions | Unevaluable event | 95 | 3.42  (2.65 - 4.41) | 3.35  (100.57) | 2.49  (2.01) | 1.32  (0.97) |
| Cardiac disorders | Cardiac arrest | 94 | 1.62  (1.29 - 2.04) | 1.60  (17.04) | 1.47  (1.21) | 0.56  (0.23) |
| Nervous system disorders | Coma | 69 | 5.01  (3.63 - 6.91) | 4.92  (117.82) | 3.13  (2.39) | 1.65  (1.23) |
| Vascular disorders | Cyanosis | 56 | 1.65  (1.22 - 2.22) | 1.64  (10.97) | 1.50  (1.17) | 0.58  (0.16) |
| Psychiatric disorders | Irritability | 44 | 3.13  (2.17 - 4.52) | 3.10  (41.13) | 2.37  (1.74) | 1.25  (0.74) |
| Respiratory, thoracic and mediastinal disorders | Pulmonary oedema | 43 | 2.09  (1.47 - 2.97) | 2.08  (17.87) | 1.80  (1.34) | 0.84  (0.35) |
| Respiratory, thoracic and mediastinal disorders | Pulmonary congestion | 33 | 9.74  (5.58 - 16.99) | 9.65  (96.75) | 4.26  (2.68) | 2.09  (1.46) |
| Psychiatric disorders | Agitation | 31 | 1.68  (1.13 - 2.52) | 1.68  (6.64) | 1.53  (1.09) | 0.61  (0.04) |
| Respiratory, thoracic and mediastinal disorders | Asphyxia | 26 | 2.12  (1.35 - 3.33) | 2.11  (11.22) | 1.82  (1.25) | 0.86  (0.23) |
| Investigations | Laboratory test abnormal | 24 | 2.04  (1.28 - 3.25) | 2.03  (9.39) | 1.77  (1.20) | 0.82  (0.17) |
| Infections and infestations | Bacterial infection | 21 | 2.28  (1.38 - 3.78) | 2.27  (10.82) | 1.92  (1.26) | 0.94  (0.24) |
| Respiratory, thoracic and mediastinal disorders | Nasal congestion | 18 | 6.61  (3.37 - 12.97) | 6.58  (40.12) | 3.62  (2.06) | 1.86  (1.03) |
| Metabolism and nutrition disorders | Anorexia | 14 | 1.86  (1.02 - 3.41) | 1.86  (4.24) | 1.65  (1.00) | 0.72  (-0.11) |
| Investigations | White blood cell count increased | 12 | 2.01  (1.04 - 3.87) | 2.00  (4.51) | 1.75  (1.01) | 0.81  (-0.10) |
| Injury, poisoning and procedural complications | Injury | 11 | 2.80  (1.36 - 5.75) | 2.80  (8.6) | 2.22  (1.21) | 1.15  (0.18) |
| Cardiac disorders | Myocarditis | 11 | 2.15  (1.08 - 4.29) | 2.14  (4.92) | 1.84  (1.03) | 0.88  (-0.07) |
| Infections and infestations | Upper respiratory tract infection | 10 | 2.44  (1.17 - 5.11) | 2.44  (5.98) | 2.01  (1.09) | 1.01  (0.01) |
| Cardiac disorders | Cardiovascular disorder | 9 | 2.29  (1.06 - 4.96) | 2.29  (4.7) | 1.93  (1.01) | 0.95  (-0.1) |
| Psychiatric disorders | Abnormal sleep-related event | 9 | 2.93  (1.31 - 6.52) | 2.92  (7.60) | 2.28  (1.17) | 1.19  (0.13) |
| Respiratory, thoracic and mediastinal disorders | Pneumonitis | 8 | 3.60  (1.49 - 8.70) | 3.60  (9.30) | 2.61  (1.25) | 1.38  (0.24) |
| Metabolism and nutrition disorders | Oral intake reduced | 8 | 3.90  (1.59 - 9.56) | 3.90  (10.35) | 2.74  (1.29) | 1.45  (0.30) |
| General disorders and administration site conditions | Multi-organ failure | 8 | 2.60  (1.13 - 5.99) | 2.60  (5.45) | 2.11  (1.05) | 1.07  (-0.04) |
| Gastrointestinal disorders | Gastrointestinal haemorrhage | 6 | 3.19  (1.18 - 8.64) | 3.19  (5.84) | 2.42  (1.05) | 1.27  (-0.01) |
| General disorders and administration site conditions | Influenza like illness | 6 | 35.14  (4.23 - 292.01) | 35.08  (28.39) | 5.87  (1.00) | 2.55  (1.06) |
| Blood and lymphatic system disorders | Splenomegaly | 5 | 3.66  (1.20 - 11.19) | 3.65  (5.93) | 2.63  (1.03) | 1.40  (-0.01) |
| Hepatobiliary disorders | Liver disorder | 5 | 4.18  (1.33 - 13.18) | 4.18  (7.05) | 2.85  (1.09) | 1.51  (0.09) |
| Investigations | Heart rate increased | 5 | 3.66  (1.20 - 11.19) | 3.65  (5.93) | 2.63  (1.03) | 1.40  (-0.01) |
| Nervous system disorders | Cerebrovascular disorder | 4 | 4.68  (1.26 - 17.44) | 4.68  (6.43) | 3.04  (1.01) | 1.61  (0.02) |
| General disorders and administration site conditions | Injection site oedema | 4 | 3.90  (1.10 - 13.83) | 3.90  (5.17) | 2.74  (0.95) | 1.45  (-0.10) |
| Blood and lymphatic system disorders | Red blood cell abnormality | 4 | 11.71  (2.14 - 63.94) | 11.69  (13.04) | 4.56  (1.10) | 2.19  (0.50) |
| Renal and urinary disorders | Renal disorder | 4 | 7.8  (1.75 - 34.88) | 7.8  (10.16) | 3.91  (1.12) | 1.97  (0.32) |
| Respiratory, thoracic and mediastinal disorders | Grunting | 4 | 4.68  (1.26 - 17.44) | 4.68  (6.43) | 3.04  (1.01) | 1.61  (0.02) |
| Cardiac disorders | Cardiac disorder | 4 | 3.34  (0.98 - 11.43) | 3.34  (4.18) | 2.49  (0.89) | 1.32  (-0.22) |
| Psychiatric disorders | Staring | 4 | 3.34  (0.98 - 11.43) | 3.34  (4.18) | 2.49  (0.89) | 1.32  (-0.22) |
| Nervous system disorders | Subarachnoid haemorrhage | 4 | 3.34  (0.98 - 11.43) | 3.34  (4.18) | 2.49  (0.89) | 1.32  (-0.22) |
| Eye disorders | Eye movement disorder | 4 | 4.68  (1.26 - 17.44) | 4.68  (6.43) | 3.04  (1.01) | 1.61  (0.02) |
| Infections and infestations | Respiratory syncytial virus infection | 4 | 3.90  (1.10 - 13.83) | 3.90  (5.17) | 2.74  (0.95) | 1.45  (-0.10) |
| Infections and infestations | Pertussis | 4 | 11.71  (2.14 - 63.94) | 11.69  (13.04) | 4.56  (1.10) | 2.19  (0.50) |
| Infections and infestations | Oral candidiasis | 3 | 8.78  (1.47 - 52.55) | 8.77  (8.26) | 4.11  (0.92) | 2.04  (0.18) |
| Endocrine disorders | Adrenal disorder | 3 | 17.55  (1.83 - 168.82) | 17.54  (11.70) | 5.13  (0.77) | 2.36  (0.42) |
| Gastrointestinal disorders | Gastrooesophageal reflux disease | 3 | 4.39  (0.98 - 19.62) | 4.38  (4.48) | 2.93  (0.84) | 1.55  (-0.21) |
| Injury, poisoning and procedural complications | Fracture | 3 | 8.78  (1.47 - 52.55) | 8.77  (8.26) | 4.11  (0.92) | 2.04  (0.18) |
| Infections and infestations | Tracheitis | 3 | 5.85  (1.18 - 29) | 5.85  (6.03) | 3.42  (0.90) | 1.78  (-0.03) |
| Respiratory, thoracic and mediastinal disorders | Pulmonary embolism | 3 | 17.55  (1.83 - 168.82) | 17.54  (11.70) | 5.13  (0.77) | 2.36  (0.42) |
| Nervous system disorders | Epilepsy | 3 | 4.39  (0.98 - 19.62) | 4.38  (4.48) | 2.93  (0.84) | 1.55  (-0.21) |
| Blood and lymphatic system disorders | White blood cell disorder | 3 | 8.78  (1.47 - 52.55) | 8.77  (8.26) | 4.11  (0.92) | 2.04  (0.18) |
| Investigations | Red blood cell count decreased | 3 | 5.85  (1.18 – 29.00) | 5.85  (6.03) | 3.42  (0.90) | 1.78  (-0.03) |

DTaP, diphtheria, tetanus, and acellular pertussis; SAE serious adverse event; PT, preferred term; SOC, System Organ Class; CI, confidence interval; ROR, reporting odds ratio; PRR, proportional reporting ratio; χ^2^, Chi-squared; IC, information component; IC-2SD, the lower limit of the 95% two-sided CI of the IC; EBGM, empirical Bayesian geometric mean; EBGM05, the lower 95 two-sided CI of EBGM.

# Table S8. Signal strength of fatal reports of DTaP vaccines at the SOC level.

| SOC | a | ROR  (95%Cl) | PRR  (χ^2^) | EBGM  (EBGM05) | IC  (IC-2SD) |
| --- | --- | --- | --- | --- | --- |
| General disorders and administration site conditions | 872 | 1.12  (1.03 - 1.22) | 1.09  (6.92) | 1.07  (1.00) | 0.10  (-0.01) |
| Respiratory, thoracic and mediastinal disorders | 455 | 1.46  (1.31 - 1.63) | 1.39  (45.58) | 1.32  (1.20) | 0.40  (0.25) |
| Investigations | 360 | 0.53  (0.47 - 0.60) | 0.58  (118.48) | 0.62  (0.56) | -0.69  (-0.85) |
| Nervous system disorders | 332 | 1.23  (1.09 - 1.39) | 1.21  (10.58) | 1.17  (1.05) | 0.23  (0.05) |
| Infections and infestations | 254 | 0.96  (0.83 - 1.10) | 0.96  (0.41) | 0.97  (0.86) | -0.05  (-0.25) |
| Cardiac disorders | 190 | 1.44  (1.22 - 1.7) | 1.42  (19.49) | 1.33  (1.16) | 0.42  (0.18) |
| Gastrointestinal disorders | 134 | 0.92  (0.76 - 1.11) | 0.92  (0.78) | 0.93  (0.80) | -0.10  (-0.37) |
| Psychiatric disorders | 119 | 1.76  (1.43 - 2.17) | 1.73  (29.01) | 1.56  (1.31) | 0.65  (0.35) |
| Vascular disorders | 103 | 0.93  (0.75 - 1.14) | 0.93  (0.52) | 0.94  (0.79) | -0.09  (-0.40) |
| Metabolism and nutrition disorders | 61 | 0.94  (0.72 - 1.24) | 0.94  (0.18) | 0.95  (0.76) | -0.07  (-0.47) |
| Blood and lymphatic system disorders | 58 | 1.23  (0.92 - 1.63) | 1.22  (2.00) | 1.19  (0.93) | 0.25  (-0.16) |
| Skin and subcutaneous tissue disorders | 57 | 0.88  (0.67 - 1.17) | 0.88  (0.77) | 0.90  (0.71) | -0.15  (-0.56) |
| Surgical and medical procedures | 46 | 0.37  (0.27 - 0.50) | 0.38  (46.17) | 0.41  (0.32) | -1.27  (-1.71) |
| Eye disorders | 35 | 1.32  (0.91 - 1.90) | 1.31  (2.14) | 1.25  (0.92) | 0.33  (-0.20) |
| Injury, poisoning and procedural complications | 34 | 1.06  (0.74 - 1.54) | 1.06  (0.11) | 1.05  (0.77) | 0.07  (-0.45) |
| Congenital, familial and genetic disorders | 26 | 1.26  (0.82 - 1.93) | 1.26  (1.13) | 1.21  (0.85) | 0.28  (-0.33) |
| Hepatobiliary disorders | 17 | 0.69  (0.42 - 1.14) | 0.69  (2.13) | 0.72  (0.47) | -0.47  (-1.19) |
| Neoplasms benign, malignant and unspecified (incl cysts and polyps) | 16 | 2.18  (1.23 - 3.88) | 2.18  (7.43) | 1.86  (1.15) | 0.89  (0.10) |
| Musculoskeletal and connective tissue disorders | 16 | 0.65  (0.39 - 1.09) | 0.65  (2.74) | 0.68  (0.44) | -0.55  (-1.28) |
| Renal and urinary disorders | 13 | 1.01  (0.56 - 1.83) | 1.01  (0.00) | 1.01  (0.62) | 0.02  (-0.81) |
| Pregnancy, puerperium and perinatal conditions | 7 | 1.78  (0.76 - 4.15) | 1.78  (1.83) | 1.60  (0.79) | 0.68  (-0.47) |
| Immune system disorders | 5 | 0.41  (0.17 - 1.02) | 0.41  (3.94) | 0.45  (0.21) | -1.15  (-2.37) |
| Endocrine disorders | 5 | 0.97  (0.38 - 2.51) | 0.97  (0.00) | 0.98  (0.44) | -0.03  (-1.30) |
| Social circumstances | 4 | 0.69  (0.24 - 1.94) | 0.69  (0.51) | 0.72  (0.3) | -0.47  (-1.84) |
| Ear and labyrinth disorders | 3 | 0.73  (0.22 - 2.43) | 0.73  (0.26) | 0.76  (0.28) | -0.39  (-1.94) |
| Reproductive system and breast disorders | 2 | 1.95  (0.39 - 9.66) | 1.95  (0.69) | 1.71  (0.45) | 0.78  (-1.15) |
| Product issues | 1 | 2.92  (0.27 - 32.26) | 2.92  (0.84) | 2.28  (0.31) | 1.19  (-1.31) |

DTaP: diphtheria, tetanus, and acellular pertussis; SOC, System Organ Class; CI, confidence interval; ROR, reporting odds ratio; PRR, proportional reporting ratio; χ^2^, Chi-squared; IC, information component; IC-2SD, the lower limit of the 95% two-sided CI of the IC; EBGM, empirical Bayesian geometric mean; EBGM05, the lower 95 two-sided CI of EBGM

# Table S9. Signal strength of all DTaP-related DME-associated PT signals and their corresponding SOCs.

| SOC | PT | a | ROR  (95%Cl) | PRR  (χ^2^) | EBGM  (EBGM05) | IC  (IC-2SD) |
| --- | --- | --- | --- | --- | --- | --- |
| Skin and subcutaneous tissue disorders | Erythema multiforme | 280 | 1.28  (1.12 - 1.47) | 1.28  (12.88) | 1.21  (1.08) | 0.27  (0.08) |
| Immune system disorders | Anaphylactic reaction | 133 | 0.85  (0.70 - 1.02) | 0.85  (3.05) | 0.87  (0.75) | -0.19  (-0.47) |
| Skin and subcutaneous tissue disorders | Angioedema | 52 | 0.70  (0.52 - 0.94) | 0.70  (5.81) | 0.74  (0.58) | -0.43  (-0.85) |
| Skin and subcutaneous tissue disorders | Dermatitis exfoliative | 25 | 1.57  (0.98 - 2.50) | 1.57  (3.58) | 1.40  (0.94) | 0.48  (-0.16) |
| Ear and labyrinth disorders | Deafness | 25 | 0.61  (0.40 - 0.93) | 0.61  (5.29) | 0.67  (0.47) | -0.58  (-1.19) |
| Immune system disorders | Anaphylactoid reaction | 23 | 0.93  (0.59 - 1.48) | 0.93  (0.09) | 0.95  (0.65) | -0.08  (-0.73) |
| Blood and lymphatic system disorders | Haemolytic anaemia | 20 | 1.39  (0.83 - 2.33) | 1.39  (1.62) | 1.29  (0.84) | 0.36  (-0.35) |
| Blood and lymphatic system disorders | Haemolysis | 18 | 1.36  (0.79 - 2.33) | 1.36  (1.24) | 1.26  (0.80) | 0.33  (-0.41) |
| Eye disorders | Blindness | 14 | 0.65  (0.37 - 1.16) | 0.65  (2.17) | 0.71  (0.44) | -0.50  (-1.30) |
| Skin and subcutaneous tissue disorders | Stevens-johnson syndrome | 14 | 0.80  (0.45 - 1.42) | 0.80  (0.60) | 0.83  (0.51) | -0.27  (-1.08) |
| Immune system disorders | Anaphylactic shock | 10 | 0.29  (0.15 - 0.55) | 0.29  (16.03) | 0.34  (0.20) | -1.55  (-2.45) |
| Gastrointestinal disorders | Intestinal perforation | 8 | 0.82  (0.38 - 1.77) | 0.82  (0.26) | 0.85  (0.45) | -0.23  (-1.28) |
| Renal and urinary disorders | Renal failure | 8 | 0.63  (0.30 - 1.33) | 0.63  (1.50) | 0.68  (0.36) | -0.55  (-1.59) |
| Blood and lymphatic system disorders | Pancytopenia | 6 | 0.52  (0.22 - 1.21) | 0.52  (2.40) | 0.57  (0.28) | -0.80  (-1.96) |
| Blood and lymphatic system disorders | Aplastic anaemia | 4 | 0.62  (0.21 - 1.77) | 0.62  (0.82) | 0.67  (0.28) | -0.58  (-1.97) |
| Respiratory, thoracic and mediastinal disorders | Pulmonary hypertension | 4 | 1.48  (0.46 - 4.71) | 1.48  (0.44) | 1.34  (0.51) | 0.42  (-1.07) |
| Hepatobiliary disorders | Hepatic failure | 3 | 0.35  (0.11 - 1.13) | 0.35  (3.38) | 0.40  (0.15) | -1.31  (-2.83) |
| Blood and lymphatic system disorders | Autoimmune haemolytic anaemia | 3 | 0.48  (0.14 - 1.6) | 0.48  (1.48) | 0.54  (0.20) | -0.88  (-2.43) |
| Skin and subcutaneous tissue disorders | Toxic epidermal necrolysis | 3 | 1.58  (0.41 - 6.12) | 1.58  (0.45) | 1.41  (0.45) | 0.49  (-1.19) |
| Renal and urinary disorders | Azotaemia | 3 | 0.69  (0.20 - 2.38) | 0.69  (0.34) | 0.74  (0.26) | -0.43  (-2.01) |
| Blood and lymphatic system disorders | Aplasia pure red cell | 3 | 5.54  (0.93 - 33.16) | 5.54  (4.47) | 2.82  (0.63) | 1.49  (-0.37) |
| Ear and labyrinth disorders | Deafness neurosensory | 3 | 0.74  (0.21 - 2.55) | 0.74  (0.23) | 0.78  (0.28) | -0.35  (-1.94) |
| Hepatobiliary disorders | Hepatic necrosis | 2 | 0.74  (0.16 - 3.37) | 0.74  (0.15) | 0.78  (0.22) | -0.35  (-2.2) |
| Hepatobiliary disorders | Acute hepatic failure | 2 | 0.67  (0.15 - 3.03) | 0.67  (0.27) | 0.72  (0.20) | -0.47  (-2.31) |
| Renal and urinary disorders | Acute kidney injury | 2 | 0.34  (0.08 - 1.43) | 0.34  (2.41) | 0.39  (0.12) | -1.35  (-3.12) |
| General disorders and administration site conditions | Sudden cardiac death | 2 | N/A  (N/A) | N/A  (7.39) | 4.69  (0) | 2.23  (-0.13) |
| Respiratory, thoracic and mediastinal disorders | Pulmonary fibrosis | 2 | 1.48  (0.29 - 7.62) | 1.48  (0.22) | 1.34  (0.34) | 0.42  (-1.53) |
| Cardiac disorders | Ventricular fibrillation | 1 | 0.62  (0.07 - 5.11) | 0.62  (0.21) | 0.67  (0.11) | -0.58  (-2.86) |
| Gastrointestinal disorders | Pancreatitis | 1 | 0.22  (0.03 - 1.63) | 0.22  (2.66) | 0.26  (0.05) | -1.94  (-4.08) |
| Ear and labyrinth disorders | Deafness permanent | 1 | 1.23  (0.13 - 11.84) | 1.23  (0.03) | 1.17  (0.18) | 0.23  (-2.18) |
| Blood and lymphatic system disorders | Granulocytopenia | 1 | 0.18  (0.02 - 1.38) | 0.18  (3.43) | 0.22  (0.04) | -2.16  (-4.29) |
| Blood and lymphatic system disorders | Agranulocytosis | 1 | 0.18  (0.02 - 1.31) | 0.18  (3.69) | 0.21  (0.04) | -2.23  (-4.36) |
| Musculoskeletal and connective tissue disorders | Rhabdomyolysis | 1 | 0.34  (0.04 - 2.6) | 0.34  (1.20) | 0.39  (0.07) | -1.35  (-3.55) |
| Hepatobiliary disorders | Hepatitis fulminant | 1 | 0.92  (0.10 - 8.26) | 0.92  (0.01) | 0.94  (0.15) | -0.09  (-2.45) |
| Blood and lymphatic system disorders | Febrile neutropenia | 1 | 0.53  (0.06 - 4.29) | 0.53  (0.37) | 0.59  (0.10) | -0.77  (-3.02) |
| Blood and lymphatic system disorders | Immune thrombocytopenia | 1 | 0.02  (0.00 - 0.16) | 0.02  (43.52) | 0.03  (0.01) | -5.18  (-7.23) |
| Ear and labyrinth disorders | Deafness transitory | 1 | 3.69  (0.23 - 59.06) | 3.69  (0.98) | 2.35  (0.23) | 1.23  (-1.40) |

DTaP, diphtheria, tetanus, and acellular pertussis; DME, designated medical event; PT, preferred term; SOC, System Organ Class; CI, confidence interval; ROR, reporting odds ratio; PRR, proportional reporting ratio; χ^2^, Chi-squared; IC, information component; IC-2SD, the lower limit of the 95% two-sided CI of the IC; EBGM, empirical Bayesian geometric mean; EBGM05, the lower 95 two-sided CI of EBGM; NA, not available.
